# Interleukin-1 Receptor Antagonist Has a Novel Function in the Regulation of Matrix Metalloproteinase-13 Expression

**DOI:** 10.1371/journal.pone.0140942

**Published:** 2015-10-16

**Authors:** Hisashi Goto, Yuichi Ishihara, Takeshi Kikuchi, Ario Izawa, Nobuaki Ozeki, Eijiro Okabe, Yosuke Kamiya, Yusuke Ozawa, Hiroki Mizutani, Genta Yamamoto, Makio Mogi, Kazuhiko Nakata, Hatsuhiko Maeda, Toshihide Noguchi, Akio Mitani

**Affiliations:** 1 Department of Periodontology, School of Dentistry, Aichi Gakuin University, Chikusa-ku, Nagoya, Aichi, Japan; 2 Department of Operative Dentistry, Endodontology, and Periodontology, School of Dentistry, Matsumoto Dental University, Nagano, Japan; 3 Department of Endodontics, School of Dentistry, Aichi Gakuin University, Chikusa-ku, Nagoya, Aichi, Japan; 4 Department of Medicinal Biochemistry, School of Pharmacy, Aichi Gakuin University, Chikusa-ku, Nagoya, Japan; 5 Department of Pathology, School of Dentistry, Aichi Gakuin University, Chikusa-ku, Nagoya, Japan; National Center for Scientific Research Demokritos, GREECE

## Abstract

Interleukin-1 receptor antagonist (IL-1Ra) is an IL-1 family member, which binds to IL-1 receptors but does not induce any intracellular signaling. We addressed whether IL-1Ra has a novel function in regulation of the extracellular matrix or adhesion molecules. Polymerase chain reaction array analysis demonstrated a ~5-fold increase in matrix metalloproteinase 13 (MMP-13) mRNA expression of IL-1Ra siRNA-transfected Ca9-22 human oral squamous epithelial carcinoma cells compared with the control. In fact, MMP-13 mRNA and protein expression as well as its activity in IL-1Ra siRNA-transfected Ca9-22 cell lines were significantly higher than those in the control. IL-1Ra siRNA treatment resulted in strong elevation of MMP-13 expression, whereas addition of rhIL-1Ra (40 ng/ml) suppressed MMP-13 expression, suggesting that IL-1Ra had a specific effect on MMP-13 induction. IL-1Ra siRNA could potently suppress IL-1α. No significant difference was found between the MMP-13 mRNA expression of IL-1Ra siRNA-transfected cells and those treated with anti-IL-1α or anti-IL-1β antibodies. These results suggested that continuous supply of IL-1 had no effect on the induction of MMP-13 by IL-1Ra siRNA. Histopathological investigation of MMP-13 in periodontal tissue showed specific localization in the junctional epithelial cells of IL-1Ra knockout (KO) mice. Furthermore, infection with *Aggregatibacter actinomycetemcomitans* to establish an experimental periodontitis model resulted in predominant localization of MMP-13 along apical junctional epithelial cells. Laminin-5, which is degraded by MMP-13, was found in the internal basal lamina of wild-type mice, whereas the internal basal lamina of IL-1Ra KO mice did not show obvious laminin-5 localization. In particular, laminin-5 localization almost disappeared in the internal basal lamina of IL-1Ra KO mice infected with *A*. *actinomycetemcomitans*, suggesting that the suppression of IL-1Ra resulted in strong induction of MMP-13 that degraded laminin-5. In conclusion, IL-1Ra is associated with MMP-13 expression and has a novel function in such regulation without interference of the IL-1 signaling cascade.

## Introduction

Interleukin (IL)-1 is closely associated with inflammatory tissue destruction in rheumatoid arthritis and periodontitis [1, 2]. Clinical studies indicate elevation of IL-1β and IL-1α levels in gingival tissue and gingival crevicular fluid (GCF) from periodontitis patients compared with healthy subjects [3, 4]. IL-1 receptor antagonist (IL-1Ra) is a naturally occurring inhibitor of the IL-1 receptor by binding competitively to the receptor without transduction of signaling [5]. IL-1Ra levels in GCF are negatively correlated with the disease severity of chronic periodontitis patients [3]. In addition, IL-1Ra levels in moderate and deep pockets of patients with aggressive periodontitis are lower than those at control sites [6]. Neutralizing anti-IL-1Ra antibodies have demonstrated that endogenous IL-1Ra is an important anti-inflammatory protein in arthritis, colitis, and granulomatous pulmonary disease [7]. IL-1Ra knockout (KO) mice with a BALB/ca background spontaneously develop chronic inflammatory polyarthropathy that closely resembles arthritis in humans [8]. Histopathological analysis has revealed marked synovial and periarticular inflammation that is accompanied by articular erosion caused by invasion of granulation tissue in these mice [9]. Because infection with *Aggregatibacter actinomycetemcomitans* establishes an experimental periodontitis model, *A*. *actinomycetemcomitans* lipopolysaccharide (LPS)-stimulated culture supernatants from IL-1Ra KO mouse peritoneal macrophages induce severe calvarial bone resorption compared with those from wild-type (WT) mice [10]. Because IL-1Ra gene deficiency promotes up-regulation and down-regulation of osteoclast- and osteoblast-related gene expression, respectively, such dysregulation induces an imbalance in bone formation and resorption [10, 11]. Live *A*. *actinomycetemcomitans*-infected IL-1Ra KO mice as an experimental periodontitis model show histological apical migration of the junctional epithelium as well as epithelial attachment loss in periodontal tissue [11].

The junctional epithelium, a unique type of epithelium that forms the dento-epithelial junction, adheres to teeth by hemidesmosomes in the internal basal lamina [12]. Hemidesmosomes are thought to be composed of laminin-5 and integrin α_6_β_4_ [12]. Laminin-5 has been identified in the internal basal lamina of the junctional epithelium by immunohistochemistry and in situ hybridization [13–15]. Matrix metalloproteinases (MMPs) are a family of enzymes that collectively degrade the components of the extracellular matrix. MMPs have crucial roles in normal physiological processes such as development and wound healing [16]. It has been reported that IL-1 induces MMP-13 in osteoblasts and fibroblasts. Moreover, all collagenases, particularly MMP-13, are able to cleave extracellular matrix components such as the laminin-5 γ2-chain of the gingival basement membrane [17, 18]. However, it is unknown whether IL-1Ra has a protective effect on the apical migration and loss of attachment of the junctional epithelium. Therefore, we hypothesized that IL-1Ra is an important endogenous protein in regulation of the extracellular matrix or cell adhesion molecules.

In the present study, IL-1Ra gene expression in a human gingival epithelial cell line (Ca9-22) was knocked down by small interfering RNA (siRNA). The gene expression profiles of 84 genes involved in regulation of the extracellular matrix and adhesion molecules were then compared between IL-1Ra KO and normal Ca9-22 cells by polymerase chain reaction (PCR) array analysis. Moreover, protein localization of MMP-13, which was up-regulated by IL-1Ra KO in Ca9-22 cells, was examined by immunohistochemistry in an experimental periodontitis model of IL-1Ra KO mice. Here, we demonstrate that IL-1Ra has a novel function in the regulation of the extracellular matrix and adhesion molecules.

## Materials and Methods

### Cell culture

Ca9-22 cells, a human oral squamous epithelial carcinoma cell line [19, 20], were commercially available and obtained from the RIKEN Bioresource Center (Ibaraki, Japan). The cells were cultured in Dulbecco’s modified Eagle’s medium (Gibco-BRL, Grand Island, NY) containing 10% fetal bovine serum (Hyclone Laboratories, Inc., Logan, UT) and an antibiotic solution (100 U/ml penicillin and 100 μg/ml streptomycin, Gibco-BRL) in a humidified atmosphere with 5% CO_2_ at 37°C. Passage 4–6 Ca9-22 cells were subjected to siRNA transfection assays.

### Transfection of siRNA

Ca9-22 cells were seeded at 1 × 10^6^ cells/well in six-well plates. After 24 hours, the cells were transiently transfected with either 20 nM siRNA targeting IL-1Ra (Stealth Select RNAi, Invitrogen, Carlsbad, CA) or control siRNA (Invitrogen) using Lipofectamine RNAiMAX (Invitrogen) according to the manufacturer’s protocol. After 48 hours, the transfected cells were harvested to examine cell growth, morphology, mRNA expression, protein synthesis, and gene expression profiles using a PCR array.

### Real-time PCR

After transfection, total RNA was extracted immediately using the NucleoSpin RNA II system (Macherey-Nagel Inc., Bethlehem, PA) according to the manufacturer’s instructions. We analyzed IL-1α, IL-1β, IL-1Ra, MMP-13, tissue inhibitor of metalloproteinase (TIMP)-1, and TIMP-2 mRNA expression at 1, 3, 6, 12, and 24 hours. The quality of the RNA samples was assessed by calculating A230/A260 and A260/A280 ratios using a Nano-Drop ND-1000 fluorospectrometer (Thermo Scientific, Wilmington, DE). cDNA was synthesized from total RNA using random primers and 200 U SuperScript III reverse transcriptase (Invitrogen). To quantify mRNA expression, quantitative PCR analyses were performed using TaqMan gene expression assays (Applied Biosystems, Foster City, CA) for human IL-1α (Hs00174092_m1), IL-1β (Hs01555410_m1), IL-1Ra (Hs00893626_m1), MMP-13 (Hs00233992_m1), TIMP-1 (Hs00171558_m1), and TIMP-2 (Hs00234278_m1) with TaqMan Universal PCR master mix (Applied Biosystems). mRNA levels were normalized to the level of eukaryotic 18S rRNA (Hs99999901_s1). Quantitative PCR was performed using the ABI Prism 7000 Sequence Detection System with the associated software (version 1.0; Applied Biosystems). PCR conditions were 10 minutes at 95°C, followed by 40 cycles of 15 seconds at 95°C and 1 minute at 60°C. The relative amounts of target mRNAs were determined by subtracting the cycle threshold (CT) value for the gene from that for 18S rRNA (ΔCT). Then, the ΔCT value for the control group was subtracted from that for the experimental group (ΔΔCT). The results are expressed as the fold change (2^-ΔΔCT^) between the mRNA expression levels of control and experimental groups, where ΔΔCT is calculated as follows: [(CT for the target mRNA − CT for 18S rRNA) for the experimental group] − [(CT for the target mRNA − CT for 18S rRNA) for the control group].

### PCR array analysis

For PCR array analysis, after 6 hours of culture, cDNA of Ca9-22 cells transfected with IL-1Ra or control siRNAs was prepared using an RT^2^ First Strand Kit (SABiosciences, Frederick, MD) according to the manufacturer’s protocol and applied to a Human Extracellular Matrix and Adhesion Molecules RT^2^Profile PCR Array (SABiosciences). Amplification was carried out using RT^2^ SYBR Green/ROX qPCR Master Mix and the 7000 Real-Time PCR System. Relative gene expression values were analyzed using a web-based software package to calculate ΔΔCT-based fold changes (http://www.sabiosciences.com/pcrarraydataanalysis.php).

### Western blot analysis

After 48 hours of culture, Ca9-22 cells transfected with IL-1Ra or control siRNAs were lysed with CellyticM lysis buffer (Sigma-Aldrich, St. Louis, MO) containing a protease inhibitor cocktail (Nacalai Tesque, Kyoto, Japan) and phosphatase inhibitor cocktail (Nacalai Tesque). Proteins were separated by any kDa sodium dodecyl sulfate polyacrylamide gel electrophoresis (Bio-Rad Laboratories, Hercules, CA) and transferred to polyvinylidene fluoride membranes (Bio-Rad Laboratories).

The primary antibodies were a mouse anti-human IL-1Ra monoclonal antibody (Cell Signaling Technologies, Beverly, MA), rabbit anti-human MMP-13 polyclonal antibody (Santa Cruz Biotechnology Inc., Santa Cruz, CA), mouse anti-human TIMP-1 and TIMP-2 polyclonal antibodies (Santa Cruz Biotechnology Inc., Santa Cruz, CA, USA), and a mouse anti-human β-actin monoclonal antibody (Cell Signaling Technologies). β-Actin was used as a loading control. The secondary antibody was a horse anti-mouse IgG (Cell Signaling Technologies) to detect IL-1Ra and TIMP-1, goat anti-rabbit IgG (Santa Cruz Biotechnology) to detect MMP-13, and donkey anti-goat IgG (Santa Cruz Biotechnology Inc.) to detect TIMP-2.

### Measurement of MMP-13 activity

Activity of the endogenous active form of MMP-13 was measured using a commercially available MMP-13 activity assay kit (SensoLyte® Plus 520 MMP-13 assay kit, AnaSpec, San Jose, CA). IL-1Ra and control siRNA-transfected cell culture supernatants were collected after 24 hours of culture. Then, MMP-13 activity was determined according to the manufacturer’s instructions.

### Recombinant human IL-1Ra treatment

IL-1Ra siRNA-transfected cells were cultured for 6 hours in the presence of recombinant human (rh)IL-1 Ra (40 ng/ml, PeproTech, Rocky Hill, NJ). Then, the total RNA of the cells was collected to investigate MMP-13 mRNA expression.

### Enzyme-linked immunosorbent assays

IL-1Ra and control siRNA-transfected cells were cultured for 24 hours, and then the culture supernatants were collected to detect the levels of IL-1α and IL-1β. We used Quantikine human IL-1α and IL-1β enzyme-linked immunosorbent assay (ELISA) kits (R&D Systems, Minneapolis, MN) according to the manufacturer’s instructions. The minimum detectable concentration was 1 pg/ml.

### Neutralization of IL-1

To neutralize IL-1 in IL-1Ra siRNA-transfected cells, we applied 1 μg/ml anti-IL-1α, anti-IL-1β, or isotype control antibodies (R&D Systems). After 6 hours, the total RNA of the cells was collected to investigate MMP-13 mRNA expression.

### IL-1Ra KO and WT mice

IL-1Ra KO mice with a BALB/ca background [8] were kindly provided by Dr. Y. Iwakura (Tokyo University of Science, Chiba, Japan). Control WT BALB/cA mice were purchased from CLEA Japan Inc. (Tokyo, Japan). All mice were maintained under specific pathogen-free conditions at Aichi Gakuin University, Japan, and provided standard mouse chow and water *ad libitum*. To minimize animal suffering and prevent aspiration, each mouse was orally infected with a total daily dose in several batches. No animals experienced clinical symptoms, and we provided veterinary care including promotion of the animal’s well-being such as sanitation, nutrition and parasite control. The condition of the animals was monitored twice a day. Particularly on the day of oral infection, we monitored the condition of the animals carefully. The mice were euthanized by cervical dislocation under sodium pentobarbital anesthesia. This study was specifically approved and carried out in strict accordance with the recommendations of the Guide for the Care and Use of the Animal Research Ethics Committee of Aichi Gakuin University (Permit number: AGUD 271).

### *A*. *actinomycetemcomitans* culture

*A*. *actinomycetemcomitans* (ATCC29524) cells were grown in brain–heart infusion medium (Difco Laboratories, Detroit, MI) with 1% yeast extract (Difco Laboratories) at 37°C in a CO_2_-rich atmosphere with an AnaeroPack (Mitsubishi Gas Chemical Co., Inc., Tokyo, Japan).

### Experimental periodontitis induced by *A*. *actinomycetemcomitans*

IL-1Ra KO and WT mice (four mice per group) were infected with 300 μl of 1 × 10^10^ colony-forming units (CFUs)/ml live *A*. *actinomycetemcomitans*. Each mouse was orally infected with 3 × 10^9^ CFUs suspended in sterile phosphate-buffered saline (PBS) containing 2.5% carboxymethylcellulose for a total of five times at 1-day intervals.

### Immunohistochemistry

At 42 days after the last infection, mandibles were removed and immediately fixed in 4% paraformaldehyde overnight at 4°C. Then, the mandible specimens were decalcified with a 10% EDTA solution (pH 7.6) (Muto Pure Chemicals Co., Ltd., Tokyo, Japan) for 21 days at 4°C. Next, paraffin embedding was performed and serial histological sections (buccolingual, 4 μm thick) were prepared. Rabbit polyclonal antibodies against mouse MMP-13 or laminin-5 (Abcam Inc., Cambridge, MA) diluted at 1:50 or 1:200, respectively, in 1% bovine serum albumin/0.01 M PBS were applied to the sections. The secondary antibody was Histofine simple stain mouse MAX-PO (R) (Nichirei, Tokyo, Japan) and applied according to the manufacturer’s instructions. The stained sections were observed under a light microscope.

### MMP-13 expression in cells infected with *A*. *actinomycetemcomitans* in the epithelial gingival layer

After 28 days of *A*. *actinomycetemcomitans* infection, mouse molars and the buccal gingival epithelial layer were harvested and homogenized in PBS containing a complete EDTA-free protease inhibitor cocktail (Roche Diagnostics, Mannheim, Germany) and 0.05% Triton X-100 by ultrasonic fragmentation using a Microson XL-2000 (Misonix, Farmingdale, NY). Homogenized samples were centrifuged at 13000 *g* for 10 minutes at 4°C, and the supernatants were collected. MMP-13 in supernatants was measured using an ELISA kit (Cloud-Clone Corp., Houston, TX) according to the manufacturer’s instructions. The minimum detectable concentration was 78 pg/ml.

### Statistical analysis

Data were analyzed using PASW Statistics software (version 18.0; SPSS Japan, Tokyo, Japan). Differences among groups were examined by one-factor analysis of variance (ANOVA) and Bonferroni’s multiple comparison test. Comparisons of two independent groups were performed using the Student’s *t*-test. Data are expressed as the mean ± standard deviation (SD). Significance was accepted at *p* < 0.05.

## Results

### PCR array analysis of genes associated with the extracellular matrix and adhesion molecules in IL-1Ra siRNA-transfected cells

**Fig 1:**
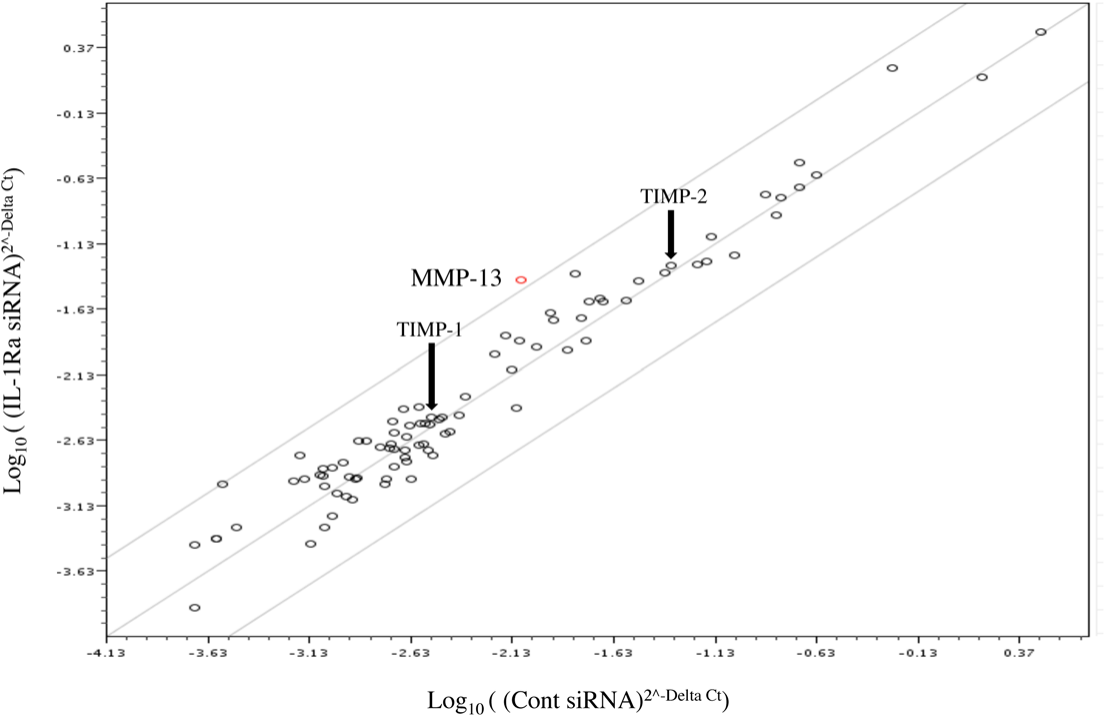
Investigation of the expression of genes involved in cell–cell and cell–matrix interactions using a PCR array. The graph shows the fold changes of gene expression in IL-1Ra and control siRNA-transfected Ca9-22 cells. MMP-13 was up-regulated in IL-1Ra siRNA-transfected cells by 4.8-fold (n = 6). The red circle (most up-regulated gene) indicates MMP-13 expression in the graph. TIMP-1 and TIMP-2 expression levels are indicated by arrows.

A human extracellular matrix and adhesion molecules PCR array was used to investigate differences in the expression of 84 genes involved in cell–cell and cell–matrix interactions. Fig 1 shows the fold changes in expression between control and IL-1Ra siRNA-transfected cells after incubation for 6 hours. Among the 84 genes, MMP-13 mRNA expression (indicated by the red circle) was up-regulated by 4.8-fold in IL-1Ra siRNA-transfected cells compared with control siRNA-transfected cells. MMP-13 activity is precisely regulated after its secretion at the post-translational level as a precursor zymogen before activation and endogenous tissue inhibitors of metalloproteinases referred to as TIMPs. Although studies have already shown that TIMP-2 is induced by cytokines, TIMP-1 and TIMP-2 expression levels (indicated by arrows) were unchanged in IL-1Ra siRNA-transfected cells compared with the control.

### Transfection of IL-1Ra siRNA into Ca9-22 cells

**Fig 2:**
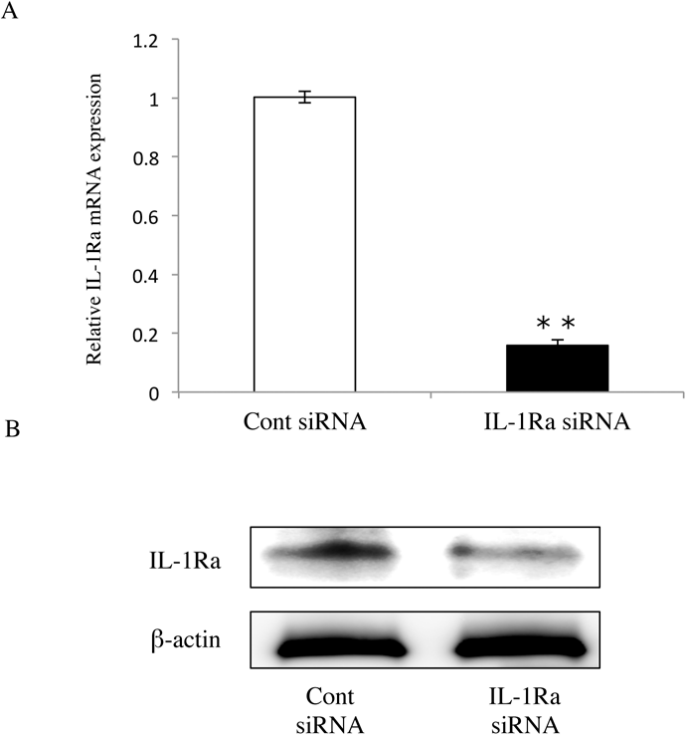
Suppression of IL-1Ra expression using siRNA. (A) Determination of IL-1Ra mRNA levels using real-time PCR. At 48 hours after transfection, IL-1Ra siRNA-transfected cells showed clear knockdown of IL-1Ra mRNA compared with the control. Differences among groups were analyzed using the Student’s *t*-test. Data are expressed as the mean ± SD (n = 6). **p < 0.01 vs control. (B) Western blot of IL-1Ra (18 KDa) in cells transfected with either IL-1Ra or control siRNAs (n = 3). Data are representative of three independent experiments.

IL-1Ra siRNA-transfected Ca9-22 cells showed obvious knockdown of IL-1Ra mRNA compared with control siRNA-transfected Ca9-22 cells (Fig 2A). Western blot analysis confirmed a decrease in the protein expression of IL-1Ra (18 kDa) in IL-1Ra siRNA-transfected Ca9-22 cells compared with the control (Fig 2B). These results indicated that transfection of IL-1Ra siRNA clearly knocked down the mRNA and protein expression of IL-1Ra in Ca9-22 cells.

### Expression of MMP-13 in IL-1Ra siRNA-transfected cells

**Fig 3:**
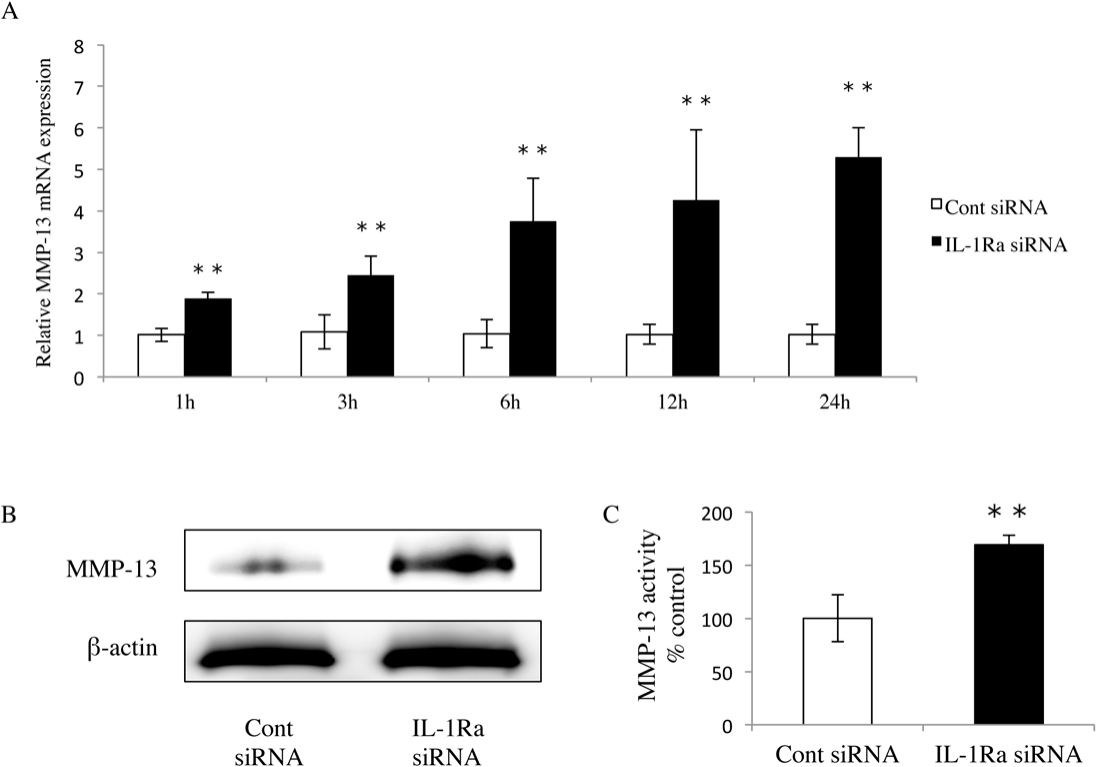
Up-regulation of MMP-13 by IL-1Ra siRNA. (A) MMP-13 mRNA expression in IL-1Ra and control siRNA-transfected cells. The cells were cultured for 1, 3, 6, 12, and 24 hours, and then mRNA levels were determined using real-time PCR. Values represent fold changes. Differences among groups were analyzed using the Student’s *t*-test. Data are expressed as the mean ± SD (n = 6). **p < 0.01 vs each time point control. (B) Western blot of MMP-13 (60 kDa) in cells transfected with either IL-1Ra or control siRNAs. Data are representative of three independent experiments. (C) Activity of the endogenous active form of MMP-13 was measured by a SensoLyte® Plus 520 MMP-13 assay. Differences among groups were analyzed using the Student’s *t*-test. Data are expressed as the mean ± SD (n = 3). **p < 0.01 vs control.

At 1, 3, 6, 12, and 24 hours, the MMP-13 mRNA expression of IL-1Ra siRNA-transfected cells was significantly higher than that of control siRNA-transfected cells (Fig 3A). Western blot analysis confirmed high protein expression of MMP-13 (60 kDa) in IL-1Ra siRNA-transfected cells compared with the control (Fig 3B). These results demonstrated that transfection of IL-1Ra siRNA strongly induced both mRNA and protein expression of MMP-13 in Ca9-22 cells.

### MMP-13 activity in IL-1Ra siRNA-transfected cells

By analyzing the endogenous active form of MMP-13 using an MMP-13 activity assay, we found that the activity of MMP-13 in IL-1Ra siRNA-transfected cells was higher than that in control siRNA-transfected cells (Fig 3C).

### TIMP expression in IL-1Ra siRNA-transfected cells

**Fig 4:**
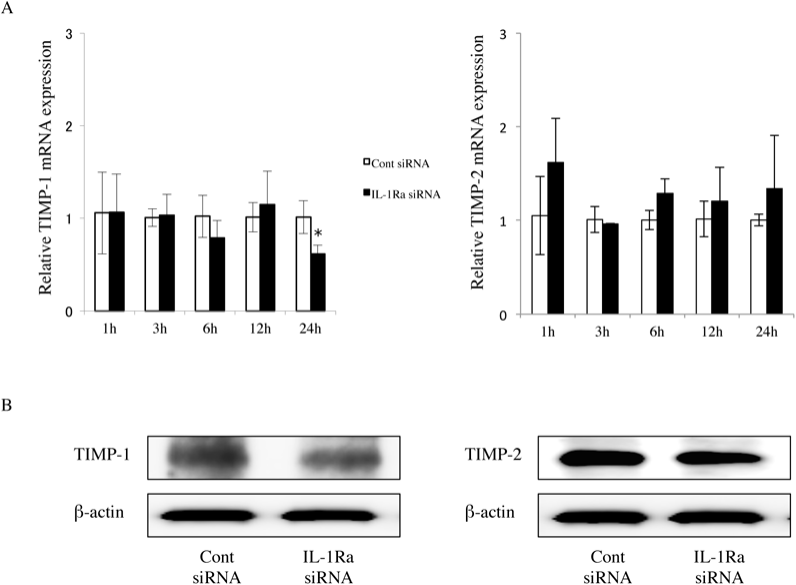
TIMP expression in IL-1Ra siRNA-transfected cells. (A) TIMP-1 and TIMP-2 mRNA expression in IL-1Ra and control siRNA-transfected cells. The cells were cultured for 1, 3, 6, 12, and 24 hours, and then mRNA levels were determined using real-time PCR. Values represent fold changes. Differences among groups were analyzed using the Student’s *t*-test. Data are expressed as the mean ± SD (n = 3). *p < 0.05 vs each time point control. (B) Western blot of TIMP-1 (23 kDa) and -2 (21 kDa) in cells transfected with either IL-1Ra or control siRNAs. Data are representative of three independent experiments.

MMP-13 activity is precisely regulated after its secretion at the post-translational level as a precursor zymogen and by TIMPs [21, 22]. Although it is known that TIMP-2 is inducible by cytokines [21], we found that TIMP-1 and TIMP-2 mRNA levels were constitutively expressed in IL-1Ra and control siRNA-transfected cells at 1, 3, 6, 12, and 24 hours (Fig 4A). These data were supported by the gene array results shown in Fig 1. To normalize the increase in MMP activity, it is necessary to describe it in the context of TIMP protein expression. As shown in Fig 1, TIMP-1 (23 kDa) and TIMP-2 (21 kDa) proteins were constitutively expressed at uniform levels under all experimental conditions (Fig 4B).

### Up-regulation of MMP-13 by IL-1Ra siRNA can be reversed by addition of rhIL-1Ra

**Fig 5:**
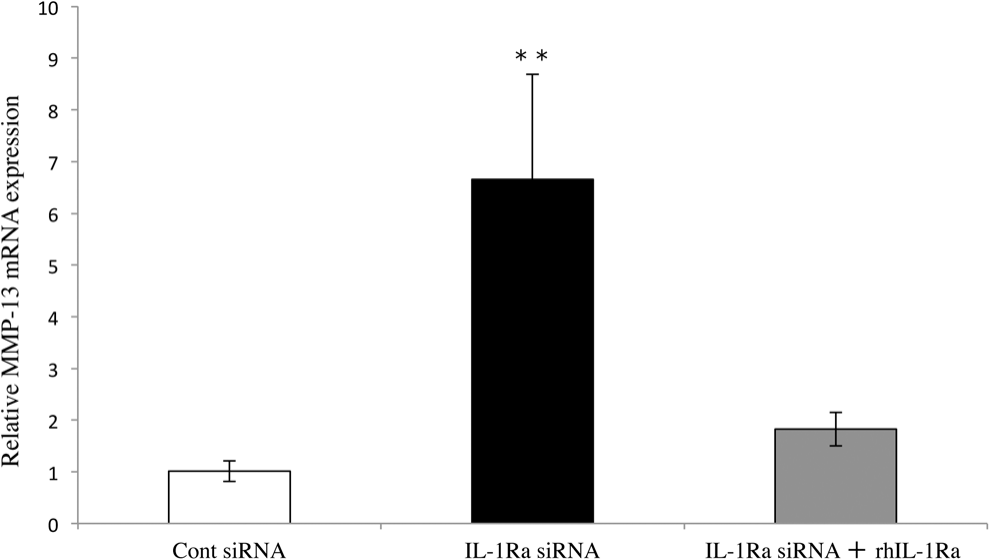
Down-regulation of MMP-13 expression by rhIL-1Ra. MMP-13 mRNA expression in IL-1Ra and control siRNA-transfected cells treated with or without rIL-1Ra. The cells were cultured for 6 hours, and then MMP-13 mRNA levels were determined using real-time PCR. Values represent fold changes. Differences among groups were analyzed by one-factor ANOVA and Bonferroni’s multiple comparison test. Data are expressed as the mean ± SD (n = 3). **p < 0.01 vs control and IL-1Ra siRNA + rIL-1Ra 40 ng/ml.

Although IL-1Ra siRNA treatment resulted in strong elevation of MMP-13 expression, addition of rhIL-1Ra (40 ng/ml) inhibited such up-regulation of MMP-13 under similar conditions (Fig 5). This result indicated that IL-1Ra has a specific function in the induction of MMP-13 in Ca9-22 cells.

### Continuous production of IL-1α but not IL-1β in Ca9-22 cells

**Fig 6:**
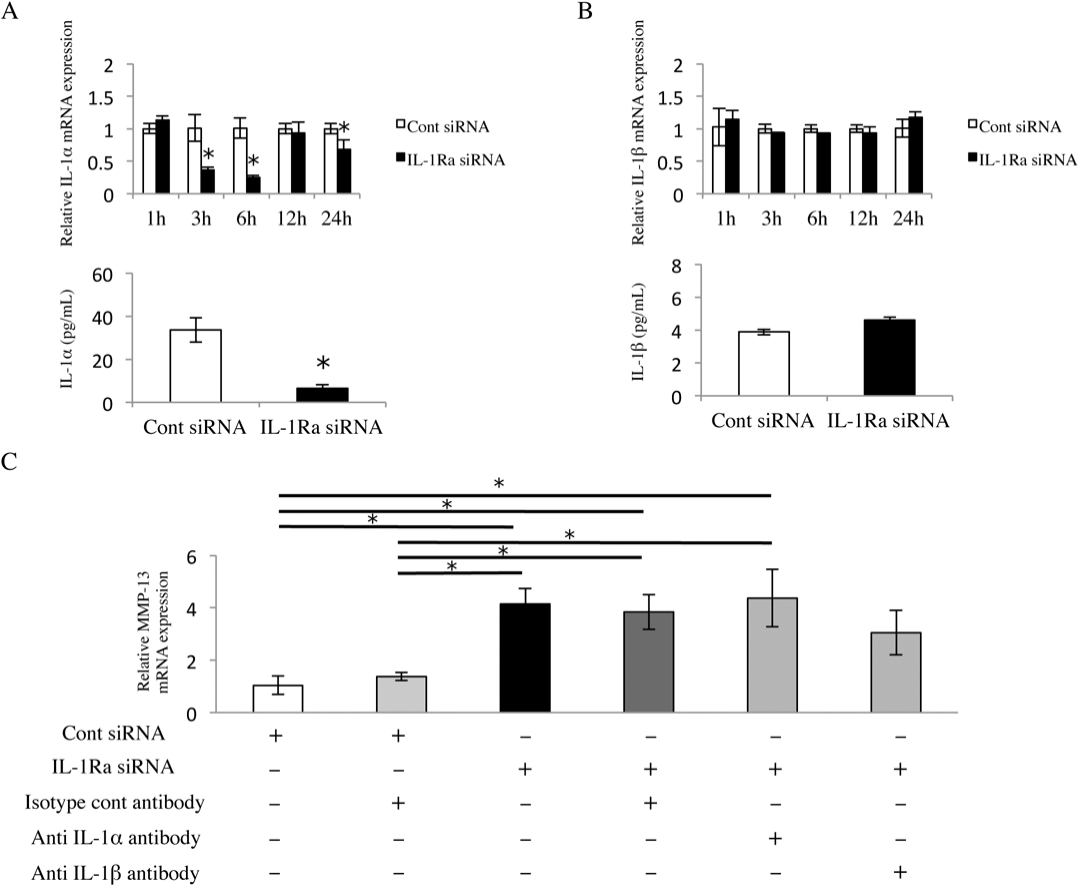
Neutralization of IL-1 does not affect MMP-13 up-regulation. (A) IL-1α and (B) IL-1β mRNA (upper column) and protein expression (lower column) in IL-1Ra siRNA-transfected cells. The cells were cultured for 1, 3, 6, 12, and 24 hours, and then analyzed by real-time PCR. The protein levels in cells cultured for 24 hours were determined by an ELISA. Values represent fold changes (real-time PCR) or concentrations (ELISA). Differences among groups were analyzed using the Student’s *t*-test. Data are expressed as the mean ± SD (n = 3). *p < 0.05 vs each time point control. (C) MMP-13 mRNA expression in IL-1Ra and control siRNA-transfected cells treated with or without anti-IL-1α, anti-IL-1β, or isotype control antibodies (1 μg/ml). Cells were cultured for 6 hours, and then mRNA levels were determined using real-time PCR. Values represent fold changes. Differences among groups were analyzed by one-factor ANOVA and Bonferroni’s multiple comparison test. Data are expressed as the mean ± SD (n = 3). *p < 0.05.

IL-1α and IL-1β induce MMP-13 production via the IL-1 receptor [23, 24]. We found continuous production of IL-1α (35 pg/mL) and IL-1β (4 pg/mL) in Ca9-22 cells (Fig 6A and 6B). By examining whether IL-1Ra can regulate IL-1 production in these cells, we found that IL-1Ra siRNA resulted in a strong but transient decrease in only IL-1α production (Fig 6A), indicating that IL-1Ra regulates the production of IL-1α. It is likely that these proinflammatory cytokines had no effect on MMP-13 production under similar conditions.

### Effect of IL-1 neutralization on MMP-13 induced by IL-1Ra deficiency

Because we found continuous production of IL-1α in Ca9-22 cells (Fig 6B), we determined whether endogenous IL-1α bound to the IL-1 receptor and induced MMP-13 under IL-1Ra siRNA treatment. There was no significant difference between the MMP-13 mRNA expression of IL-1Ra siRNA-transfected cells and those treated with an anti-IL-1α antibody. Interestingly, there was also no significant difference between MMP-13 mRNA expression levels in IL-1Ra siRNA-transfected cells and those treated with the IL-1β antibody (Fig 6C). These results suggested that continuous production of IL-1α and IL-1β had no effect on the induction of MMP-13 under IL-1Ra siRNA treatment.

### Expression and localization of MMP-13 in experimental periodontitis of IL-1Ra KO mice

**Fig 7:**
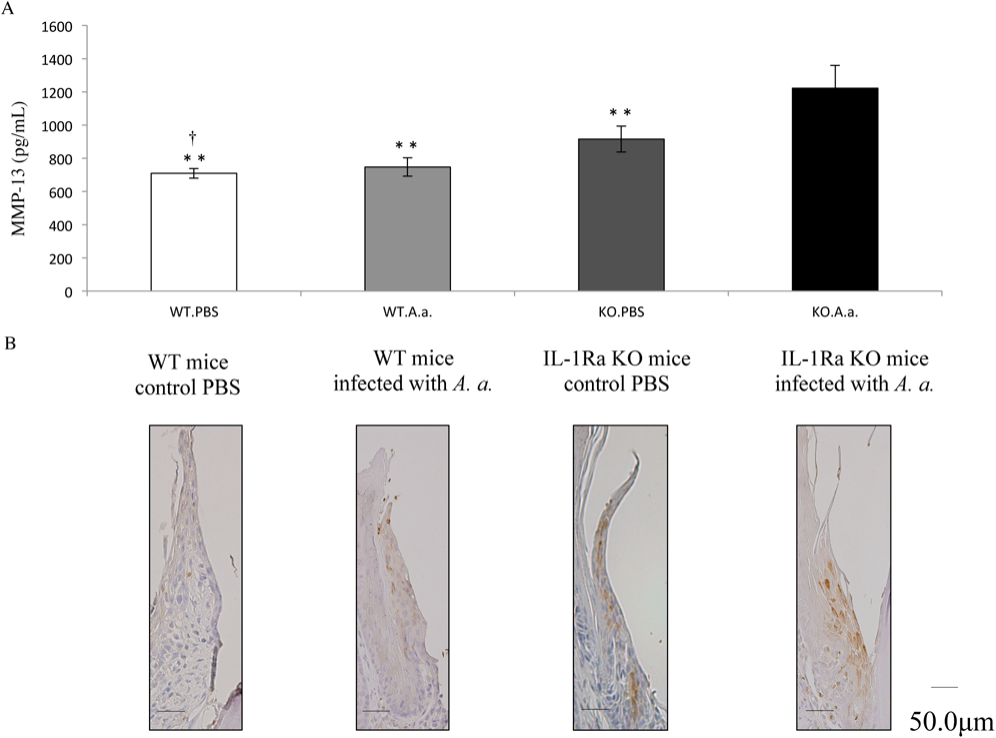
Expression and localization of MMP-13 in periodontal tissue of IL-1Ra KO and WT mice. (A) At 4 weeks after infection, the mouse gingival epithelial layer was collected to examine MMP-13 levels. The levels of MMP-13 were measured by an ELISA. Differences among groups were analyzed by one-factor ANOVA and Bonferroni’s multiple comparison test. Data are expressed as the mean ± SD (n = 4). **p <0.01 vs IL-1Ra KO mice infected with *A*. *actinomycetemcomitans*. ^†^ *p* <0.05 vs IL-1Ra KO mice treated with PBS. (B) Localization of MMP-13 in periodontal tissue. Buccolingual sections of the second and third mandibular molars from *A*. *actinomycetemcomitans*-infected IL-1Ra KO and WT mice were stained immunohistochemically. In contrast to infected WT mice, immunohistochemical findings in periodontal tissues indicated considerable inflammatory reactions in IL-1Ra KO mice infected with *A*. *actinomycetemcomitans*.

The levels of MMP-13 protein in the mouse gingival epithelial layer after 28 days of infection with *A*. *actinomycetemcomitans* were measured by ELISA. Collected tissue from IL-1Ra KO mice infected with *A*. *actinomycetemcomitans* had significantly high amounts of MMP-13 protein compared with the control (Fig 7A). MMP-13 expression in IL-1Ra KO mice treated with PBS was higher than that in WT mice treated with PBS. Histopathological investigation of MMP-13 in periodontal tissue showed specific localization of MMP-13 in the junctional epithelial cells of IL-1Ra KO mice (Fig 7B). Moreover, in contrast to KO mice treated with PBS, localization of MMP-13 in IL-1Ra KO mice infected with *A*. *actinomycetemcomitans* was mainly along apical junctional epithelial cells (Fig 7B).

### Localization of laminin-5 in experimental periodontitis of IL-1Ra KO mice

**Fig 8:**
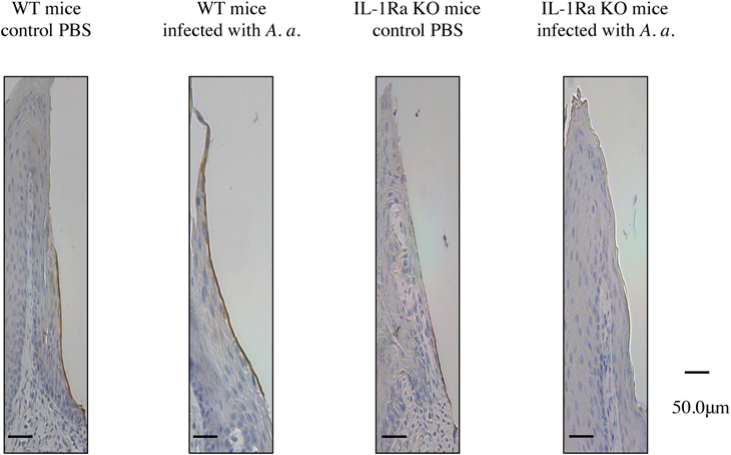
Localization of laminin-5 in periodontal tissue of IL-1Ra KO and WT mice. Buccolingual sections of the second and third mandibular molars from *A*. *actinomycetemcomitans*-infected IL-1Ra KO and WT mice were stained immunohistochemically. In contrast to IL-1Ra KO mice, positive reactions were evident in the internal basal lamina of WT mice.

MMP-13 cleaves extracellular matrix components such as laminin-5 γ2 chain of the gingival basement membrane [17]. In accordance with the distribution of MMP-13 in IL-1Ra KO mice (Fig 7B), laminin-5 was clearly found in the internal basal lamina of WT mice. However, the internal basal lamina of IL-1Ra KO mice did not show obvious localization of laminin-5. Notably, laminin-5 had almost disappeared in the internal basal lamina of IL-1Ra KO mice infected with *A*. *actinomycetemcomitans* (Fig 8). This result suggested that IL-1Ra deficiency caused MMP-13 induction and led to degradation of laminin-5 in epithelial cells.

## Discussion

Normally, IL-1Ra interferes with the IL-1 signaling cascade and prevents IL-1-induced MMP-13 expression. However, we found that IL-1Ra has a novel function to suppress MMP-13 expression.

By investigating the effect of IL-1Ra on the junctional epithelium that attaches to the enamel surface via hemidesmosomes in which laminin-5 and integrin α_6_β_4_ are the main components [12], we found that the most affected gene associated with the extracellular matrix and adhesion molecules was MMP-13 under anti-IL-1Ra siRNA treatment (Figs 1 and 3). MMP-13 functions in the loss of epithelial attachment by degradation of the laminin-5 γ2 chain in the extracellular matrix [18]. Although we have no definite data on the mechanism of MMP-13 suppression by IL-1Ra, we demonstrated that IL-1Ra plays an important role in the suppression of MMP-13 without interference of the IL-1 signaling cascade.

We found continuous production of IL-1α (35 pg/mL) and IL-1β (4 pg/mL) in Ca9-22 cells, whereas the expression and secretion of IL-1α but not IL-1β were decreased by IL-1Ra siRNA. Because Somm et al reported that the circulating levels of IL-1β are not significantly different in IL-1Ra KO and WT mice [25], it is likely that IL-1Ra does not modulate proinflammatory IL-1β. However, our previous report showed that peritoneal macrophages from IL-1Ra KO mice significantly induce IL-1α and IL-1β compared with those from WT mice [10]. Furthermore, IL-1Ra suppresses LPS-induced IL-1β production and plays a protective role against bacterial infection [26]. These discrepancies in the findings might be caused by the distinct experimental animals or the conditions of harvesting macrophages from the peritoneal cavity with thioglycollate medium.

The addition of neutralizing antibodies against IL-1α and IL-1β did not significantly affect the expression of MMP-13, suggesting that MMP-13 up-regulation induced by IL-1Ra suppression is not attributable to endogenous IL-1. Taken together, such a phenomenon implies a possible novel agonistic function of IL-1Ra. This hypothesis is also supported by the fact that a substantial amount of IL-1α was constitutively present, and that anti-IL-1Ra siRNA reduced the expression of IL-1α. It remains to be elucidated whether IL-1Ra has a specific function mediated by the IL-1 receptor. If this is the case, IL-1Ra behaves as both an agonist and antagonist to suppress MMP-13 induction.

Using an in vivo model, we investigated the expression of laminin-5 that is assumed to be cleaved by MMP-13. Moreover, *A*. *actinomycetemcomitans*-infected IL-1Ra KO mice as an experimental model of periodontitis were employed to investigate laminin-5 alterations under infectious conditions in periodontal tissue. The level of MMP-13 in collected tissue from IL-1Ra KO mice infected with *A*. *actinomycetemcomitans* was the highest among the groups, whereas MMP-13 expression in IL-1Ra KO mice treated with PBS was higher than that in WT mice treated with PBS. In addition, MMP-13 was strongly expressed in the gingival epithelial cells of mandible gingiva sections from IL-1Ra KO mice. The absence of IL-1Ra increased MMP-13 expression in vivo, demonstrating regulation of the homeostasis of periodontal tissue by IL-1Ra. This result was supported by the fact that expression of laminin-5 was not clearly observed in the periodontal tissue of IL-1Ra KO mice. In addition, MMP-13 expression was mainly induced at the apical side in IL-1Ra KO mice by *A*. *actinomycetemcomitans* infection. Thus, the absence of laminin-5 in tissue sections was likely because of the increase in MMP-13 expression. Furthermore, our results suggested that exacerbation of inflammatory responses in periodontal disease induced by *A*. *actinomycetemcomitans* infection in the absence of IL-1Ra further promoted the loss of adhesion. Indeed, *A*. *actinomycetemcomitans*-infected IL-1Ra KO mice undergo rapid bone loss that begins in the cervical area because of the loss of cell adhesion [11]. Indirectly, IL-1Ra might play an important role in attachment of the junctional epithelium to the enamel surface by inhibiting laminin-5 degradation.

In conclusion, IL-1Ra has an important protective role in periodontal tissue by inhibition of MMP-13 functions. Similar to the use of anakinra, a therapeutic approach for periodontal disease using IL-1Ra might be useful and further investigation is needed for this potentially therapeutic molecule.
