# Correction: Interleukin-1 Receptor Antagonist Has a Novel Function in the Regulation of Matrix Metalloproteinase-13 Expression

**DOI:** 10.1371/journal.pone.0231910

**Published:** 2020-04-10

**Authors:** Hisashi Goto, Yuichi Ishihara, Takeshi Kikuchi, Ario Izawa, Nobuaki Ozeki, Eijiro Okabe, Yosuke Kamiya, Yusuke Ozawa, Hiroki Mizutani, Genta Yamamoto, Makio Mogi, Kazuhiko Nakata, Hatsuhiko Maeda, Toshihide Noguchi, Akio Mitani

The underlying data were not included with this article [1], although the Data Availability Statement in this article says, “All relevant data are within the paper.” The data are provided here in S1 File. Questions were raised as to whether the β-actin images provided corresponded to the published panels in Figs 2, 3, and 4; the authors commented that the images were compressed vertically during figure preparation and that these are indeed the correct supporting image data.

β-actin control data for all western blot experiments were obtained using parallel blots prepared using the same protein samples as the corresponding experimental blots. The same β-actin blot was provided for Figs 2 and 3. The authors confirm that these experiments used the same protein extracts and blot, and so the same control blot applied to both experiments (IL-1Ra in Fig 2B and MMP-13 in Fig 3B). Similarly, in Fig 4B, the same protein samples were used in the TIMP-1 and TIMP-2 experiments such that the same β-actin data applied to both experimental blots.

For several of the western blots, the background area in the image data provided appears uniform. The authors clarified that they utilized LAS-3000 (Fujifilm, Tokyo, Japan) for digitizing the density of immune complexes and at that time, adjusted the capture time and setting (brightness and contrast). The images included in S1 File are these brightness/contrast adjusted digital images of the original film.

For the IL-1Ra blot in Figure 2 and the TIMP-1 blot in Fig 4B, the original blot images (S1 File) include numerous bands aside from those shown in the figures. The authors estimated the targets from the size of the molecular weight with reference to the data sheet of antibodies. No experiments were performed to confirm the specificity of the antibodies used in these panels. Readers are advised to interpret the results of these experiments accordingly.

In reviewing the original data, it came to light that the lanes in TIMP-1 and TIMP-2 blots in Fig 4B were mislabeled. A corrected figure is provided here. Also, in light of the primary data for the TIMP-1 and TIMP-2 western blot experiments in Fig 4B, the last sentence in the section “TIMP expression in IL-1Ra siRNA-transfected cells” is not supported and is updated to: “Western blot results indicated that TIMP-1 (23 kDa) and TIMP-2 (21 kDa) proteins are expressed at slightly higher levels in IL-1Ra siRNA samples than in controls (Fig 4B and S1 File).”

**Fig 4:**
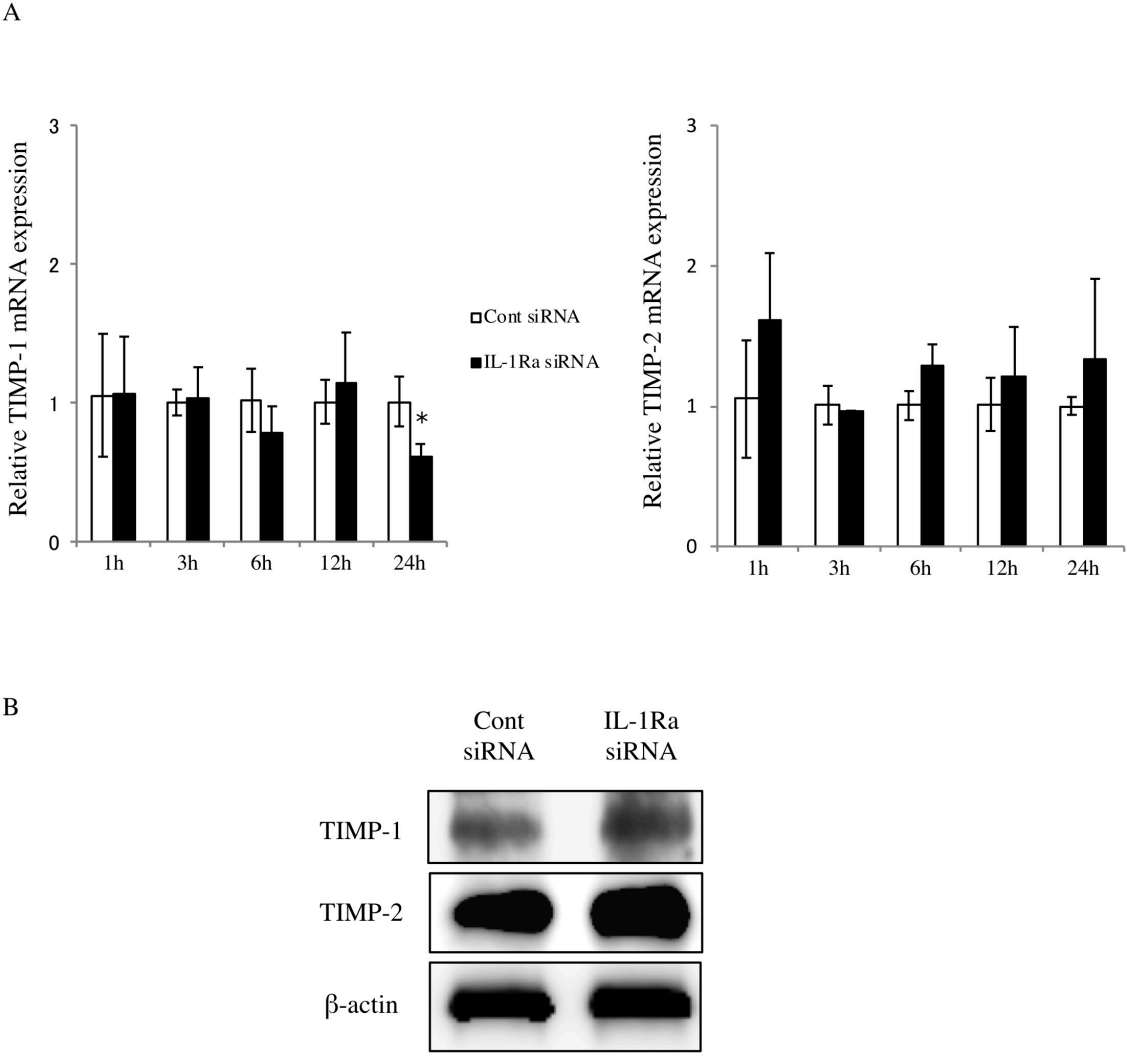
TIMP expression in IL-1Ra siRNA-transfected cells. (A) TIMP-1 and TIMP-2 mRNA expression in IL-1Ra and control siRNA-transfected cells. The cells were cultured for 1, 3, 6, 12, and 24 hours, and then mRNA levels were determined using real-time PCR. Values represent fold changes. Differences among groups were analyzed using the Student’s *t*-test. Data are expressed as the mean ± SD (n = 3). *p < 0.05 vs each time point control. (B) Western blot of TIMP-1 (23 kDa) and -2 (21 kDa) in cells transfected with either IL-1Ra or control siRNAs. Data are representative of three independent experiments.

## Supporting information

S1 File Original data underlying Figure 1. (ZIP)

S2 File Original data underlying Figure 2. (ZIP)

S3 File Original data underlying Figure 3. (ZIP)

S4 File Original data underlying Fig 4. (ZIP)

S5 File Original data underlying Figure 5. (ZIP)

S6 File Original data underlying Figure 6. (ZIP)

S7 File Original data underlying Figure 7. (ZIP)

S8 File Original data underlying Figure 8. (ZIP)
