# Supplementary figures and images for: Correction: Interleukin-1 Receptor Antagonist Has a Novel Function in the Regulation of Matrix Metalloproteinase-13 Expression
Source: PLoS One. 2020 Apr 10;15(4):e0231910. doi: 10.1371/journal.pone.0231910 (PMC7147734; doi:10.1371/journal.pone.0231910)

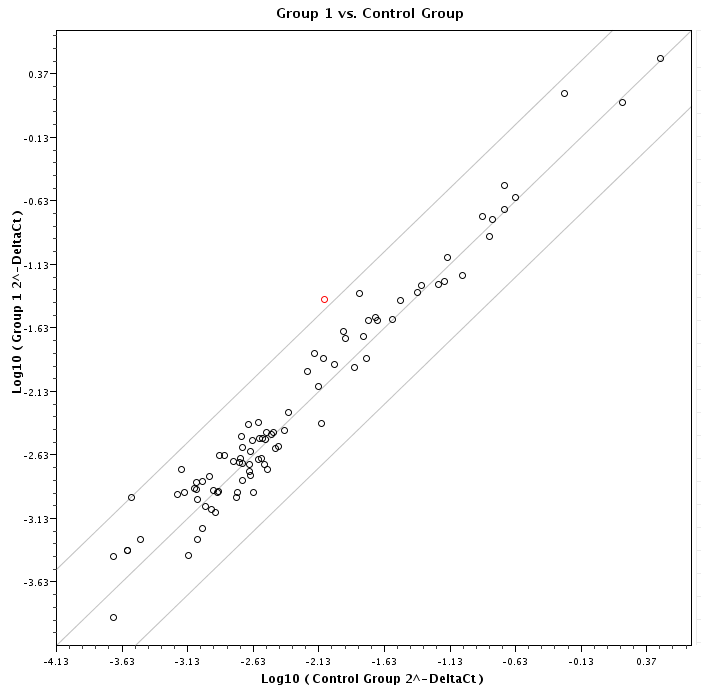

Supplement: S1 File — (ZIP) [file pone.0231910.s001.zip › S1_File/Fig1.screen shot.png]

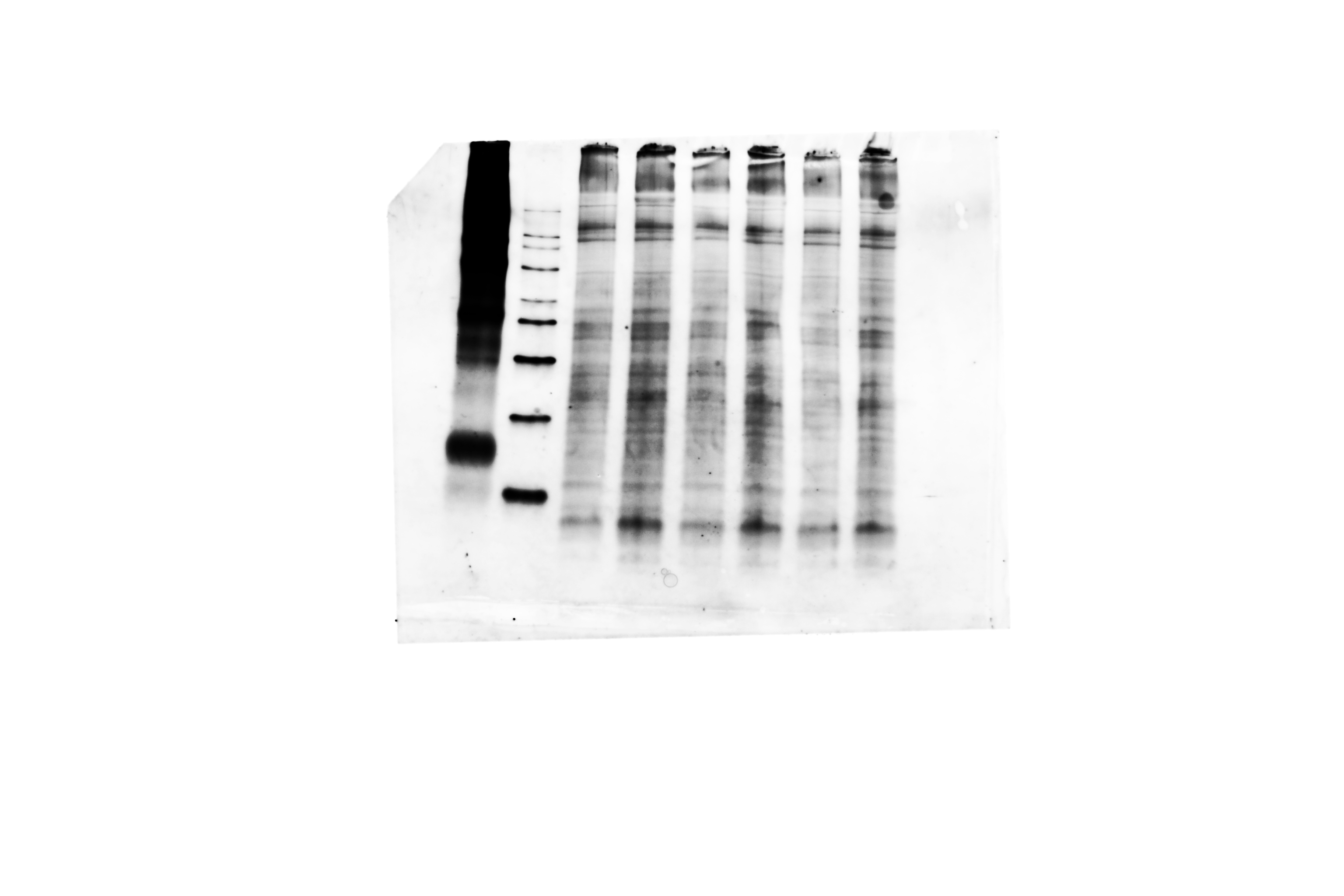

Supplement: S2 File — (ZIP) [file pone.0231910.s002.zip › S2_File/Fig2.-5.tif]

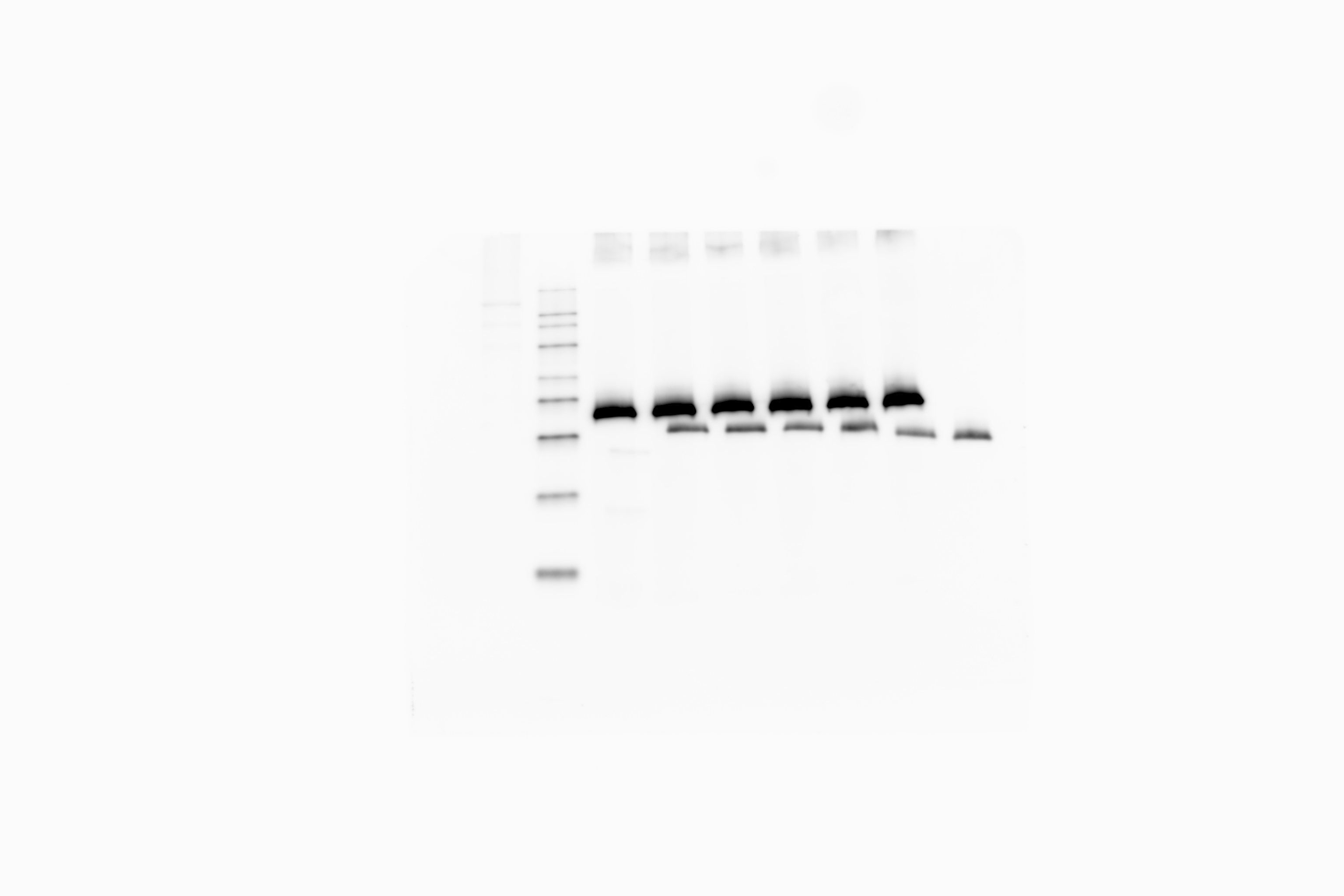

Supplement: S2 File — (ZIP) [file pone.0231910.s002.zip › S2_File/Fig2.actin.tif]

## Slide 1
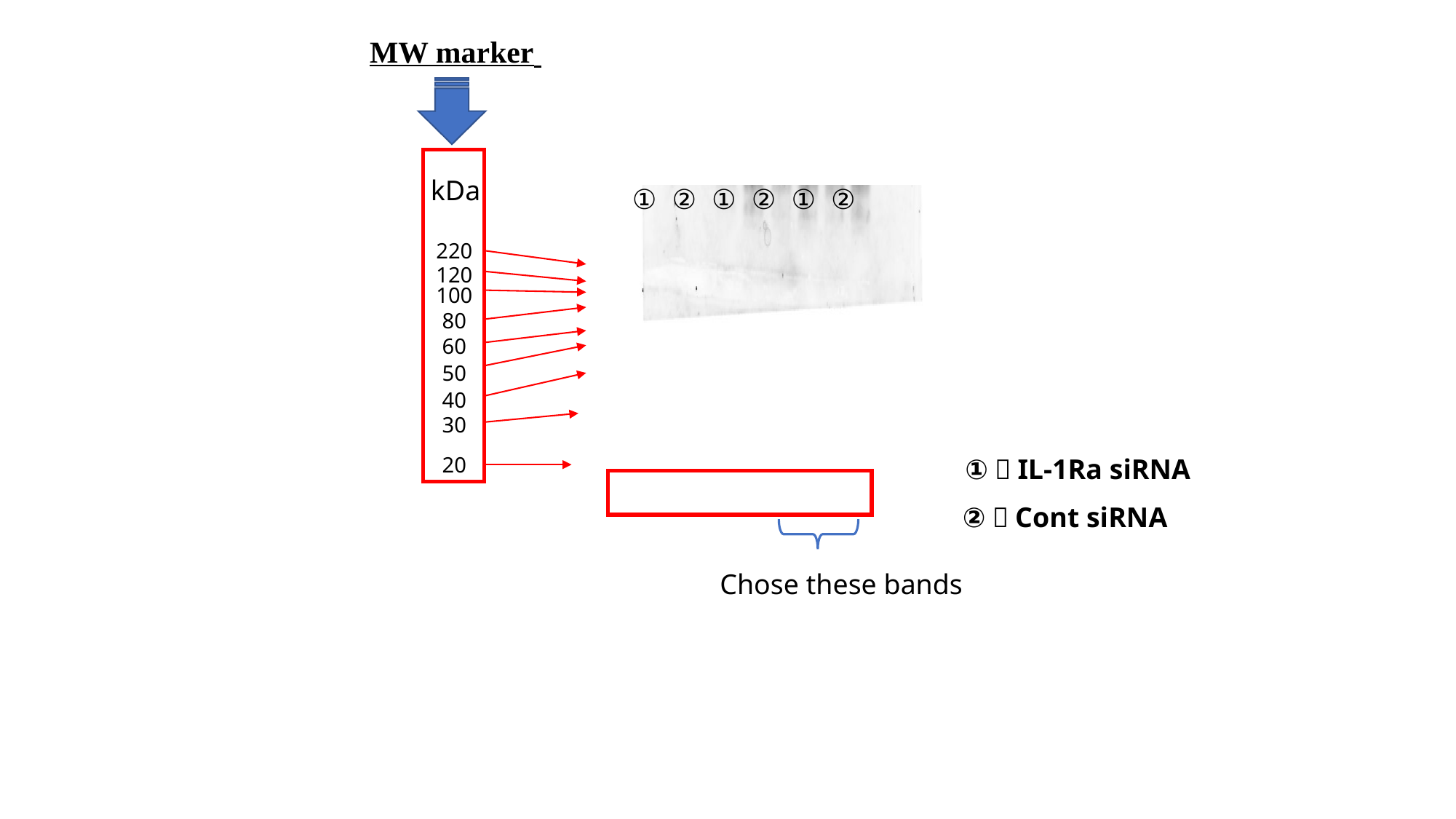

MW marker
kDa
①
②
①
②
①
②
220
120
100
80
60
50
40
30
20
①；IL-1Ra siRNA
②；Cont siRNA
Chose these bands

Supplement: S2 File — (ZIP) [file pone.0231910.s002.zip › S2_File/Fig2.WBIL-1Ra.pptx]

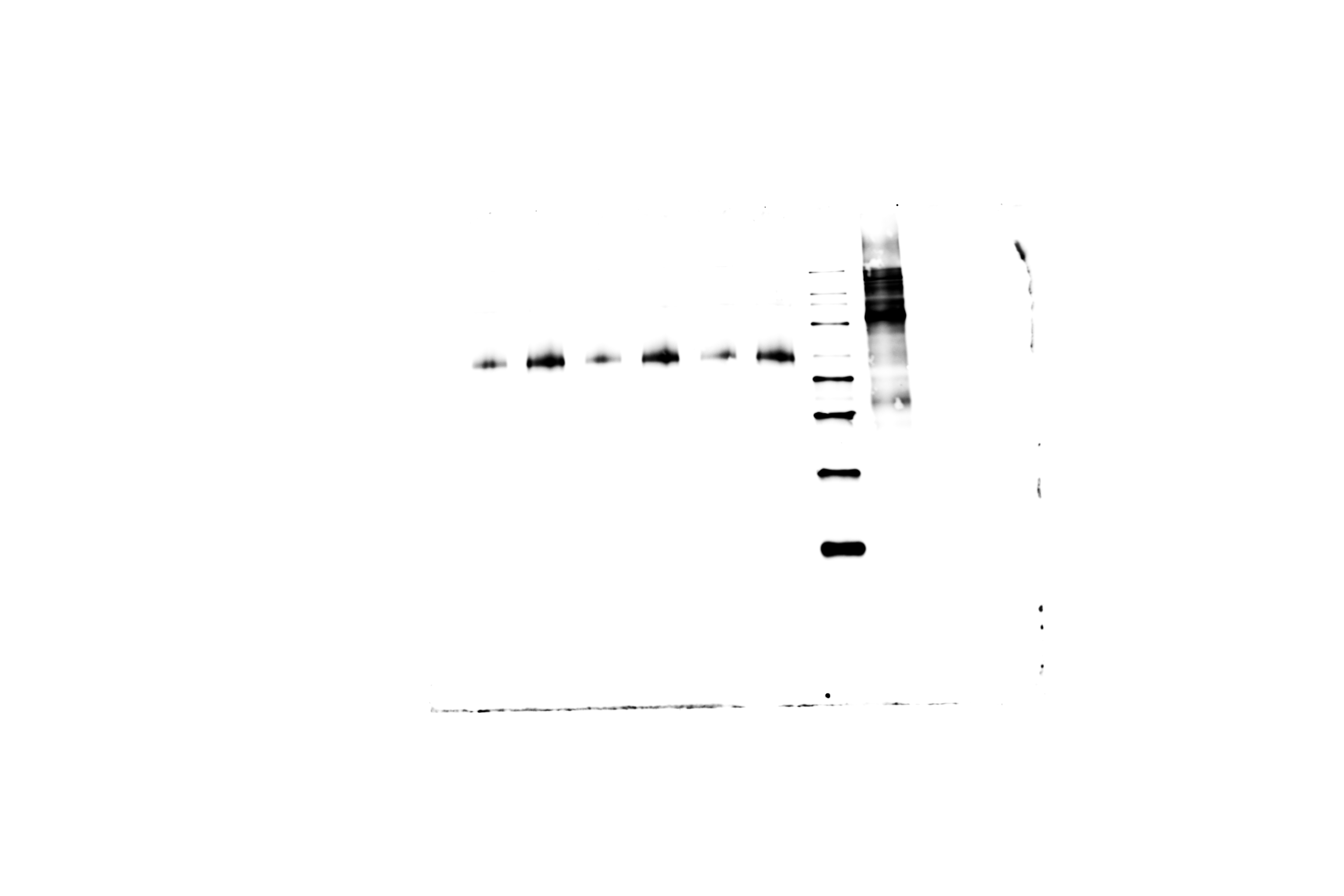

Supplement: S3 File — (ZIP) [file pone.0231910.s003.zip › S3_File/B/Fig3B.120s.tif]

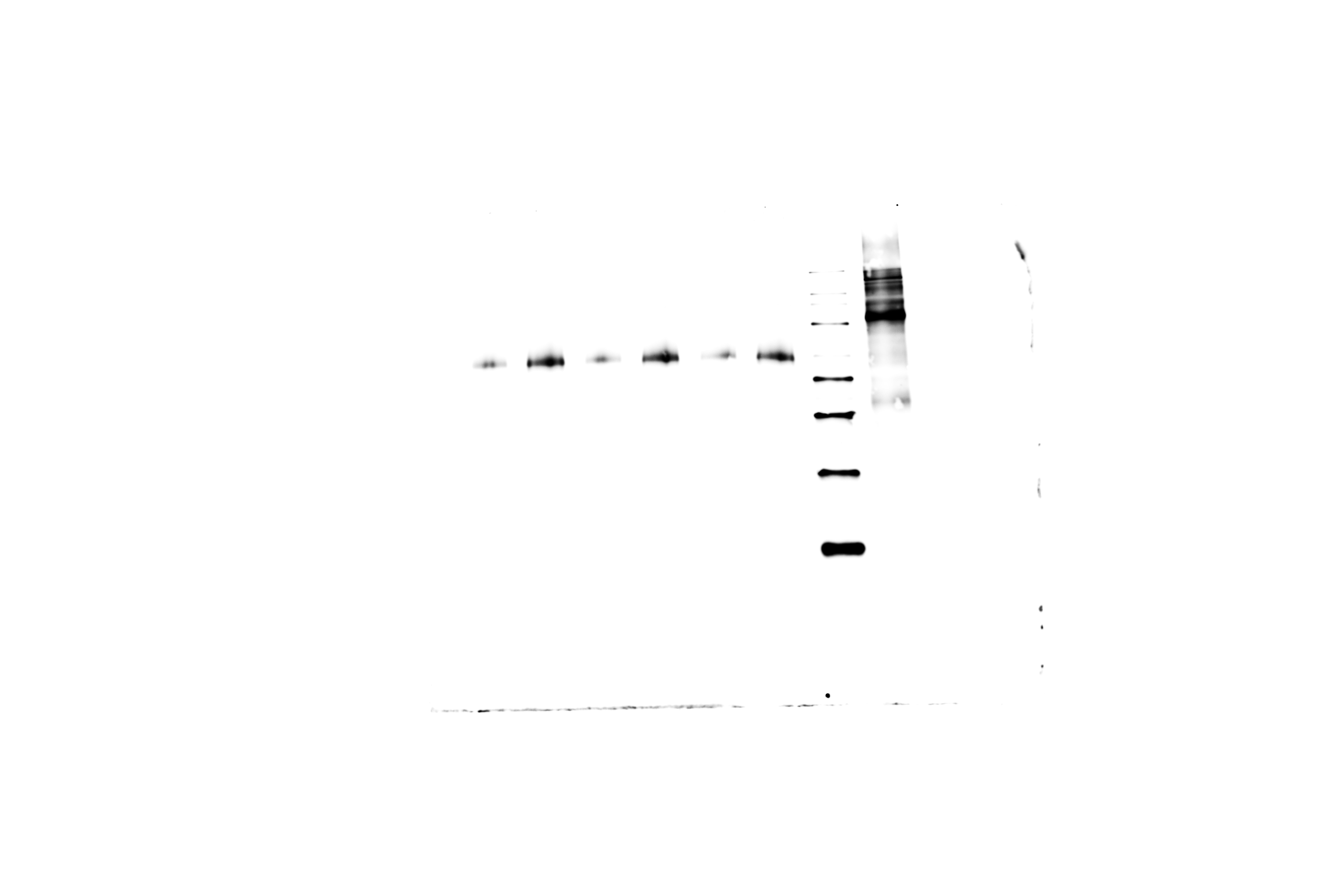

Supplement: S3 File — (ZIP) [file pone.0231910.s003.zip › S3_File/B/WBexposure(each 10sec)/Fig3B.100s.tif]

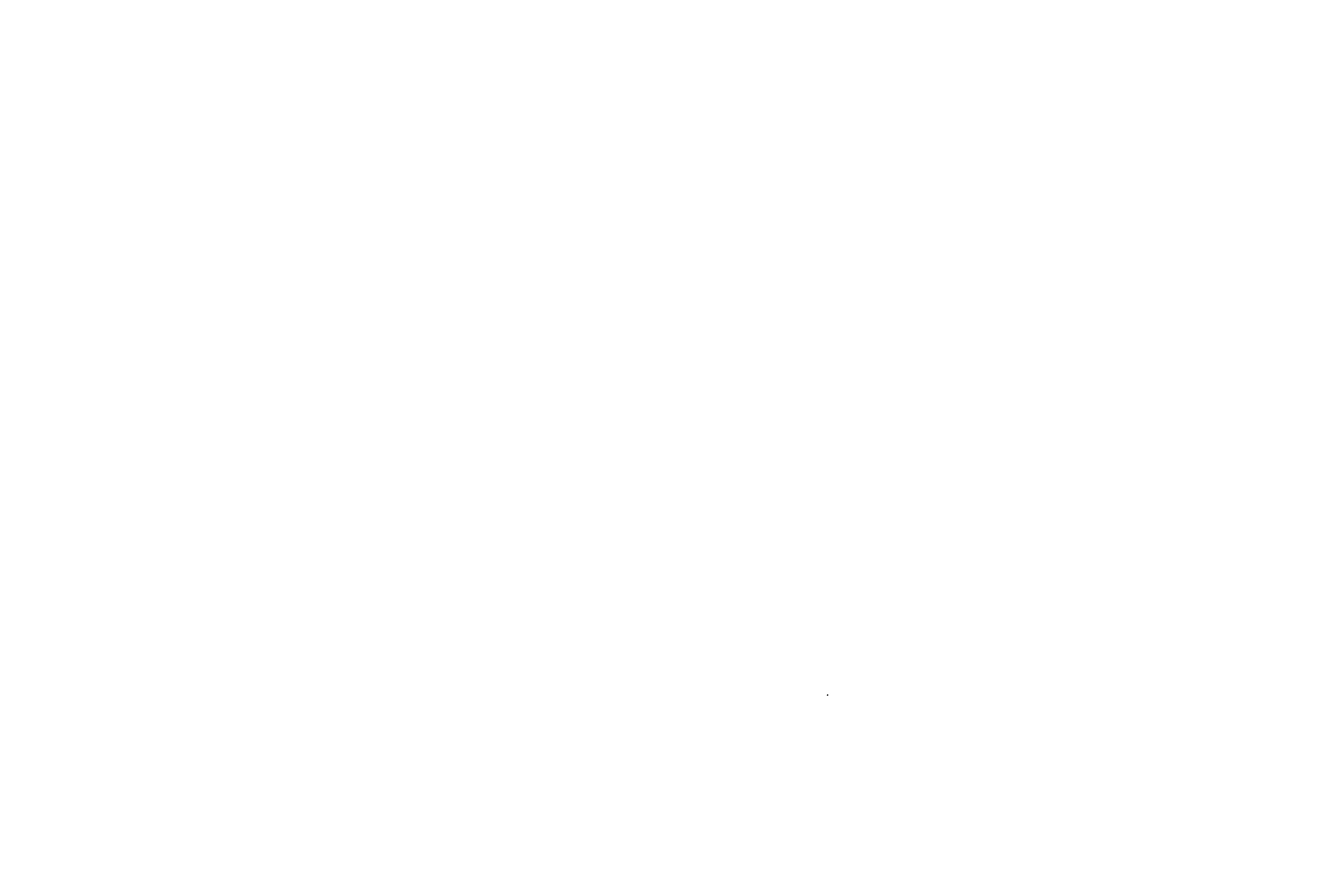

Supplement: S3 File — (ZIP) [file pone.0231910.s003.zip › S3_File/B/WBexposure(each 10sec)/Fig3B.10s.tif]

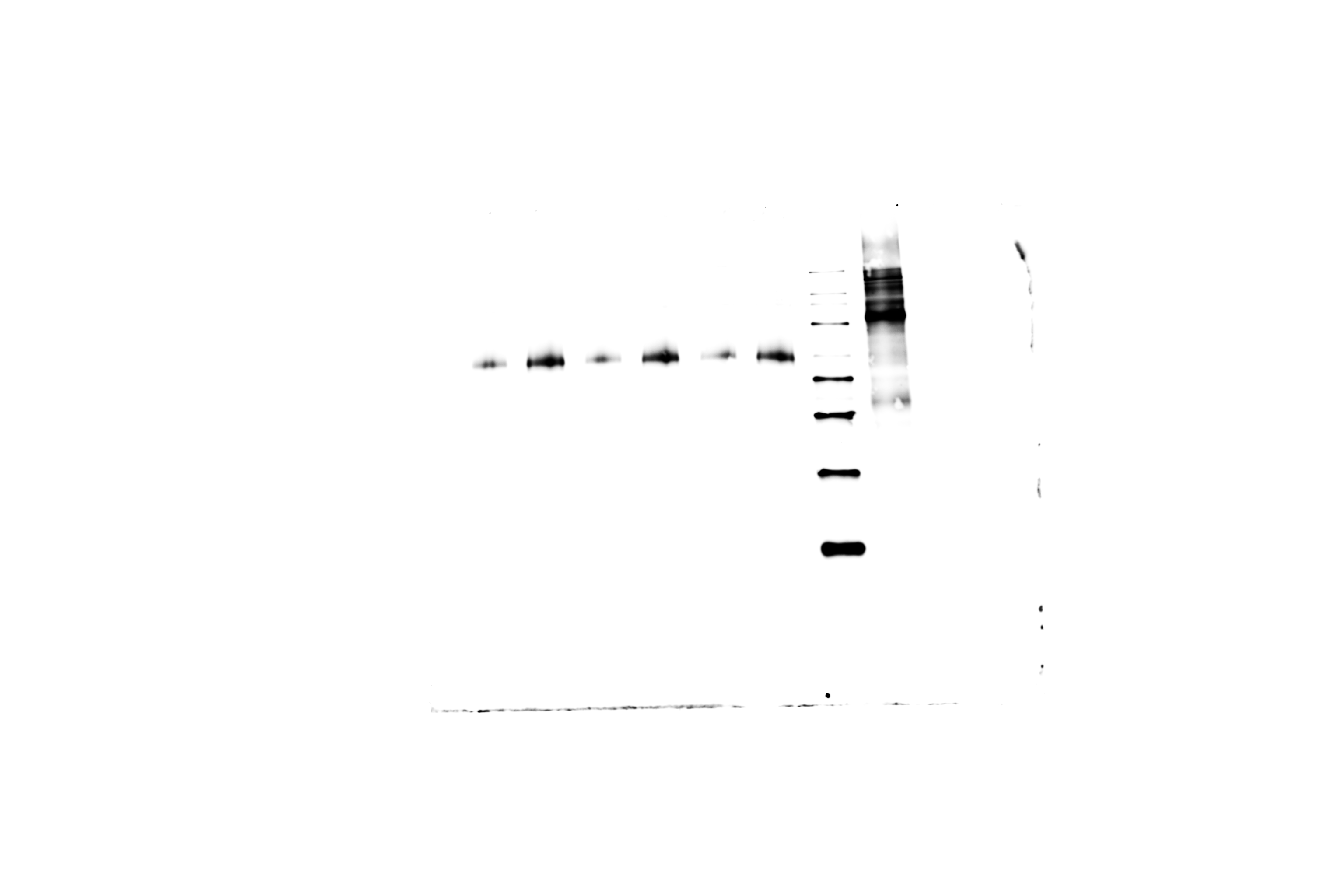

Supplement: S3 File — (ZIP) [file pone.0231910.s003.zip › S3_File/B/WBexposure(each 10sec)/Fig3B.110s.tif]

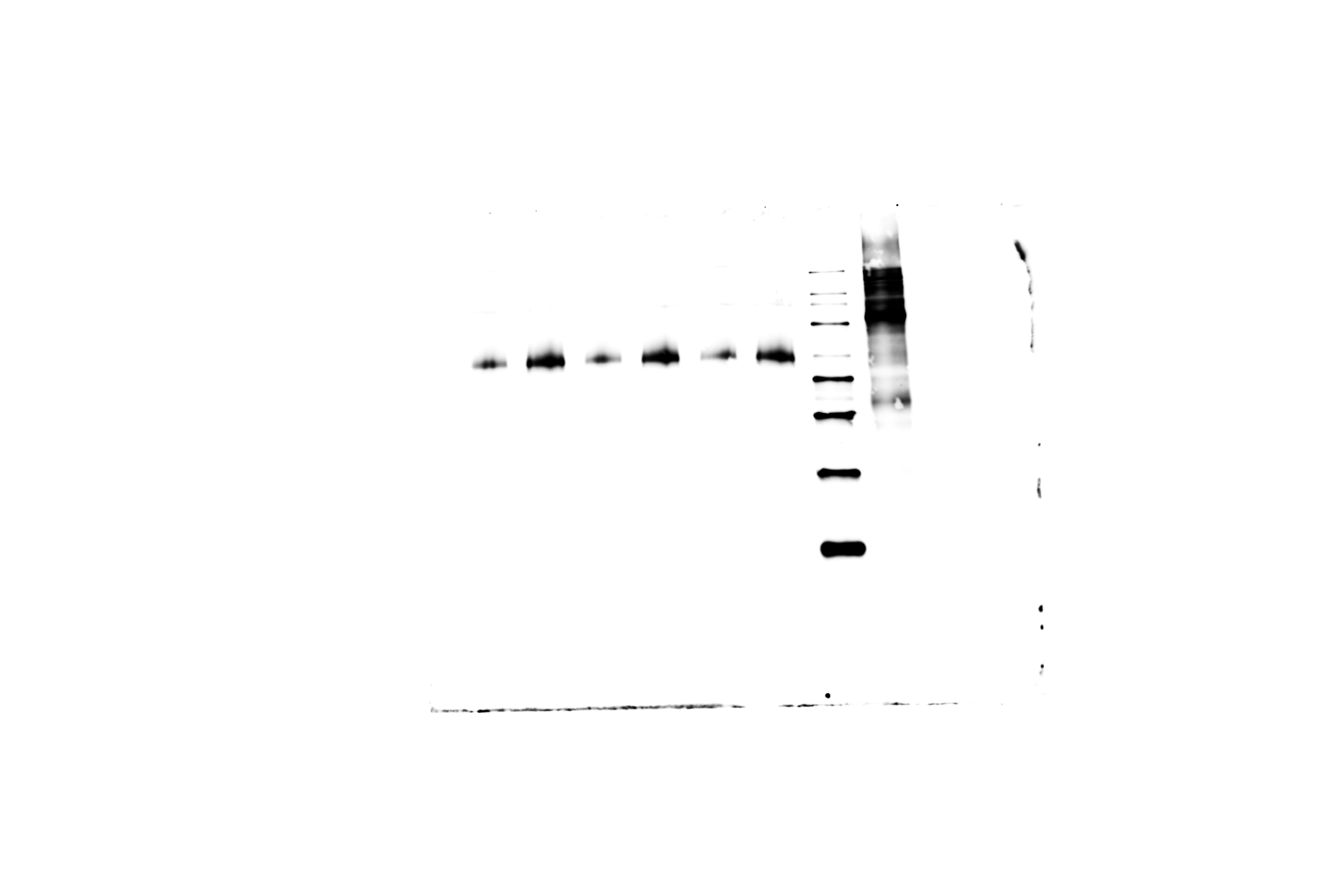

Supplement: S3 File — (ZIP) [file pone.0231910.s003.zip › S3_File/B/WBexposure(each 10sec)/Fig3B.130s.tif]

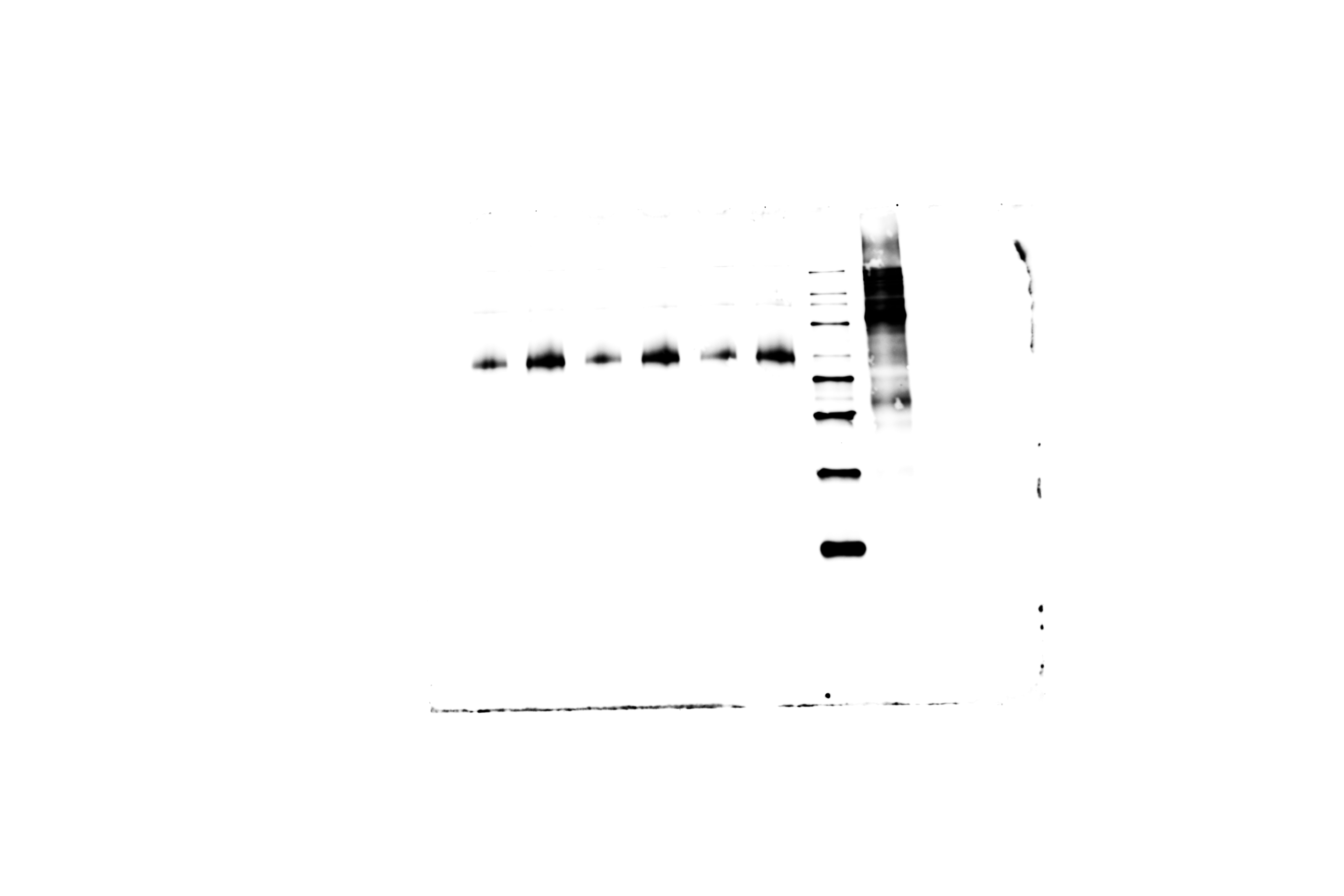

Supplement: S3 File — (ZIP) [file pone.0231910.s003.zip › S3_File/B/WBexposure(each 10sec)/Fig3B.140s.tif]

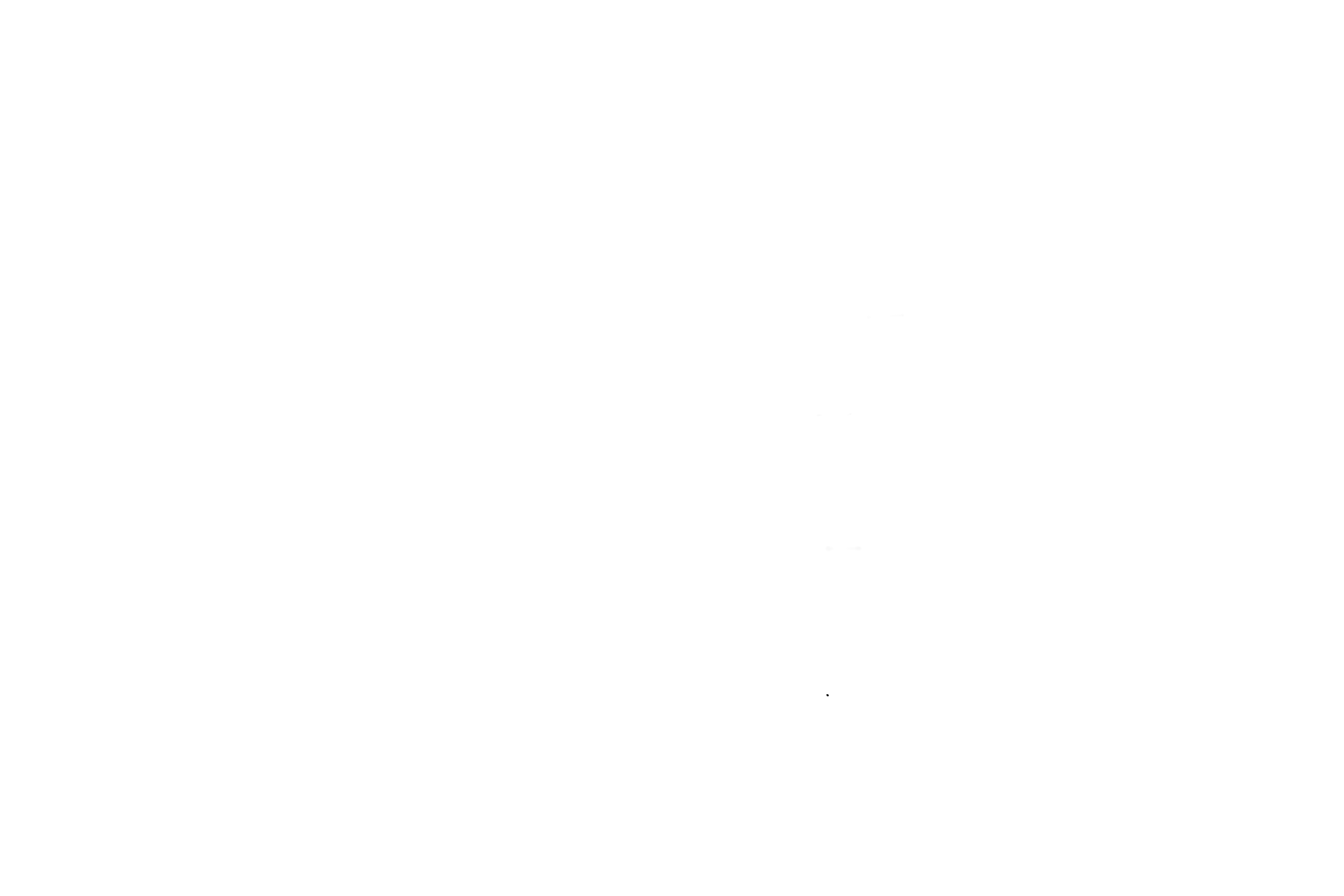

Supplement: S3 File — (ZIP) [file pone.0231910.s003.zip › S3_File/B/WBexposure(each 10sec)/Fig3B.20s.tif]

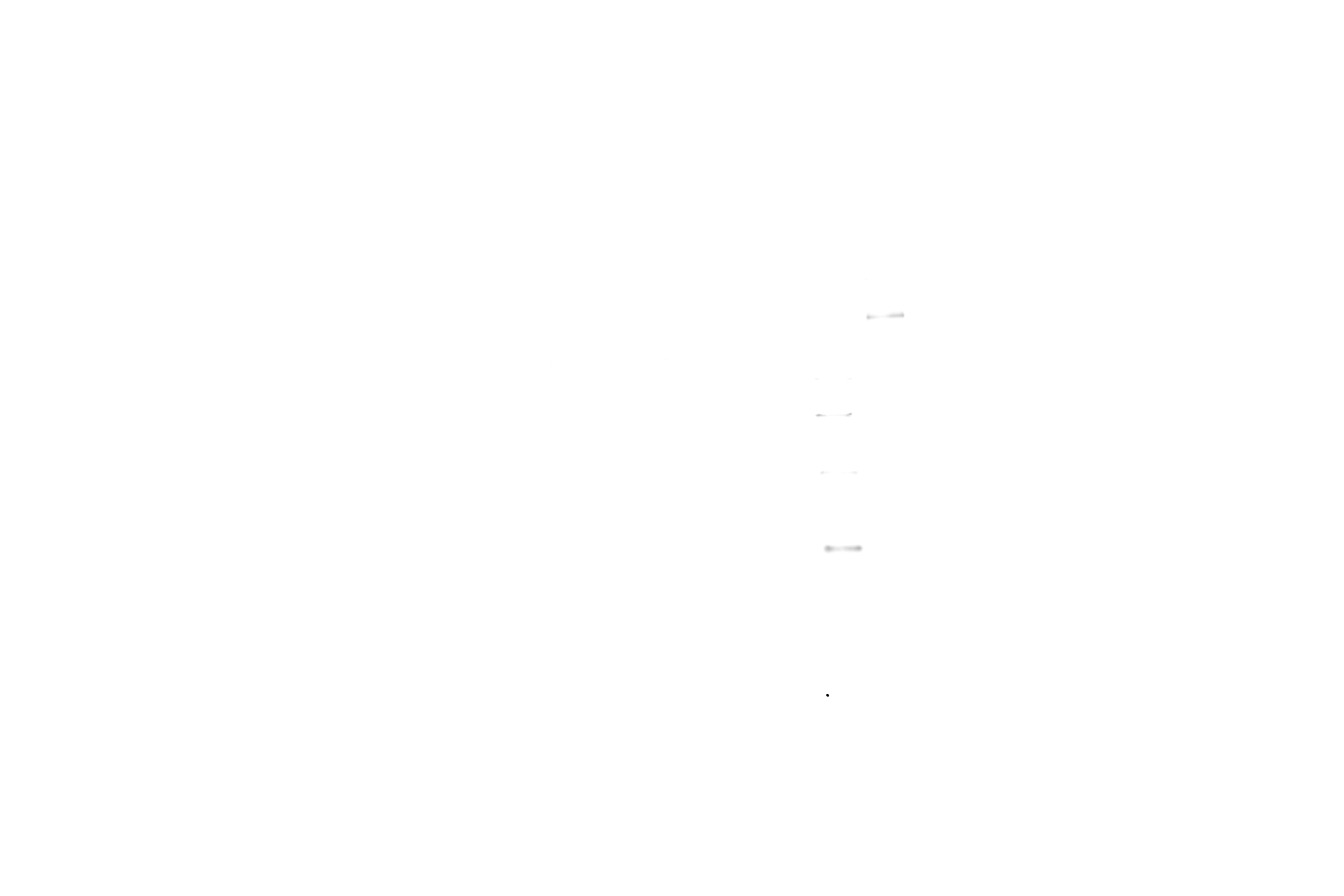

Supplement: S3 File — (ZIP) [file pone.0231910.s003.zip › S3_File/B/WBexposure(each 10sec)/Fig3B.30s.tif]

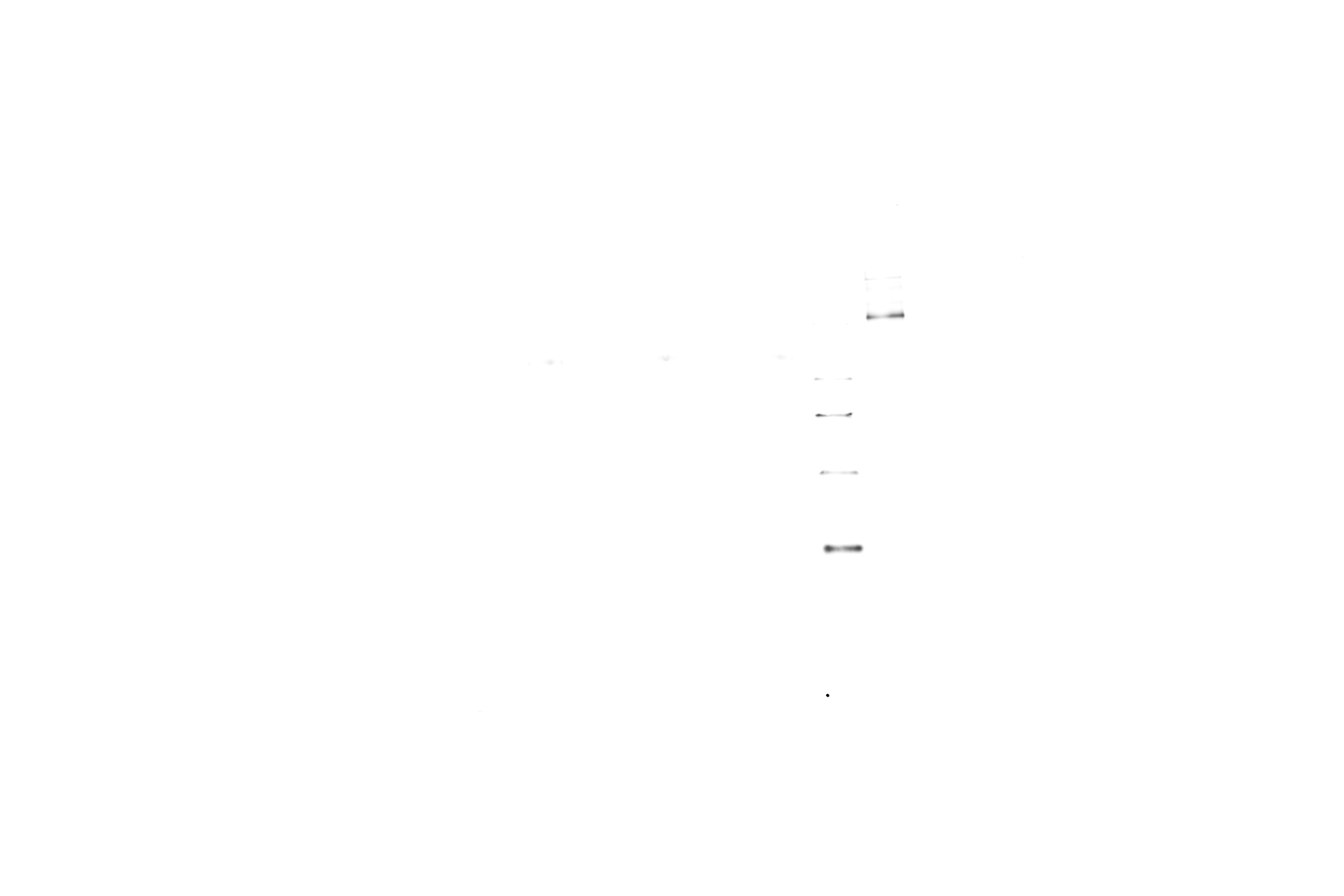

Supplement: S3 File — (ZIP) [file pone.0231910.s003.zip › S3_File/B/WBexposure(each 10sec)/Fig3B.40s.tif]

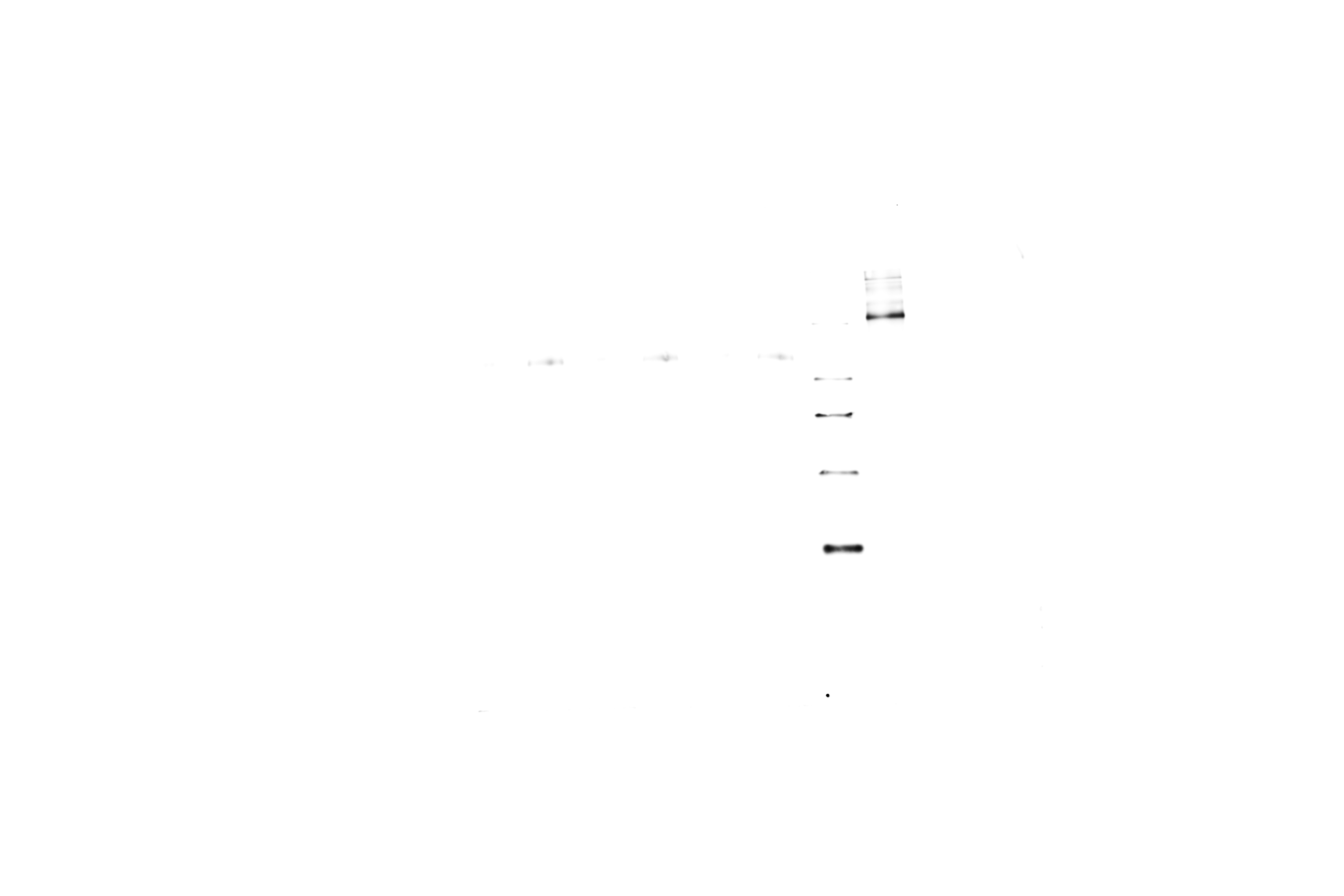

Supplement: S3 File — (ZIP) [file pone.0231910.s003.zip › S3_File/B/WBexposure(each 10sec)/Fig3B.50s.tif]

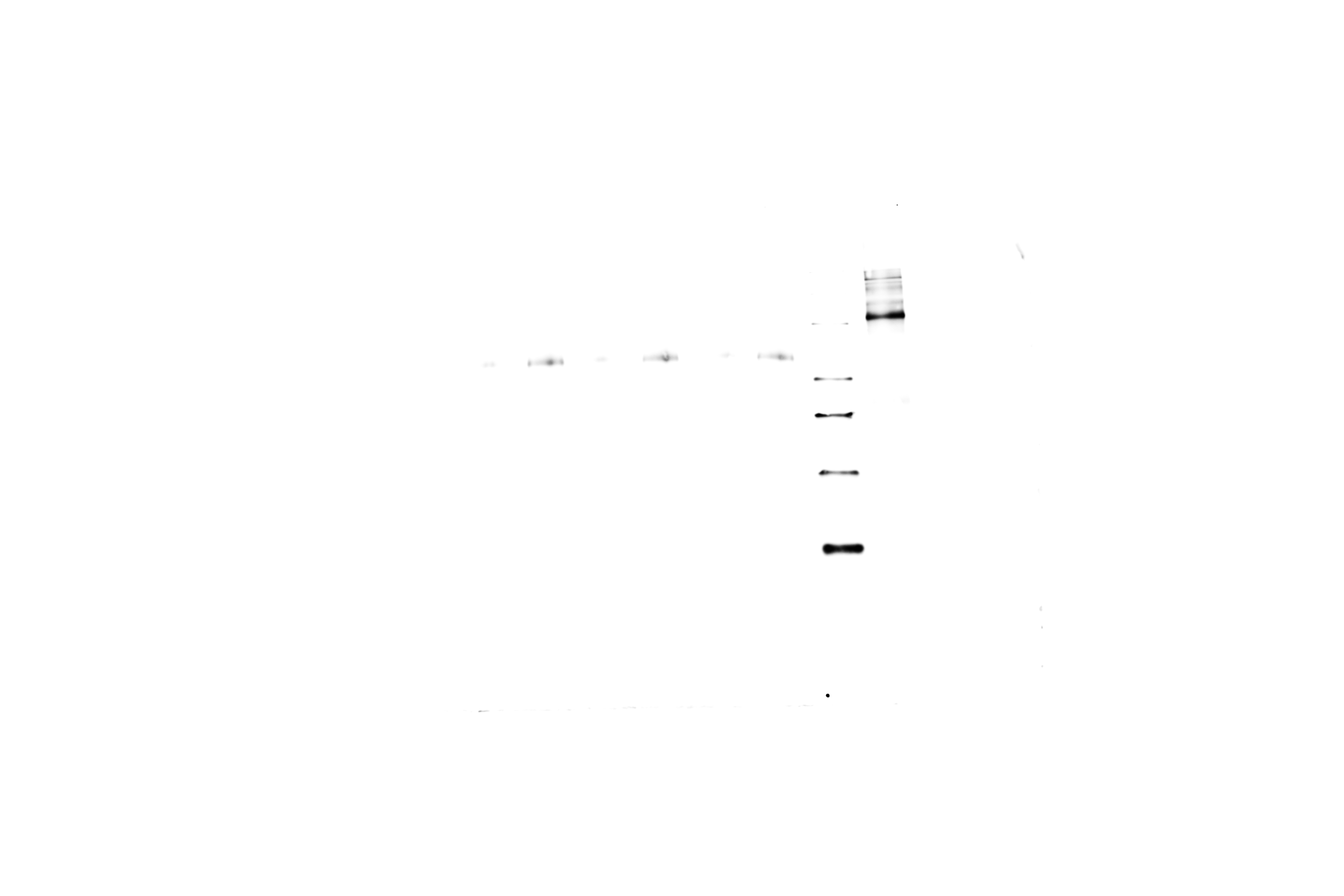

Supplement: S3 File — (ZIP) [file pone.0231910.s003.zip › S3_File/B/WBexposure(each 10sec)/Fig3B.60s.tif]

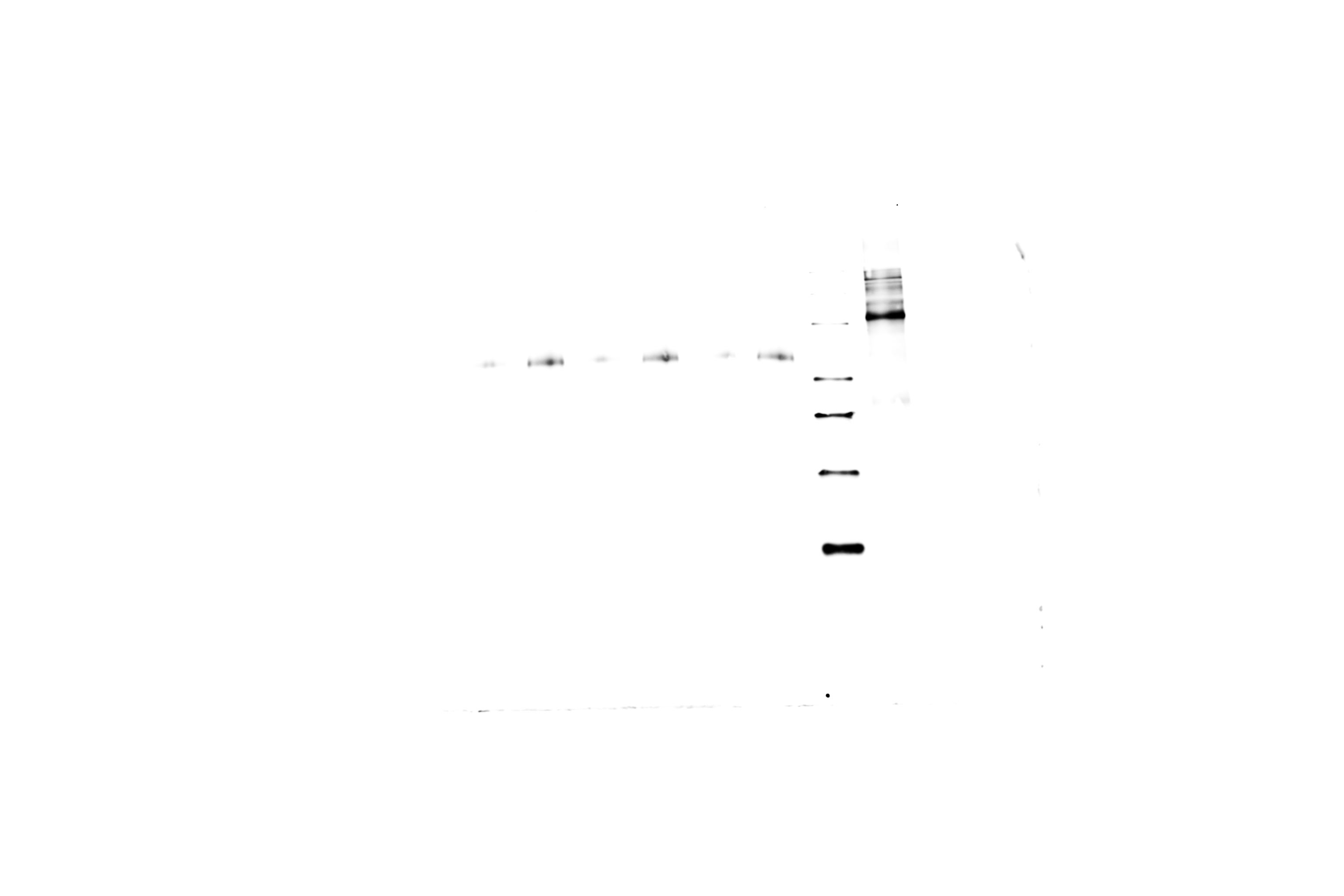

Supplement: S3 File — (ZIP) [file pone.0231910.s003.zip › S3_File/B/WBexposure(each 10sec)/Fig3B.70s.tif]

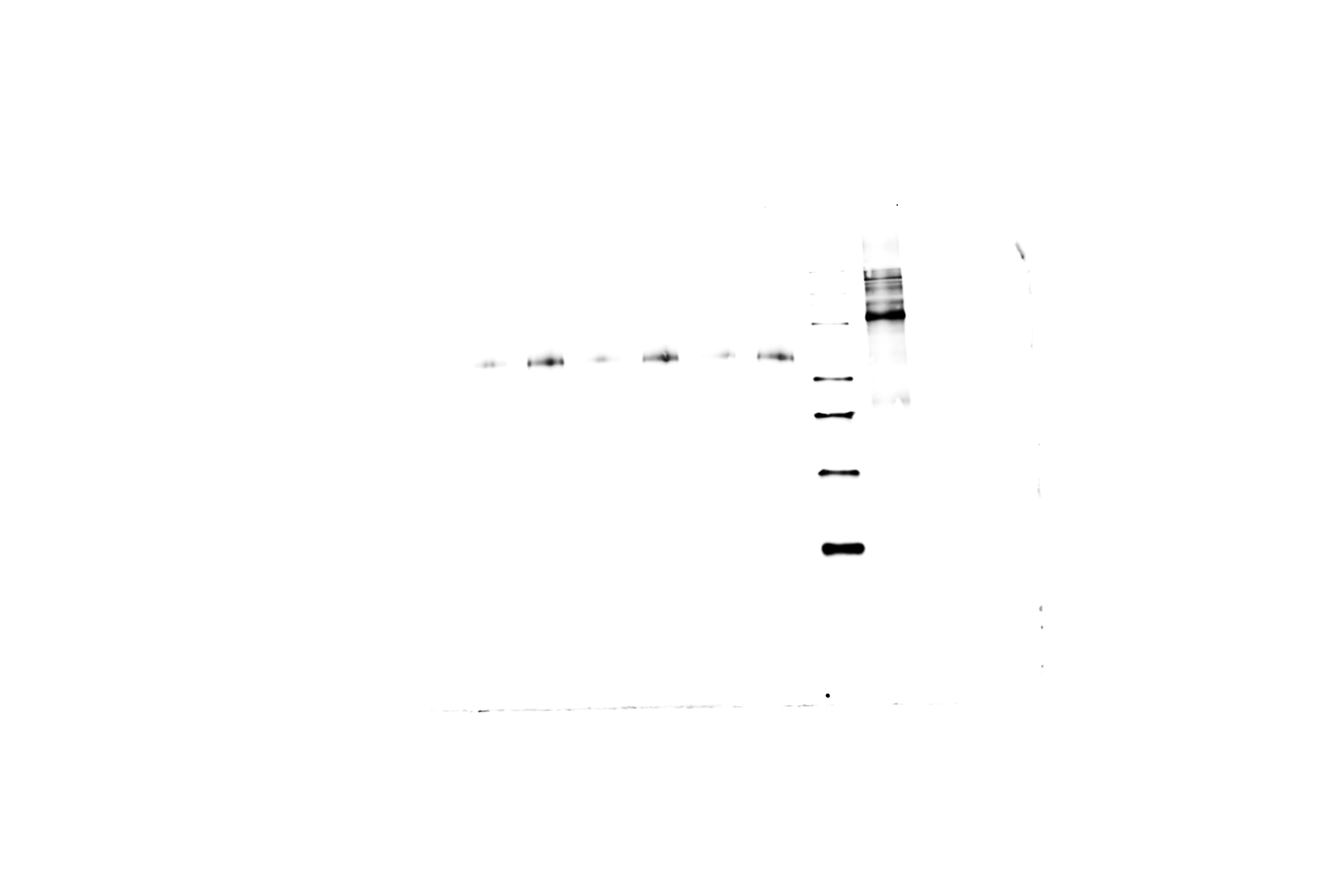

Supplement: S3 File — (ZIP) [file pone.0231910.s003.zip › S3_File/B/WBexposure(each 10sec)/Fig3B.80s.tif]

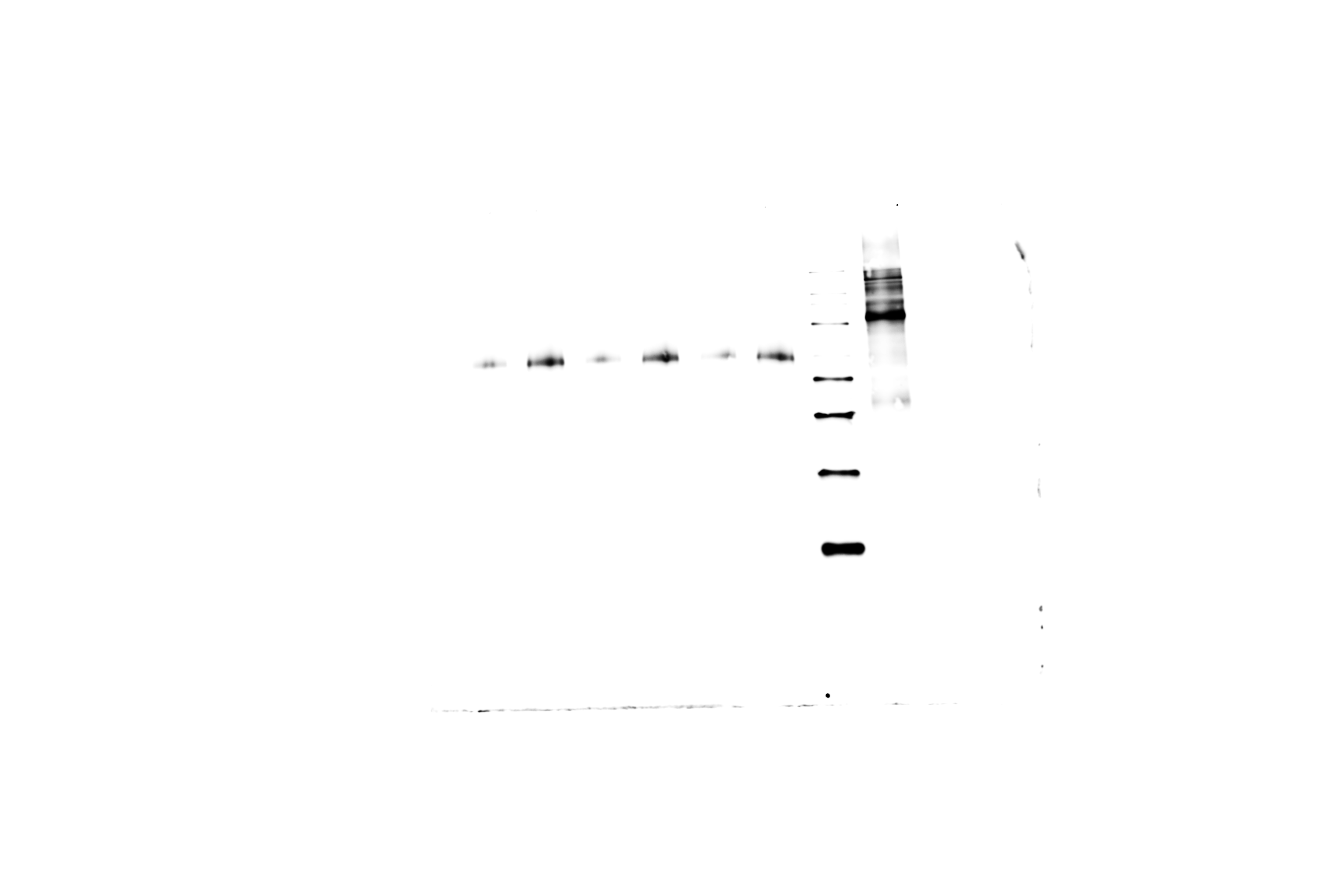

Supplement: S3 File — (ZIP) [file pone.0231910.s003.zip › S3_File/B/WBexposure(each 10sec)/Fig3B.90s.tif]

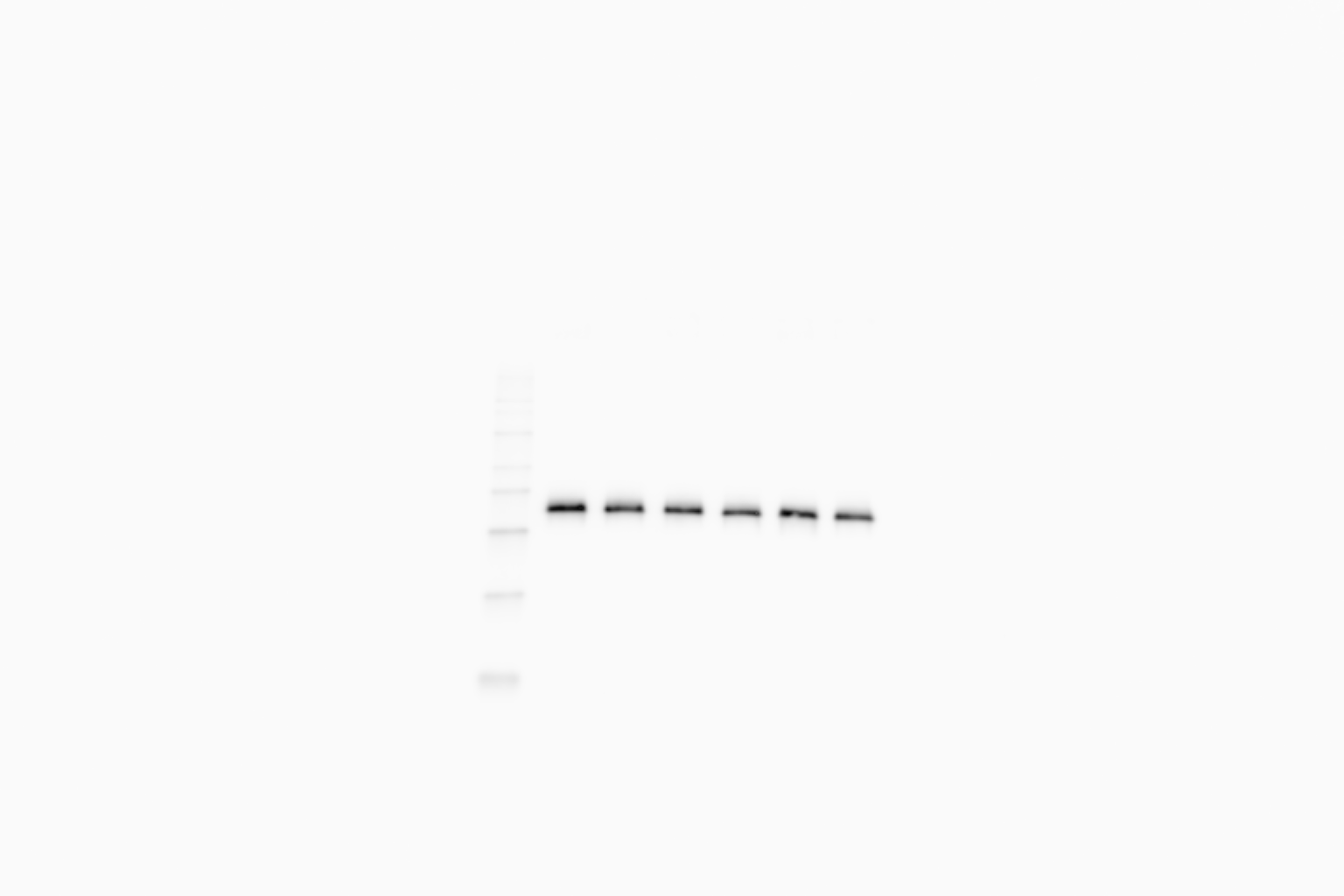

Supplement: S4 File — (ZIP) [file pone.0231910.s004.zip › S4_File/B/Fig4B.actin(each 10sec)/10s.tif]

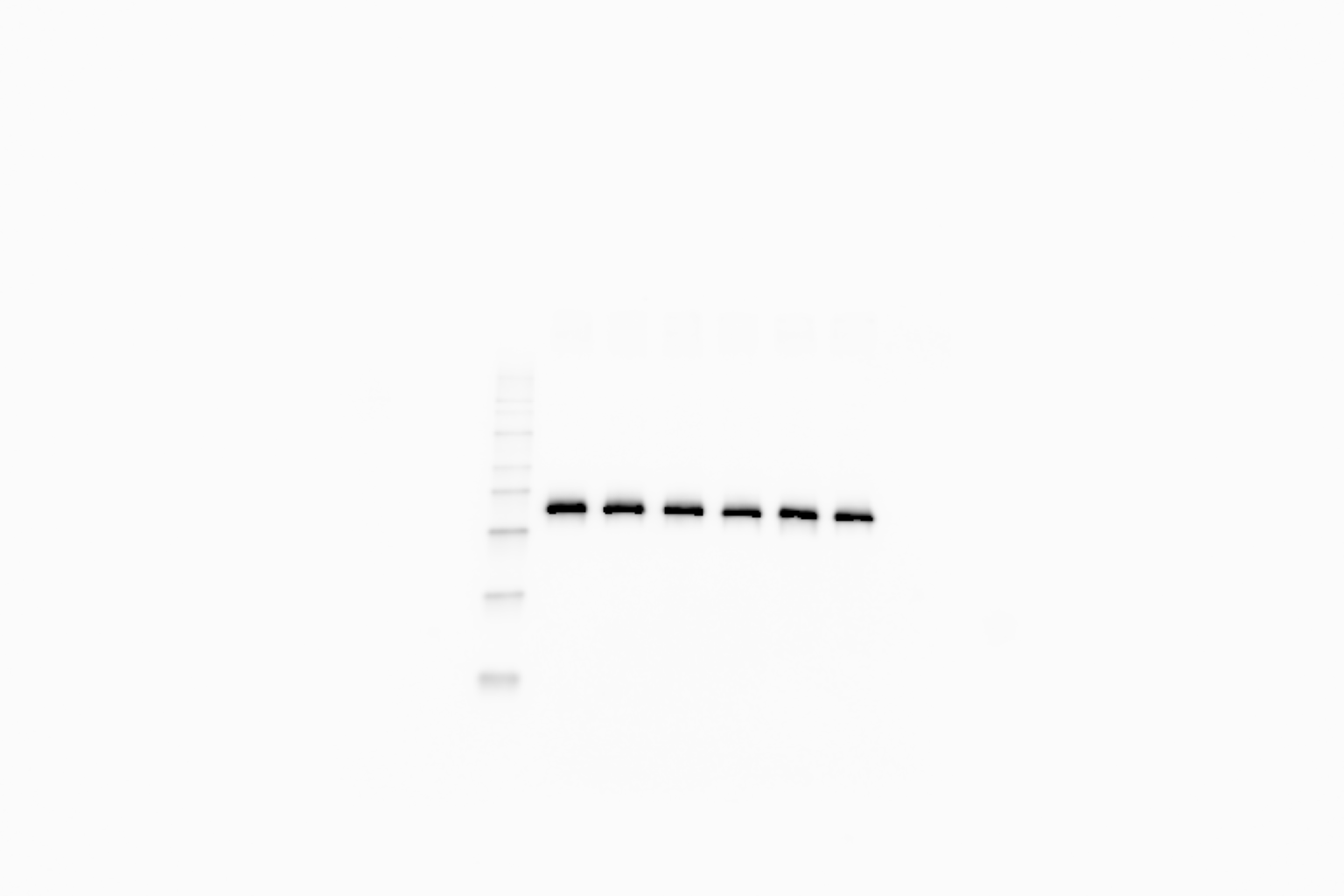

Supplement: S4 File — (ZIP) [file pone.0231910.s004.zip › S4_File/B/Fig4B.actin(each 10sec)/20s.tif]

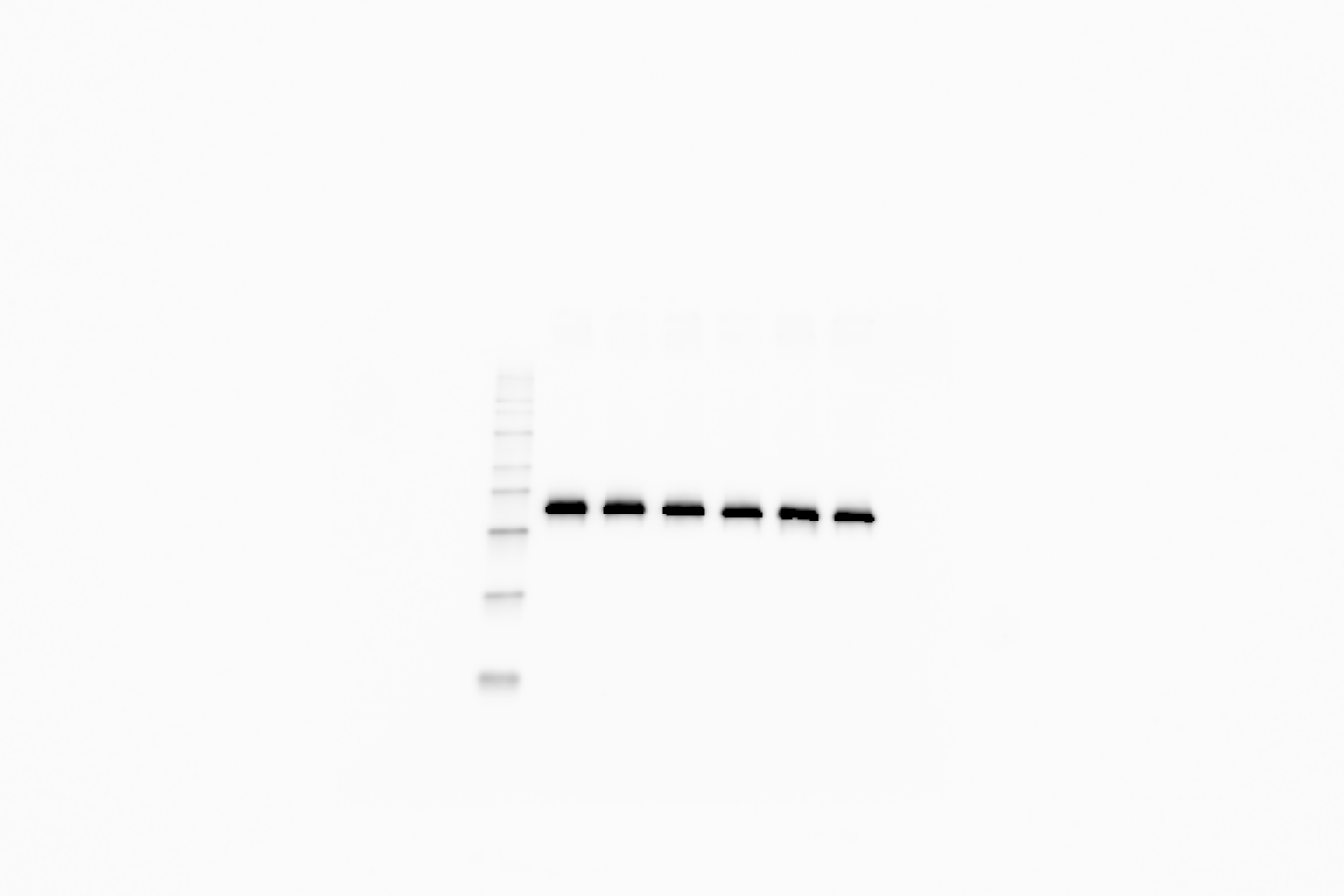

Supplement: S4 File — (ZIP) [file pone.0231910.s004.zip › S4_File/B/Fig4B.actin(each 10sec)/30s.tif]

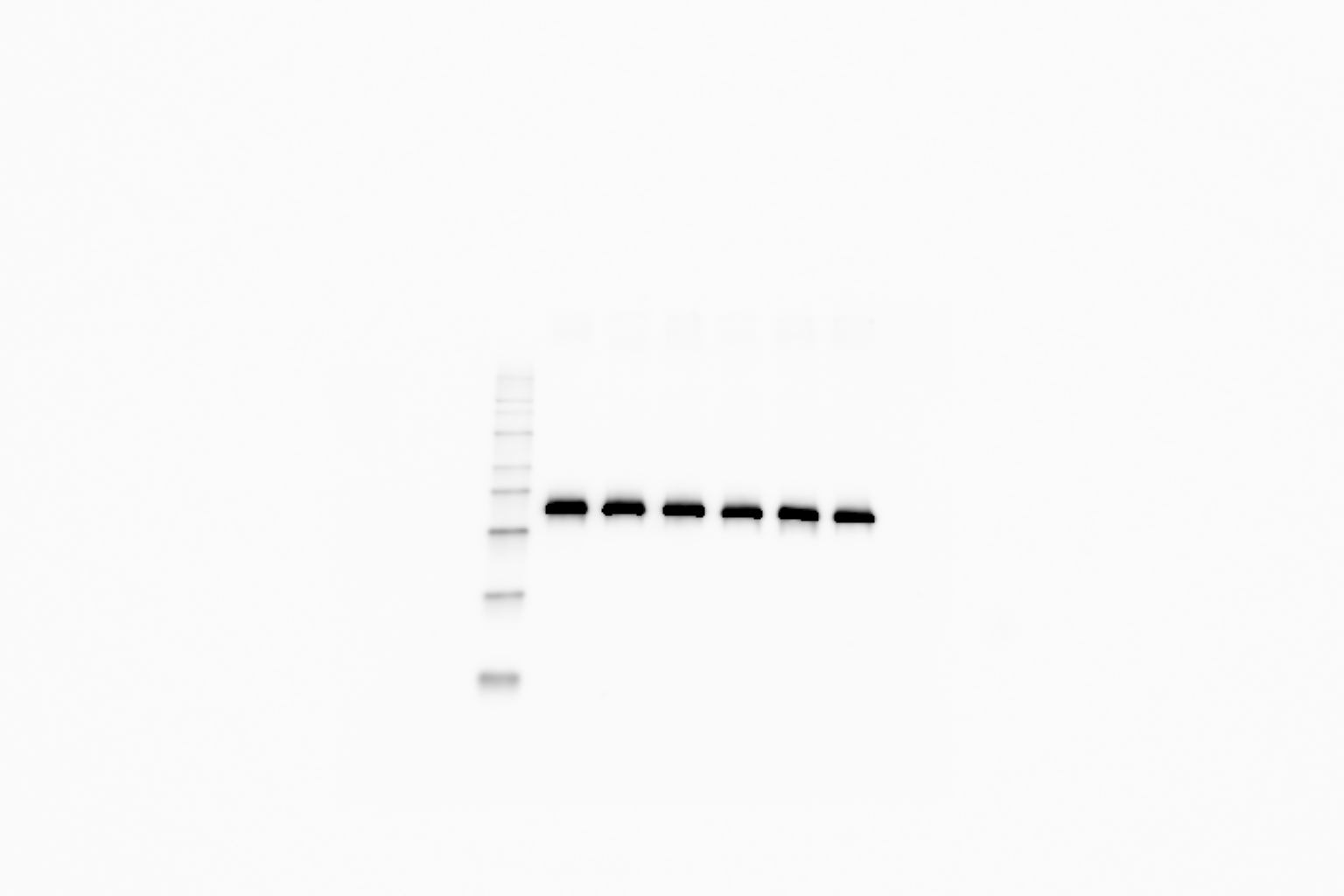

Supplement: S4 File — (ZIP) [file pone.0231910.s004.zip › S4_File/B/Fig4B.actin(each 10sec)/40s.tif]

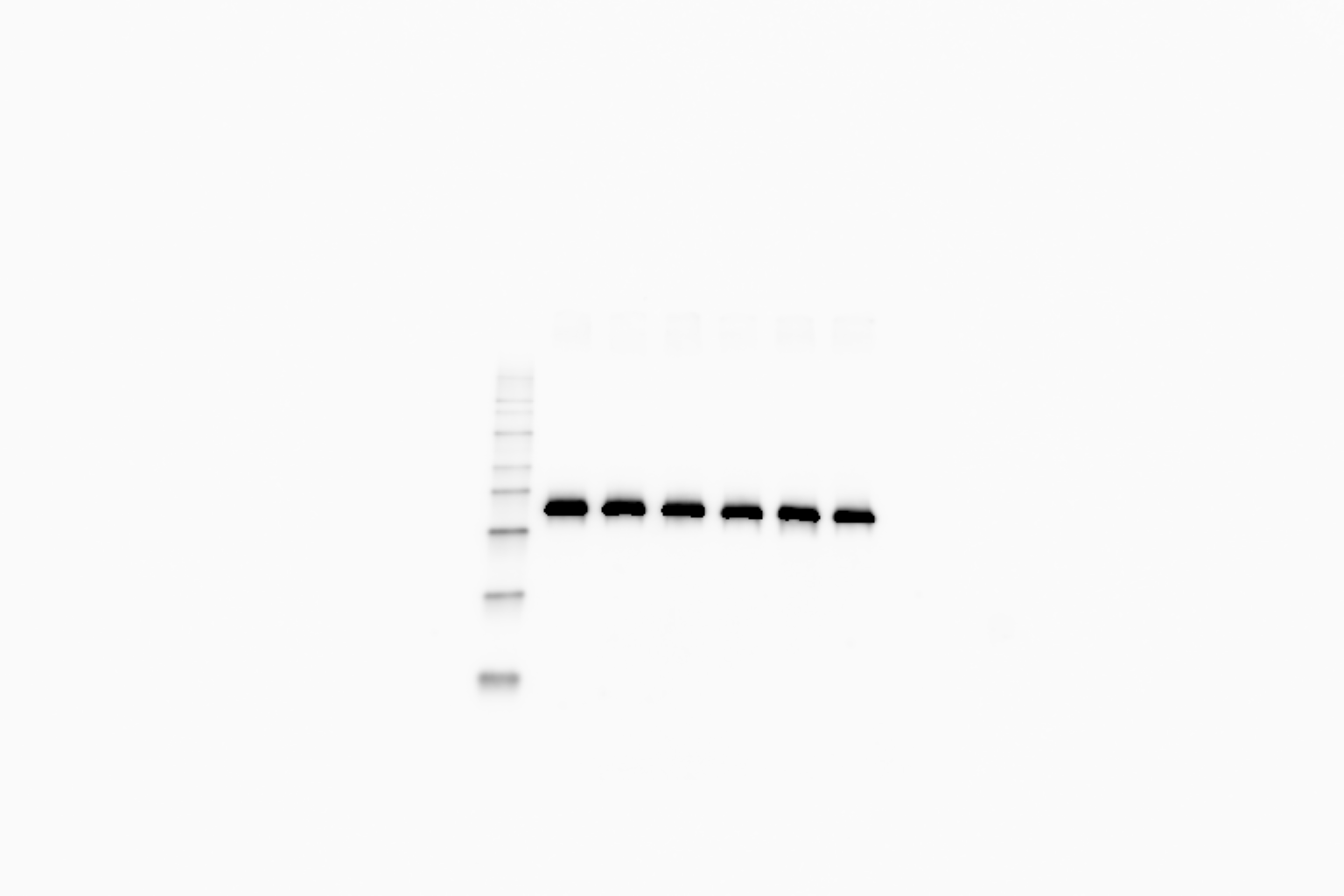

Supplement: S4 File — (ZIP) [file pone.0231910.s004.zip › S4_File/B/Fig4B.actin(each 10sec)/50s.tif]

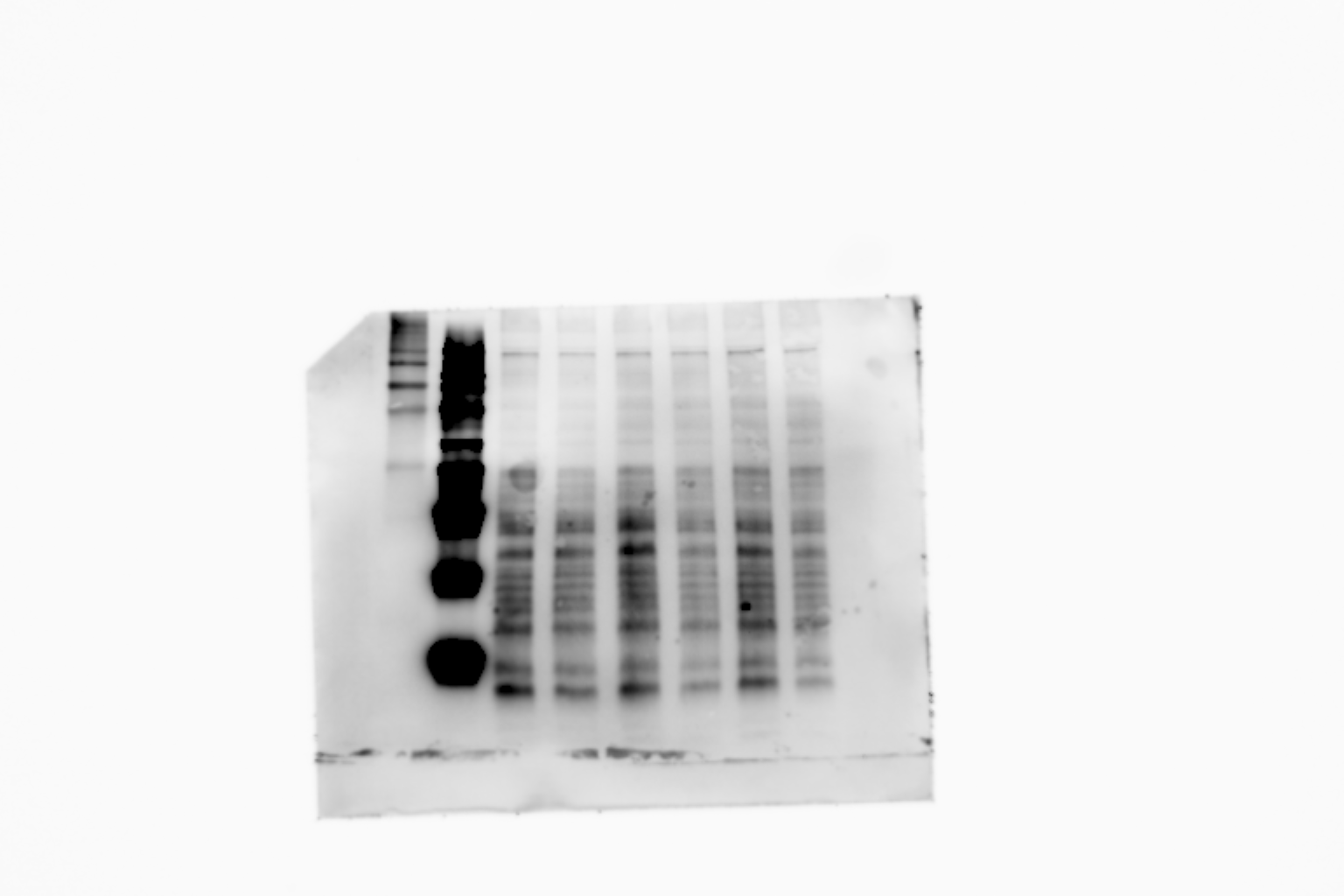

Supplement: S4 File — (ZIP) [file pone.0231910.s004.zip › S4_File/B/TIMP-1/Fig4B.TIMP1.70s .tif]

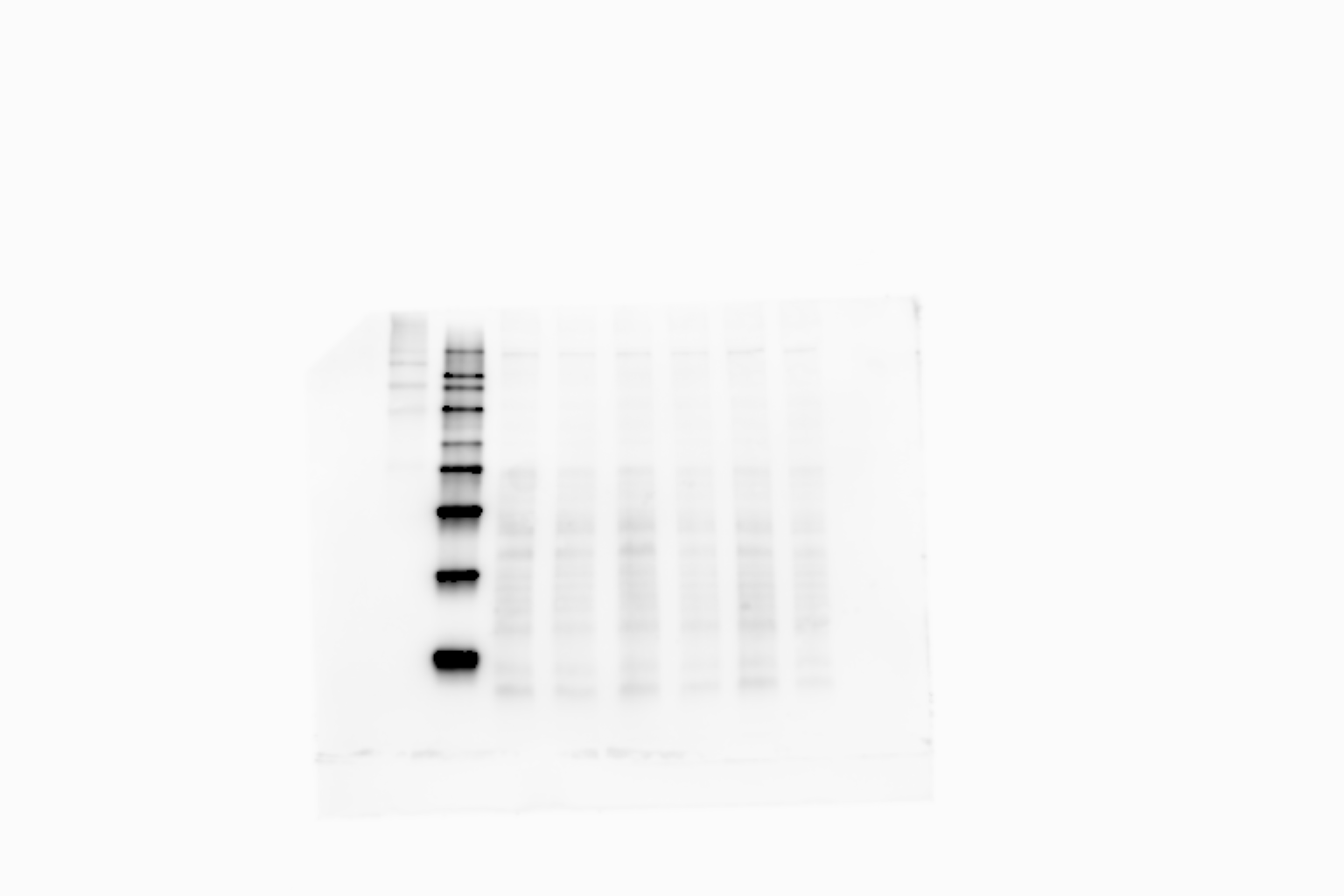

Supplement: S4 File — (ZIP) [file pone.0231910.s004.zip › S4_File/B/TIMP-1/WBexposure (each 10 sec)/Fig4B.TIMP1.10s.tif]

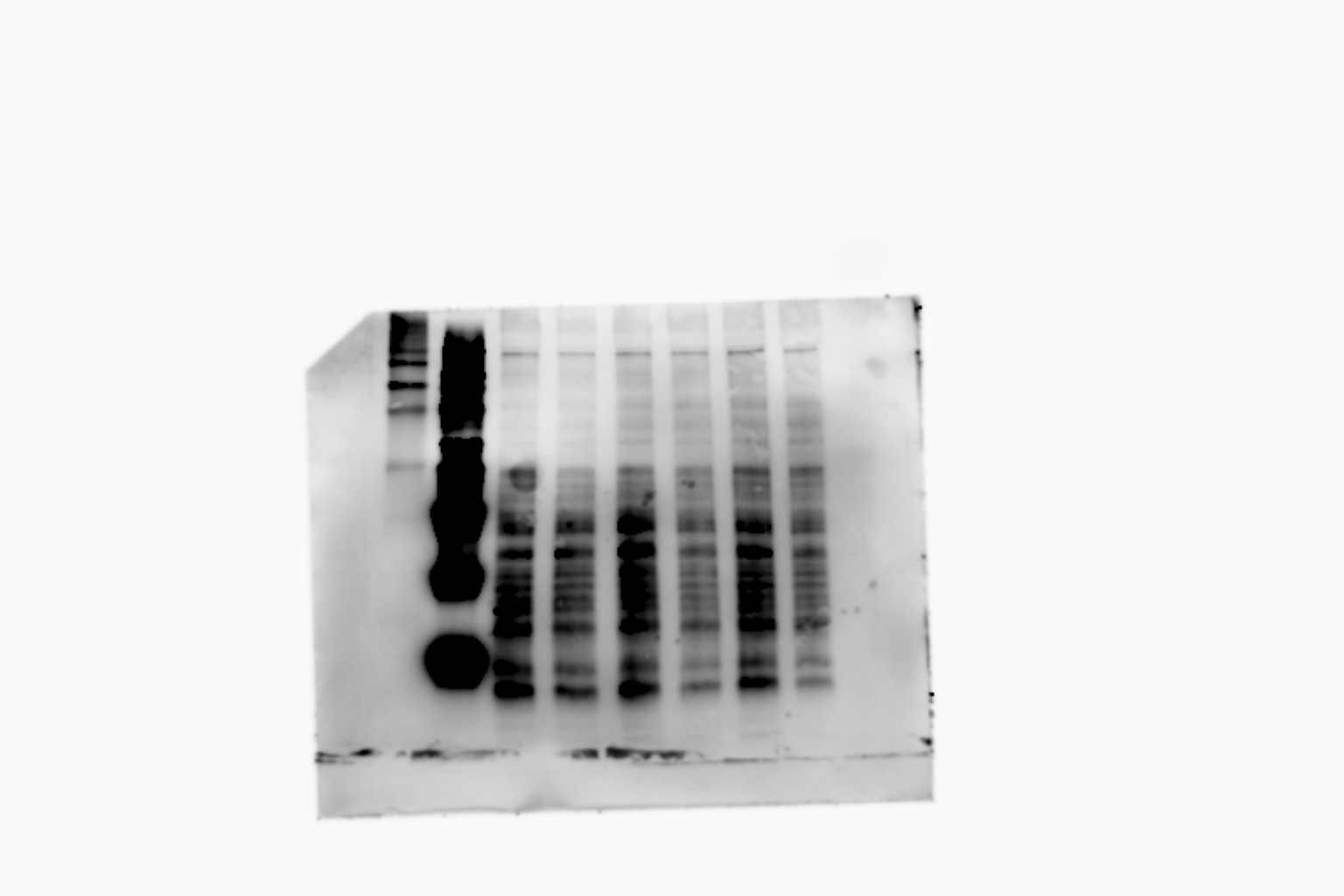

Supplement: S4 File — (ZIP) [file pone.0231910.s004.zip › S4_File/B/TIMP-1/WBexposure (each 10 sec)/Fig4B.TIMP1.110s.tif]

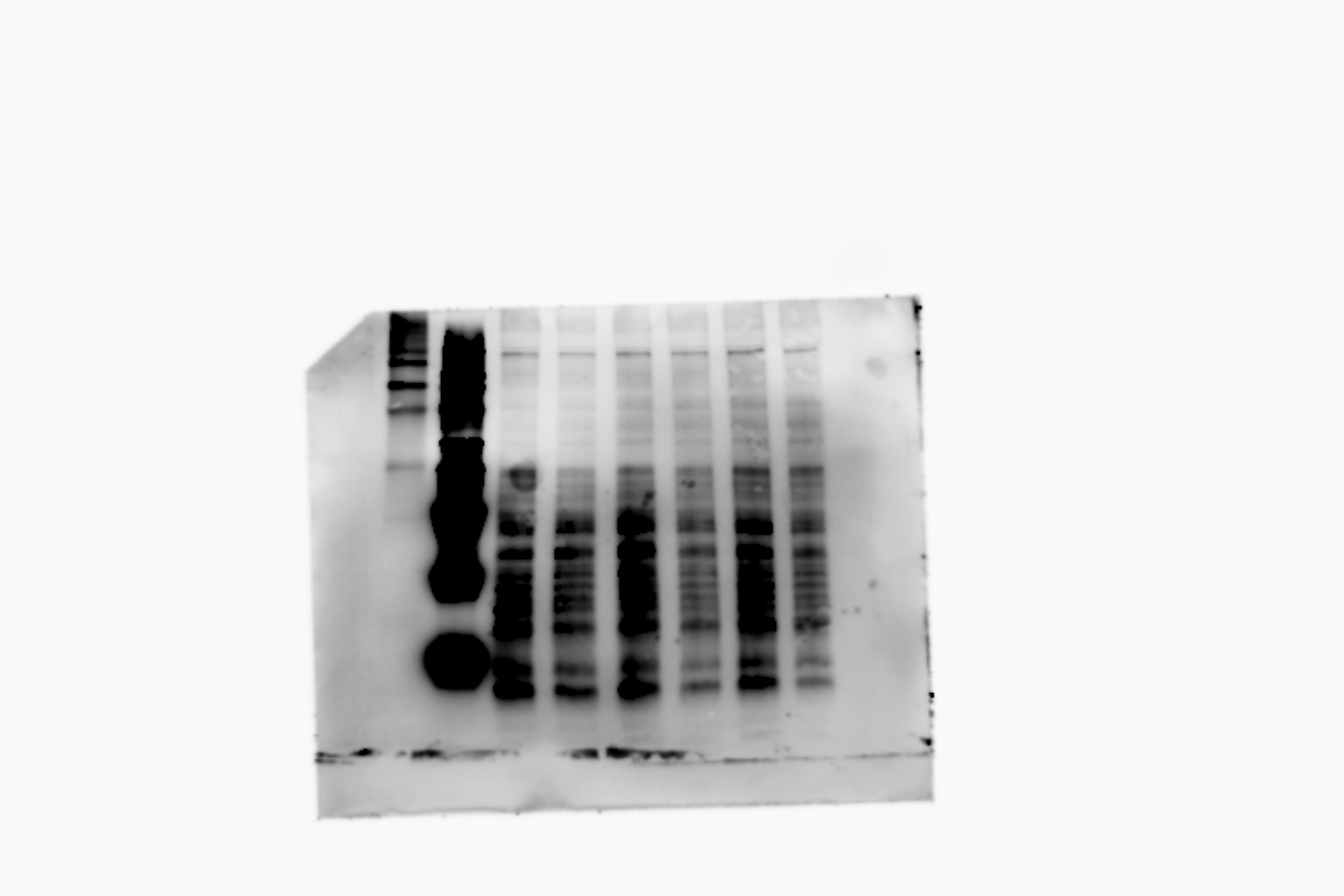

Supplement: S4 File — (ZIP) [file pone.0231910.s004.zip › S4_File/B/TIMP-1/WBexposure (each 10 sec)/Fig4B.TIMP1.120s.tif]

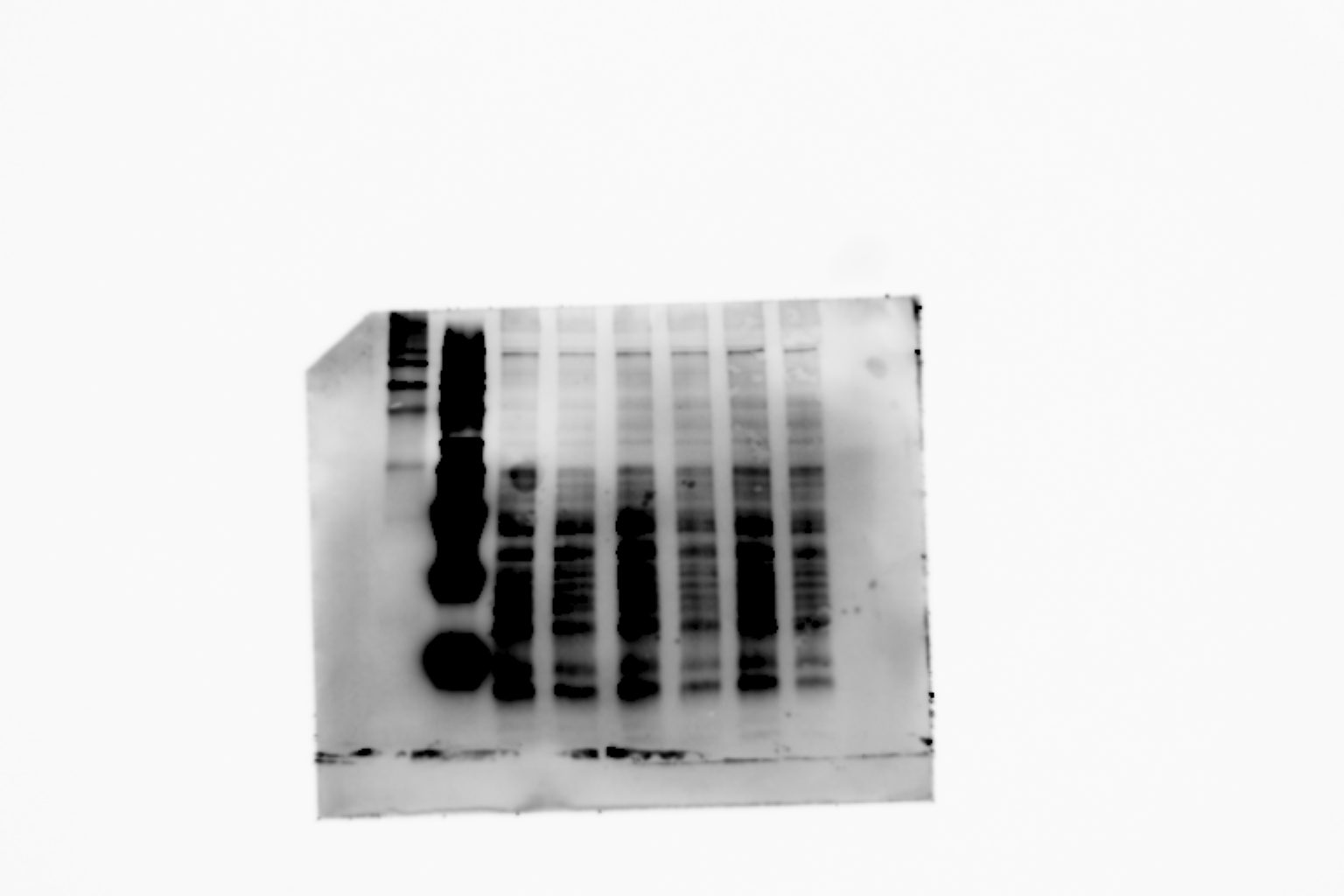

Supplement: S4 File — (ZIP) [file pone.0231910.s004.zip › S4_File/B/TIMP-1/WBexposure (each 10 sec)/Fig4B.TIMP1.130s.tif]

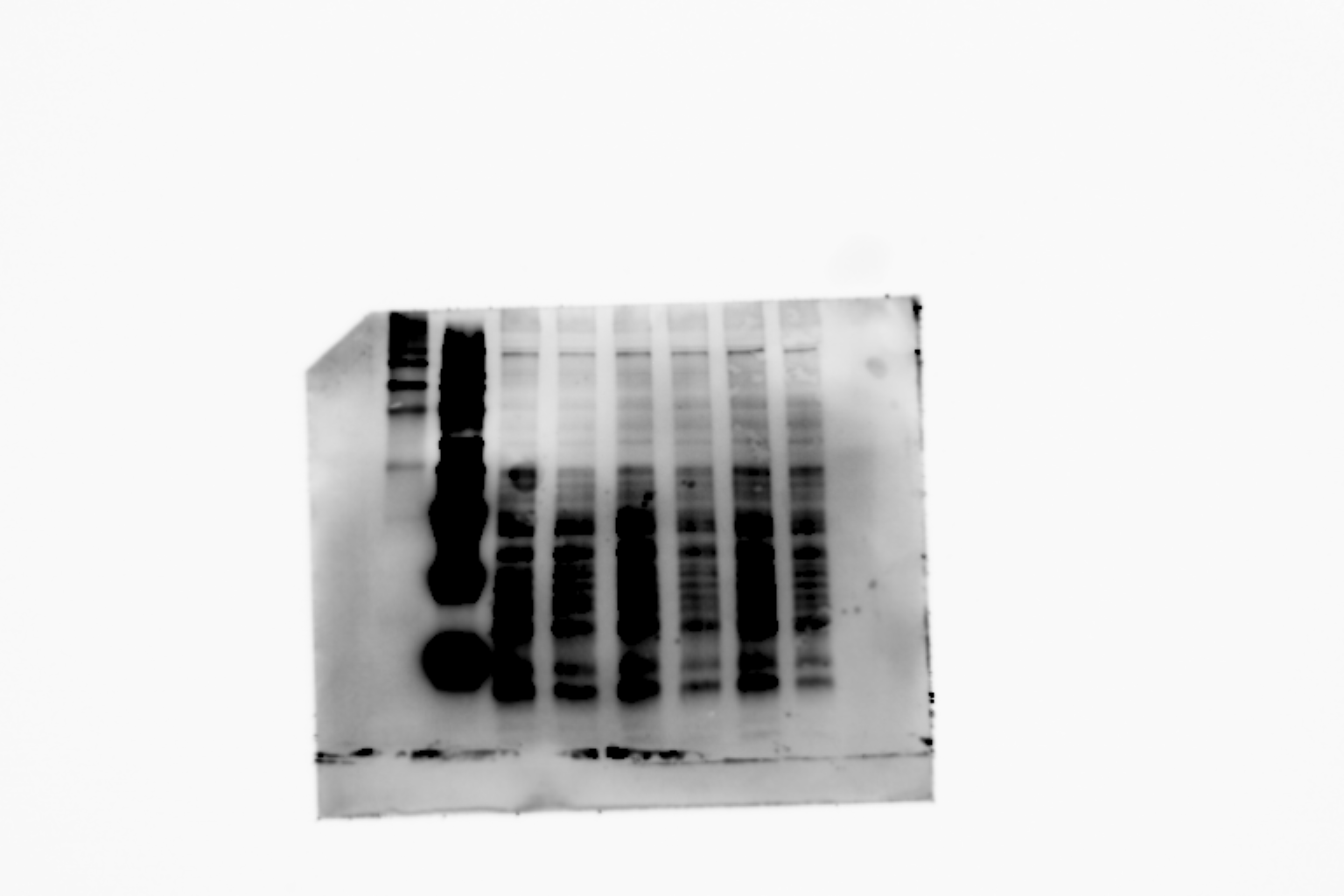

Supplement: S4 File — (ZIP) [file pone.0231910.s004.zip › S4_File/B/TIMP-1/WBexposure (each 10 sec)/Fig4B.TIMP1.140s.tif]

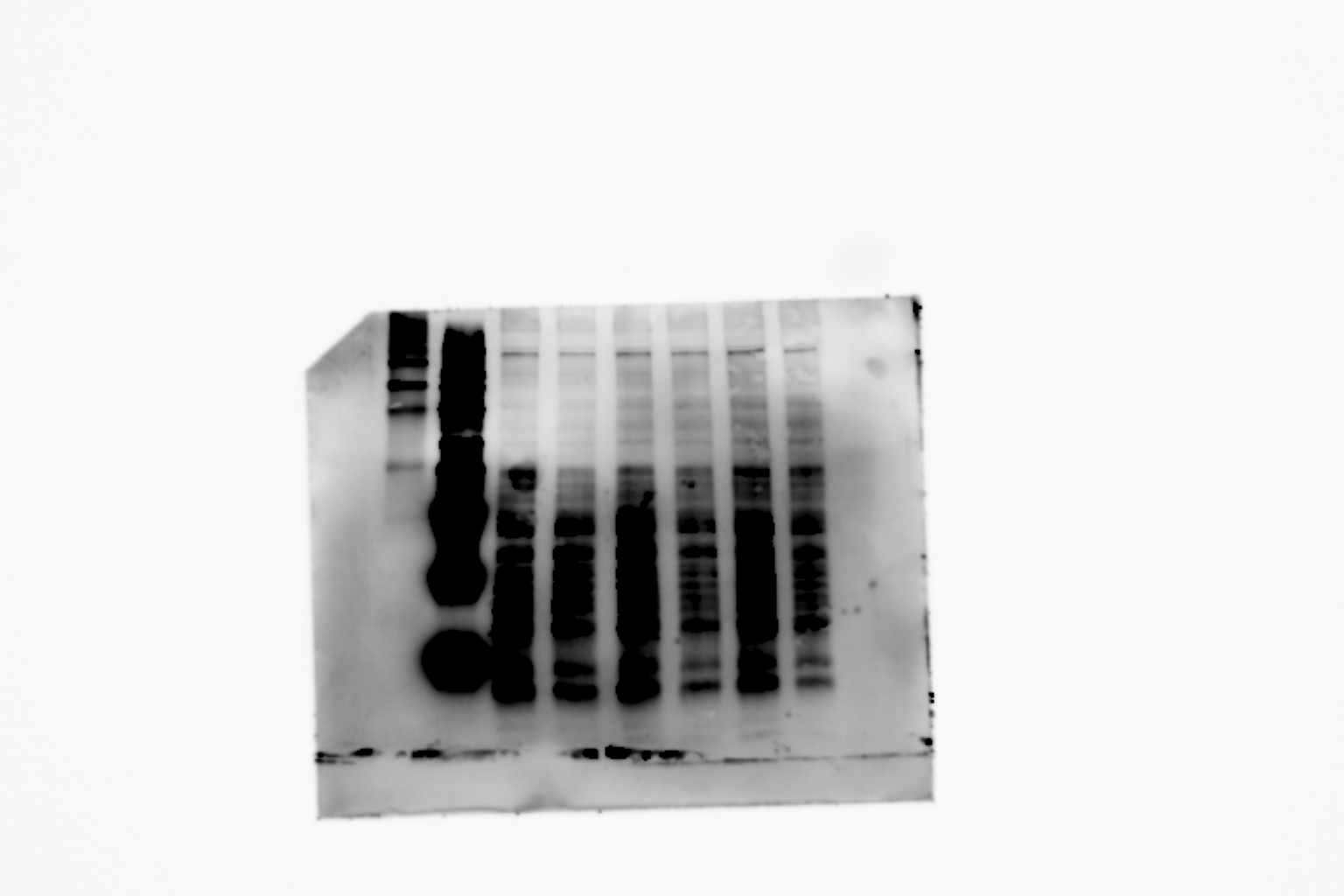

Supplement: S4 File — (ZIP) [file pone.0231910.s004.zip › S4_File/B/TIMP-1/WBexposure (each 10 sec)/Fig4B.TIMP1.150s.tif]

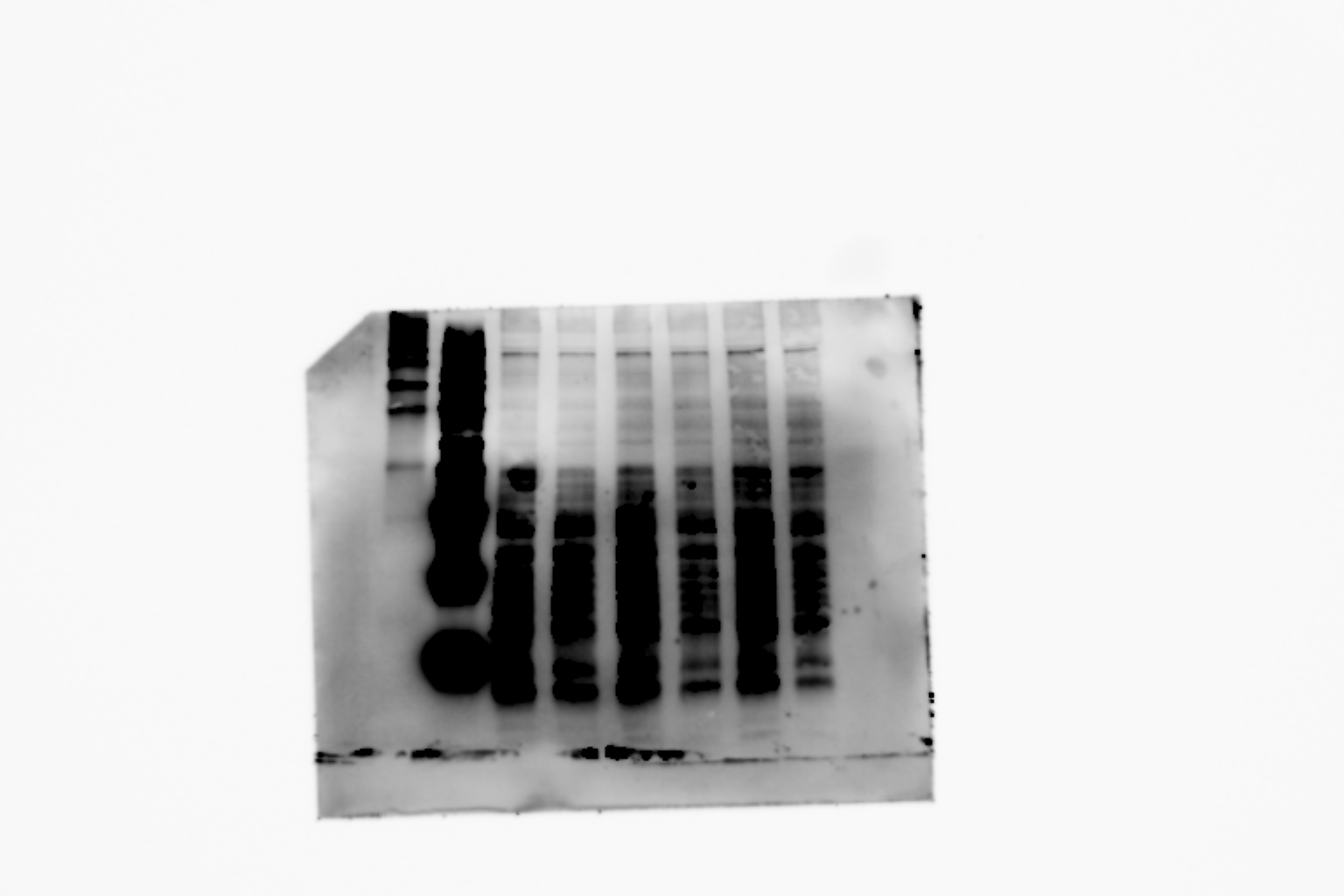

Supplement: S4 File — (ZIP) [file pone.0231910.s004.zip › S4_File/B/TIMP-1/WBexposure (each 10 sec)/Fig4B.TIMP1.160s.tif]

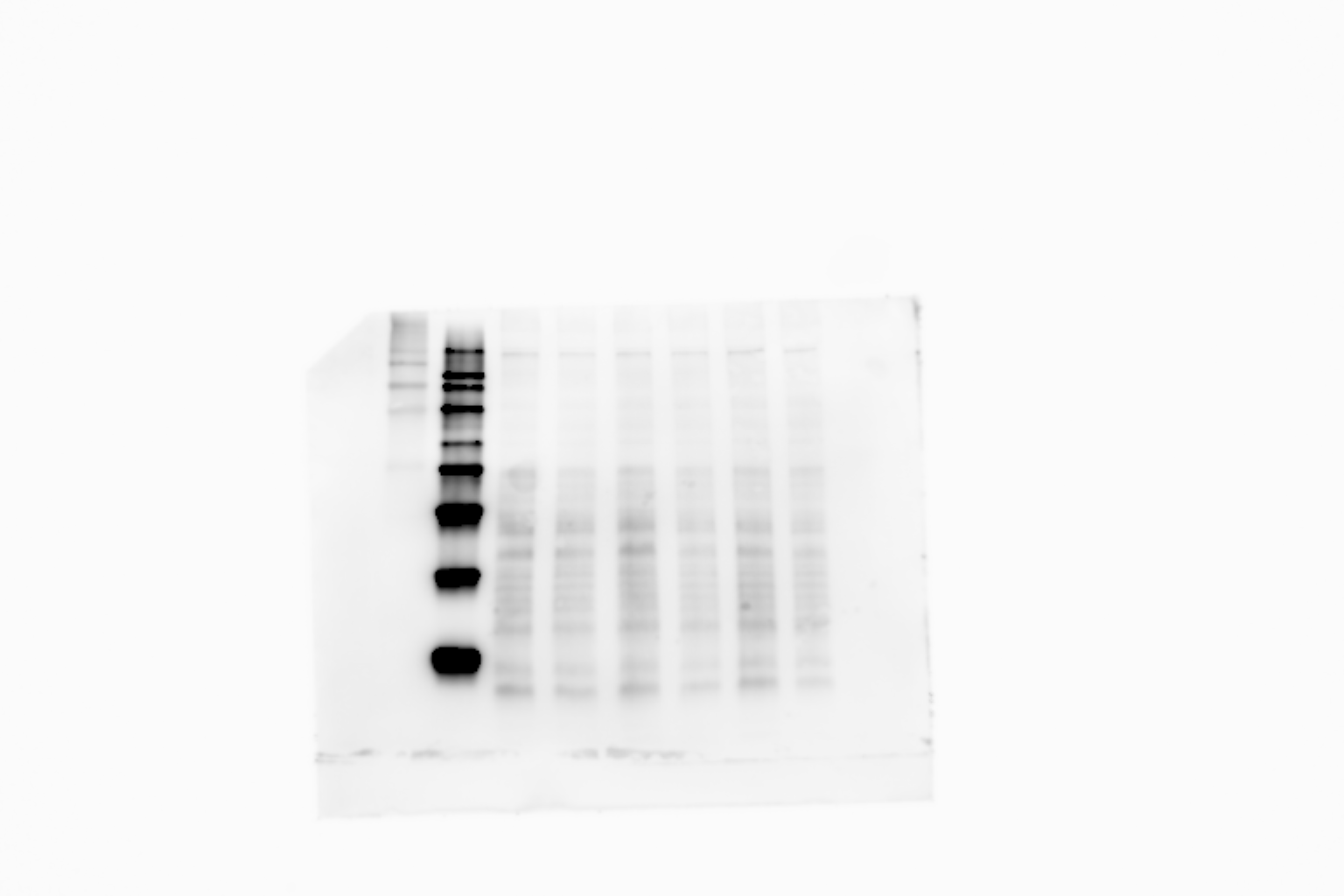

Supplement: S4 File — (ZIP) [file pone.0231910.s004.zip › S4_File/B/TIMP-1/WBexposure (each 10 sec)/Fig4B.TIMP1.20s.tif]

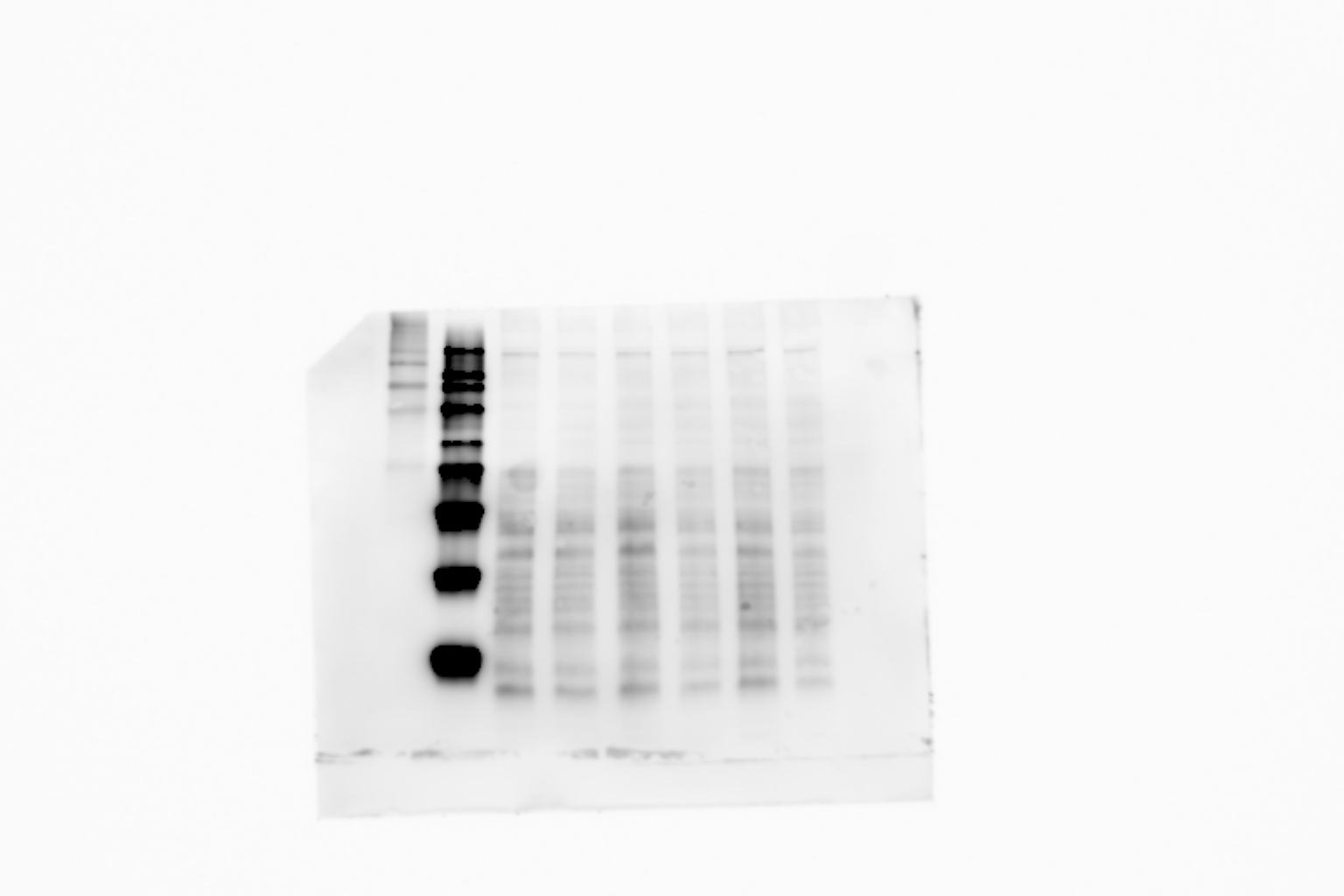

Supplement: S4 File — (ZIP) [file pone.0231910.s004.zip › S4_File/B/TIMP-1/WBexposure (each 10 sec)/Fig4B.TIMP1.30s.tif]

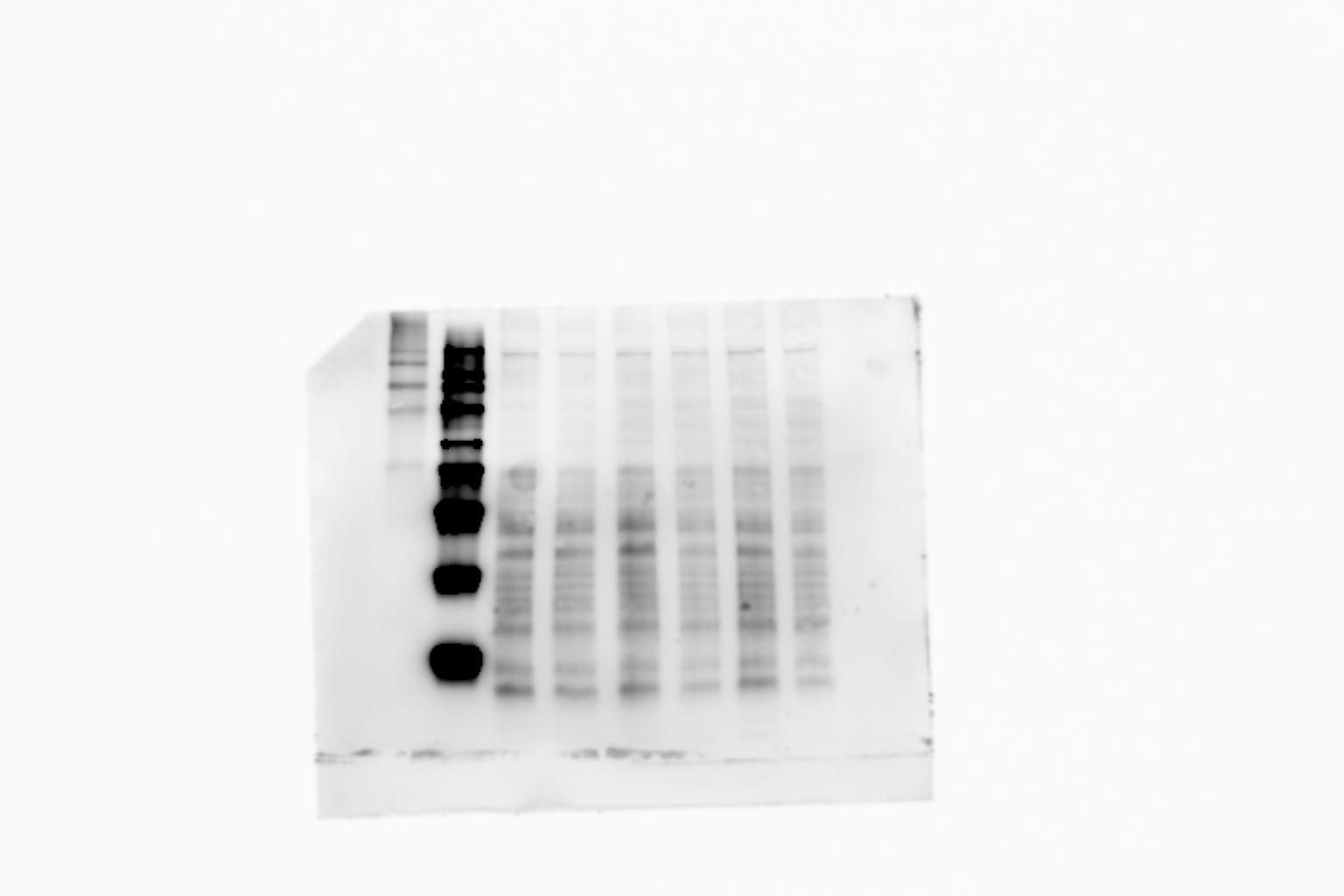

Supplement: S4 File — (ZIP) [file pone.0231910.s004.zip › S4_File/B/TIMP-1/WBexposure (each 10 sec)/Fig4B.TIMP1.40s.tif]

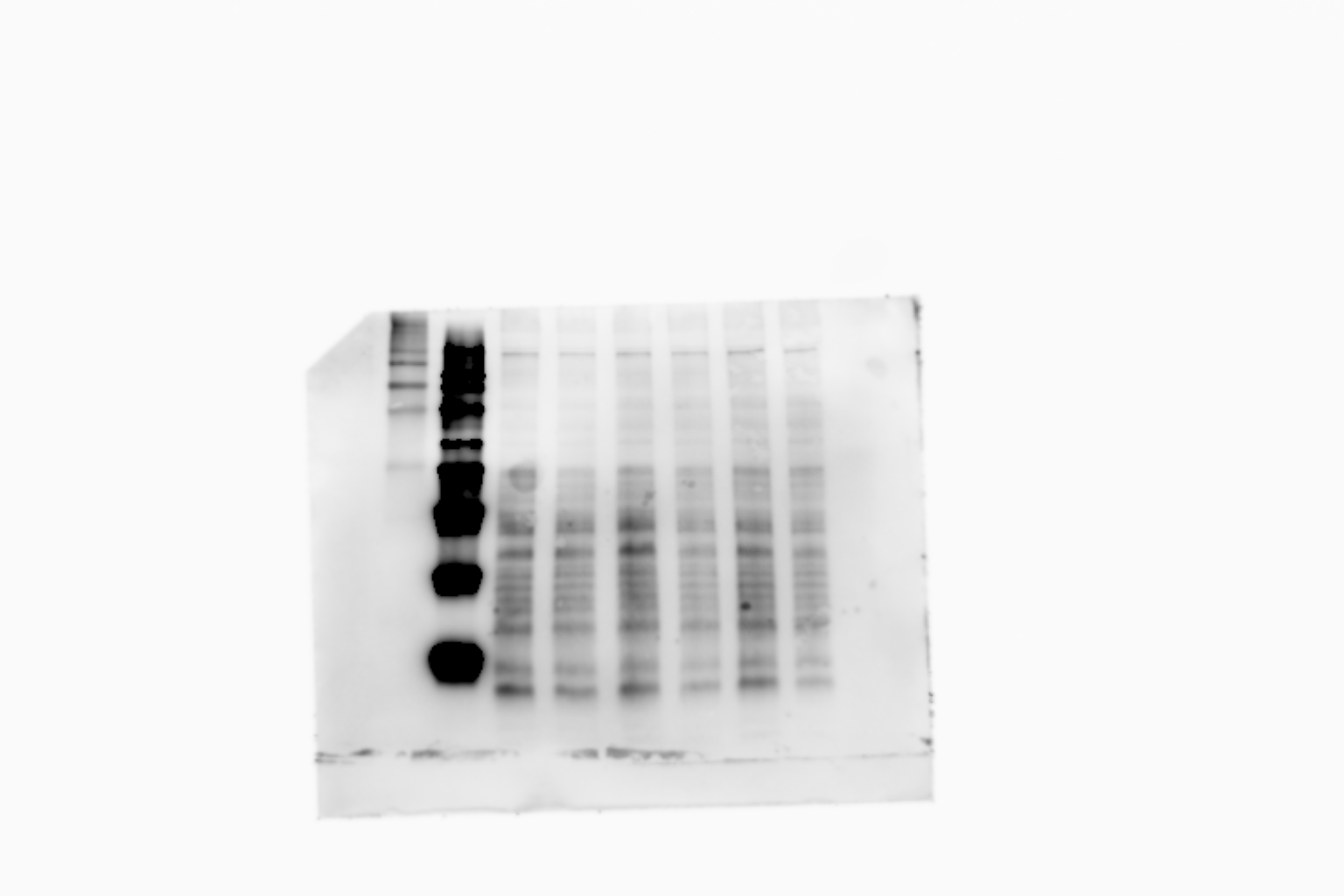

Supplement: S4 File — (ZIP) [file pone.0231910.s004.zip › S4_File/B/TIMP-1/WBexposure (each 10 sec)/Fig4B.TIMP1.50s.tif]

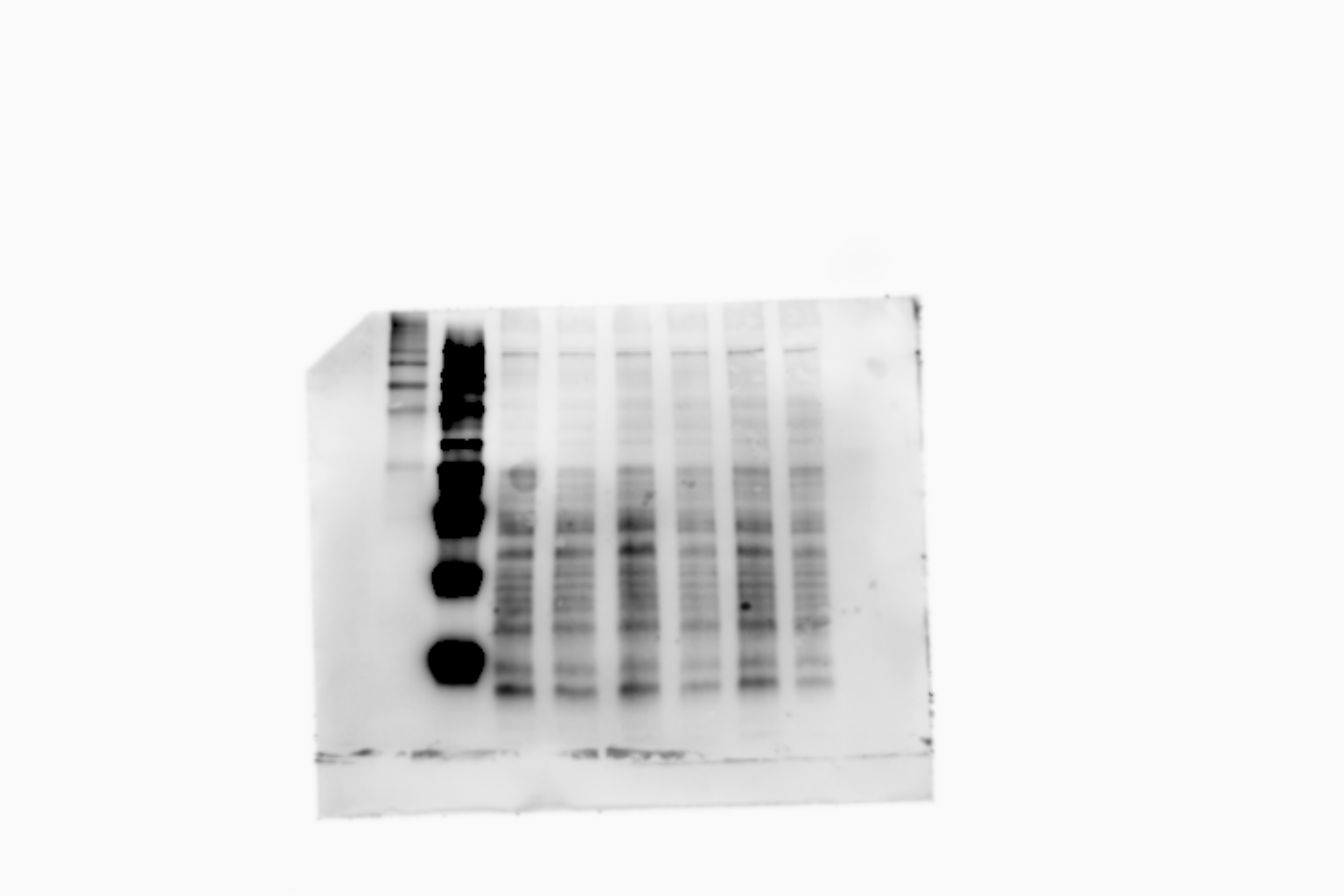

Supplement: S4 File — (ZIP) [file pone.0231910.s004.zip › S4_File/B/TIMP-1/WBexposure (each 10 sec)/Fig4B.TIMP1.60s.tif]

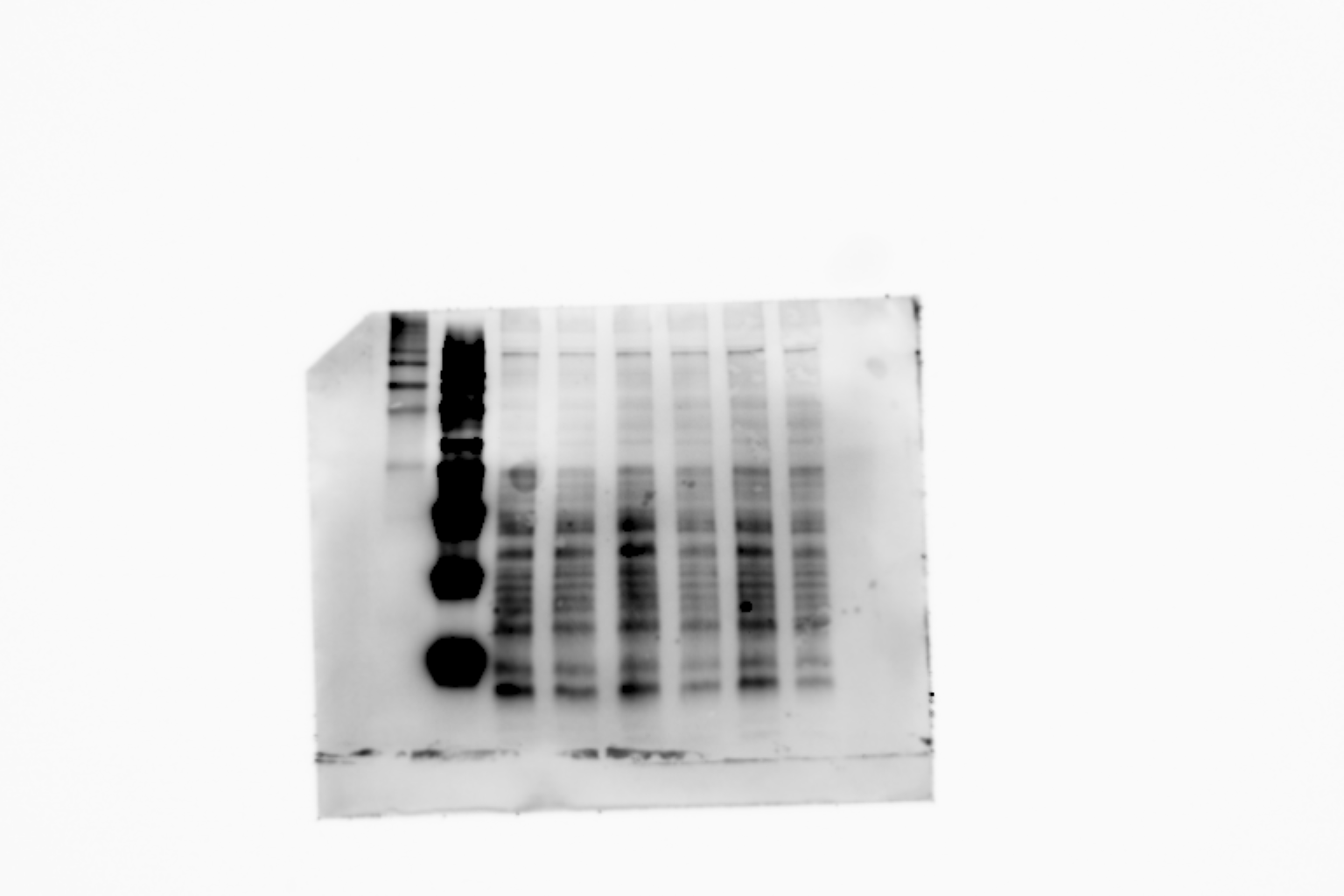

Supplement: S4 File — (ZIP) [file pone.0231910.s004.zip › S4_File/B/TIMP-1/WBexposure (each 10 sec)/Fig4B.TIMP1.80s.tif]

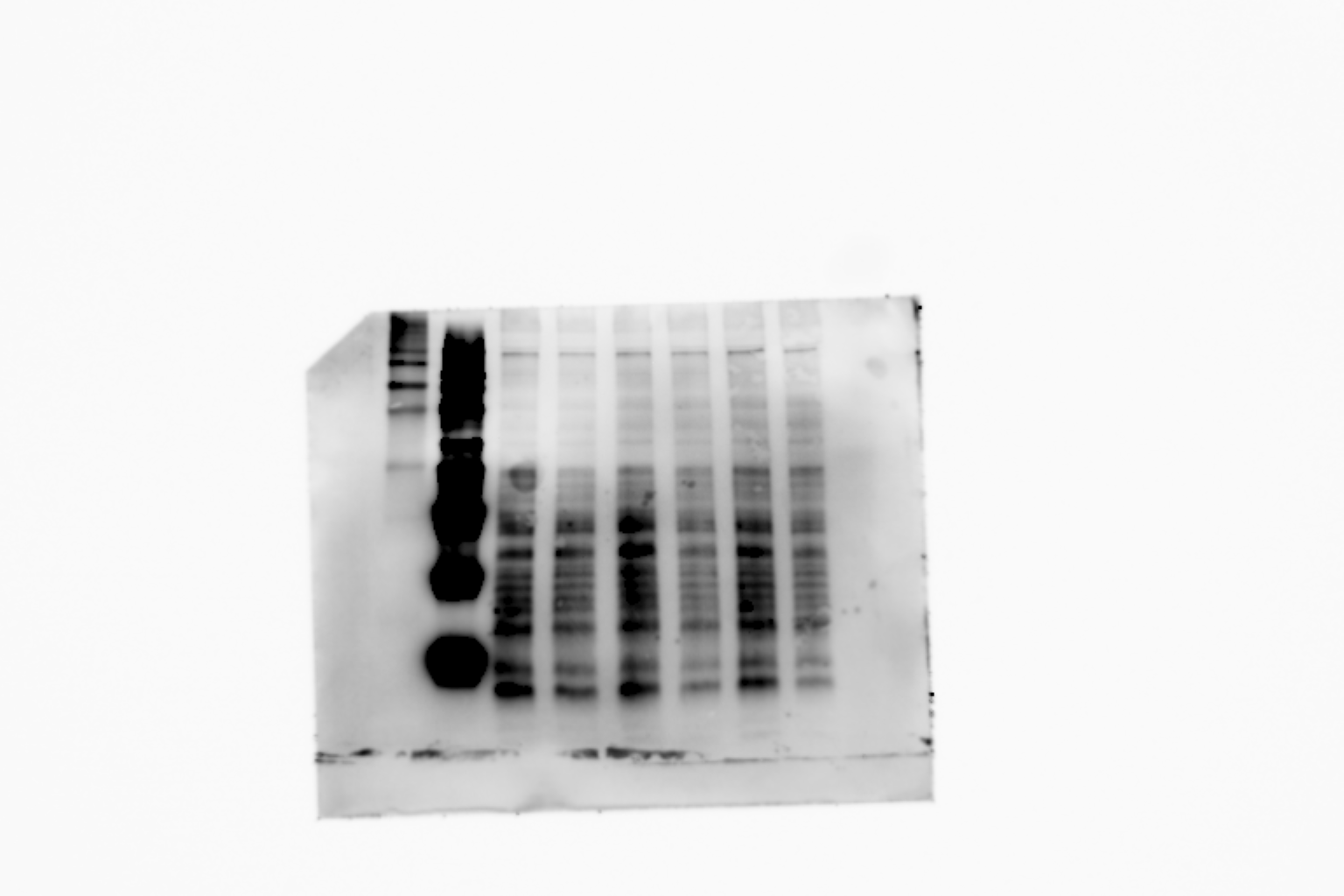

Supplement: S4 File — (ZIP) [file pone.0231910.s004.zip › S4_File/B/TIMP-1/WBexposure (each 10 sec)/Fig4B.TIMP1.90s.tif]

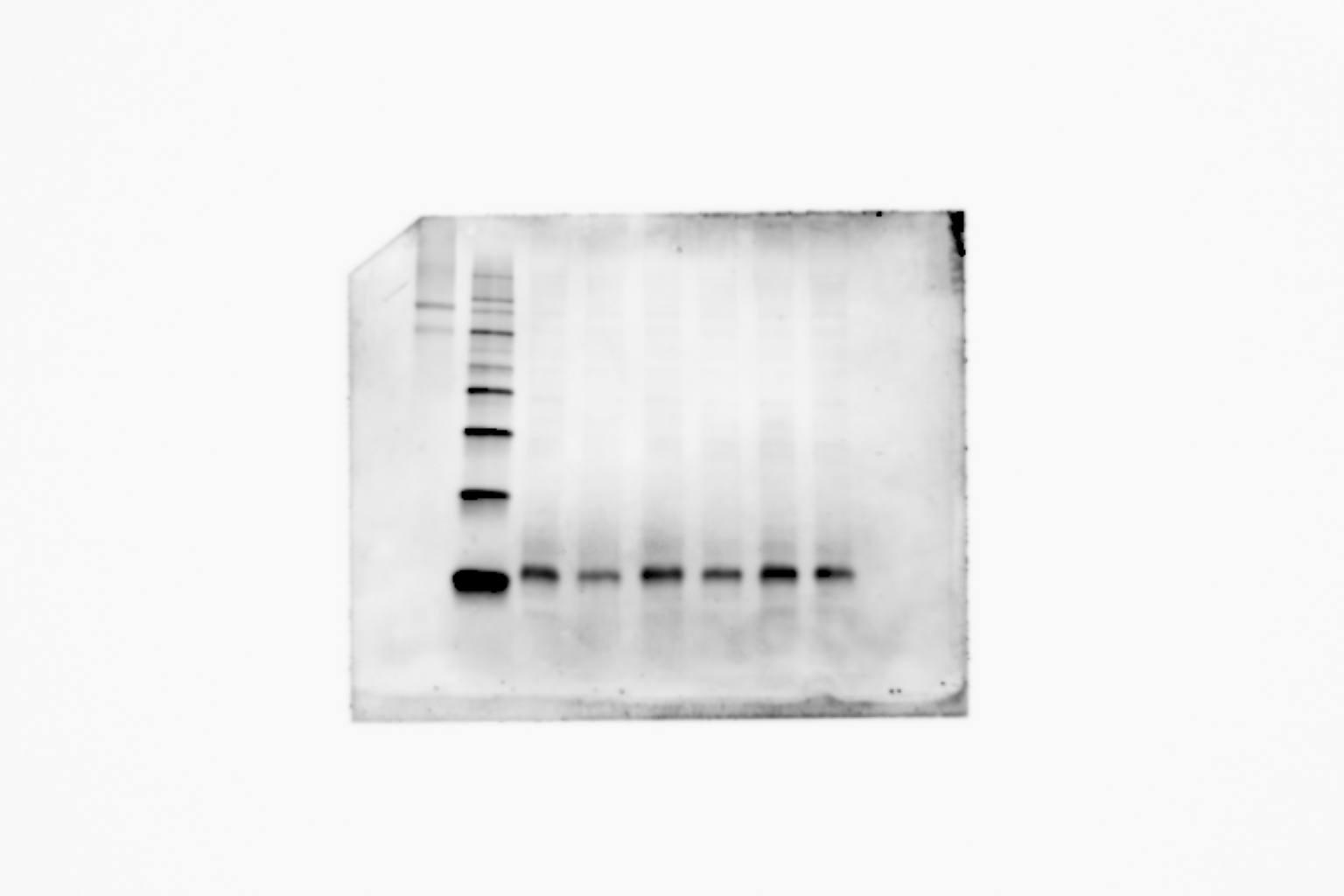

Supplement: S4 File — (ZIP) [file pone.0231910.s004.zip › S4_File/B/TIMP-2/Fig4B.TIMP2.140s.tif]

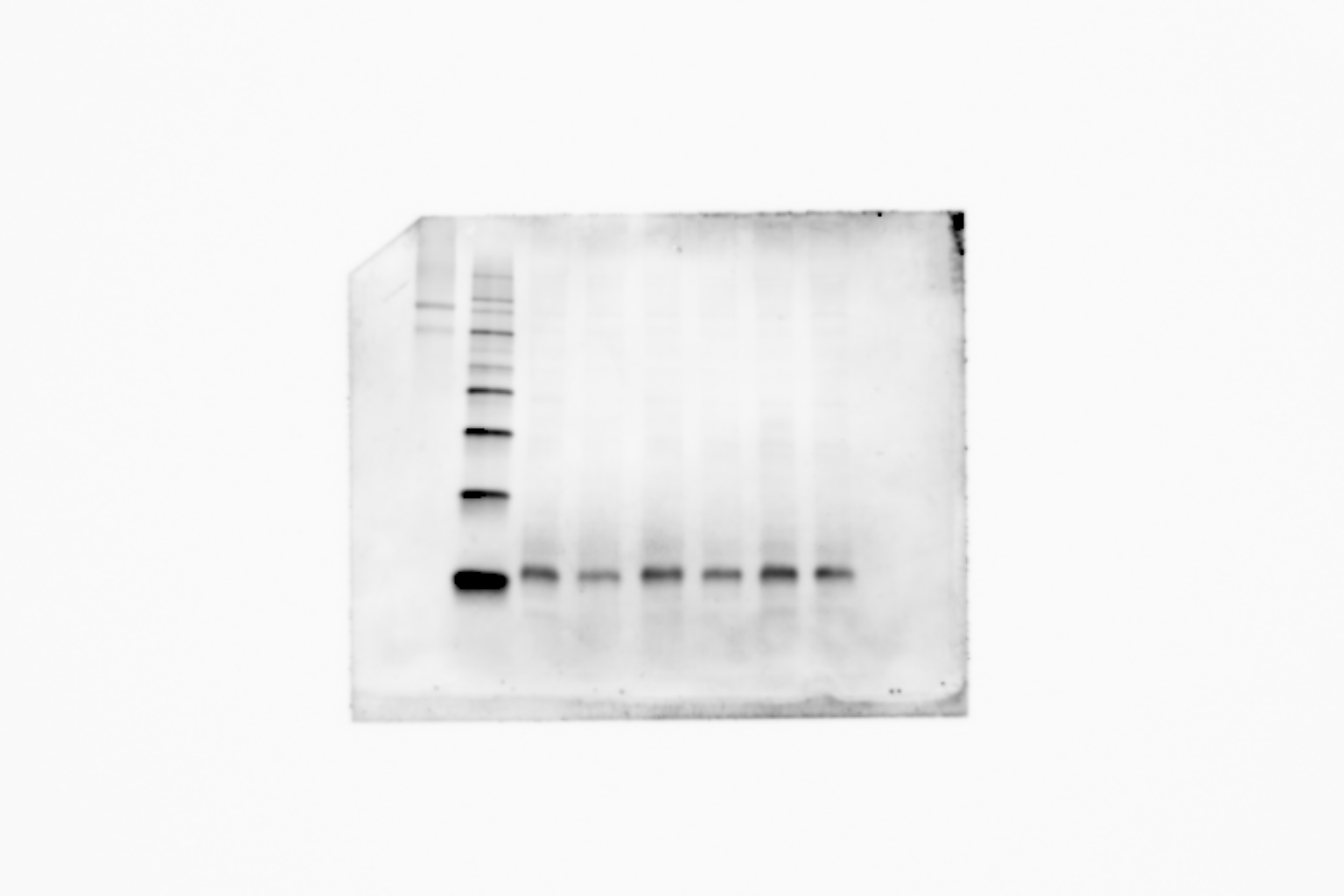

Supplement: S4 File — (ZIP) [file pone.0231910.s004.zip › S4_File/B/TIMP-2/WBexposure (each 10sec)/Fig4B.TIMP2.100s.tif]

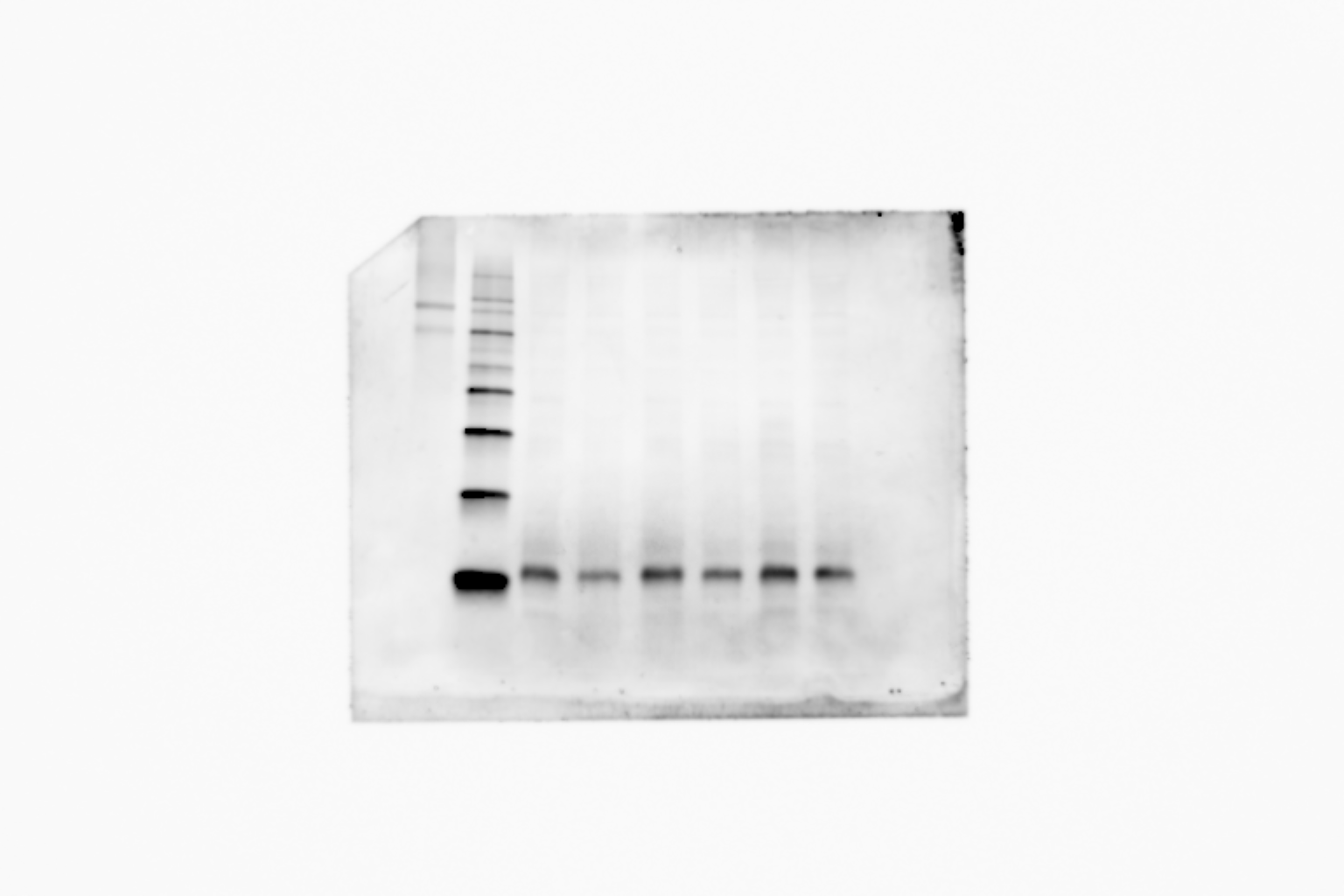

Supplement: S4 File — (ZIP) [file pone.0231910.s004.zip › S4_File/B/TIMP-2/WBexposure (each 10sec)/Fig4B.TIMP2.110s.tif]

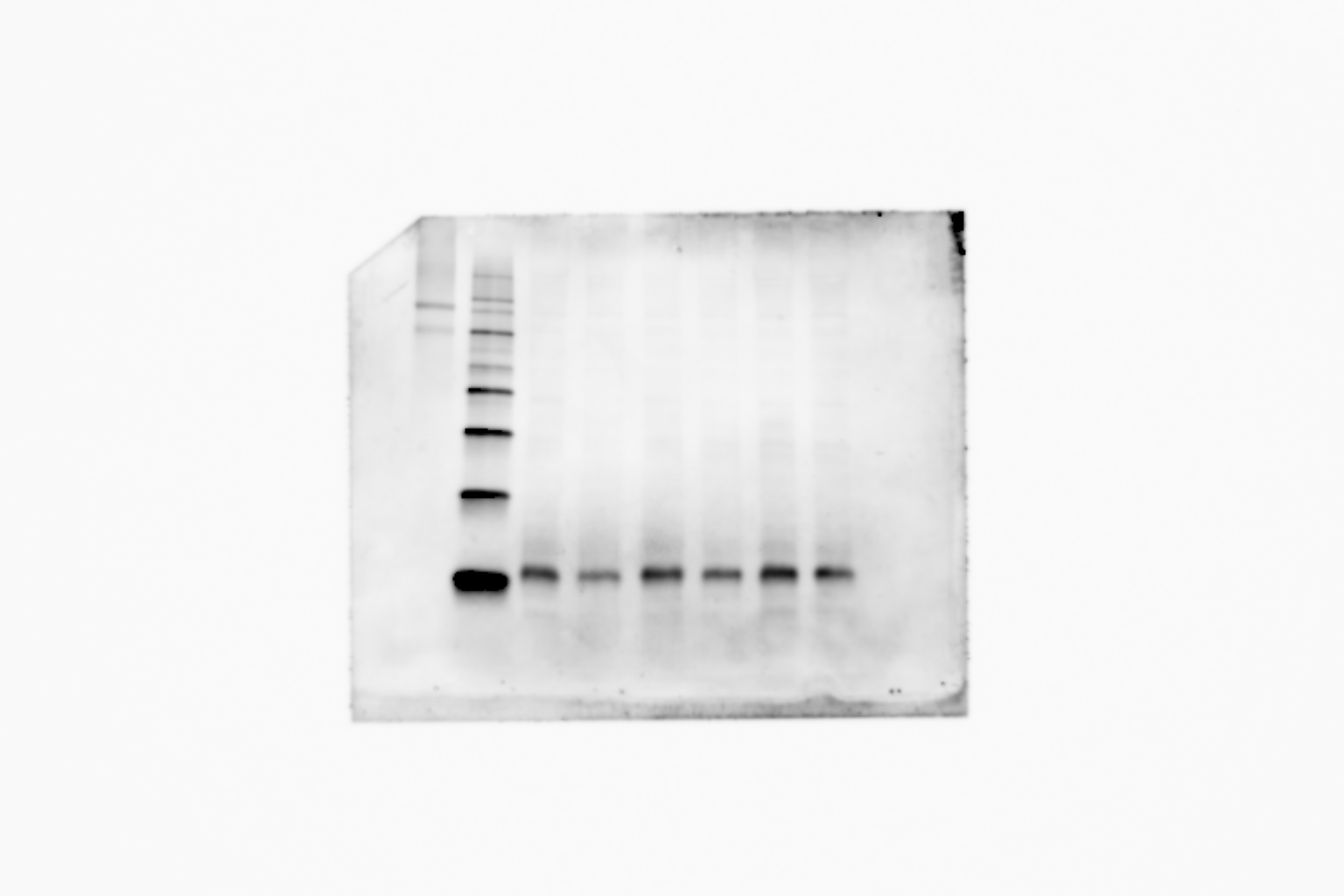

Supplement: S4 File — (ZIP) [file pone.0231910.s004.zip › S4_File/B/TIMP-2/WBexposure (each 10sec)/Fig4B.TIMP2.120s.tif]

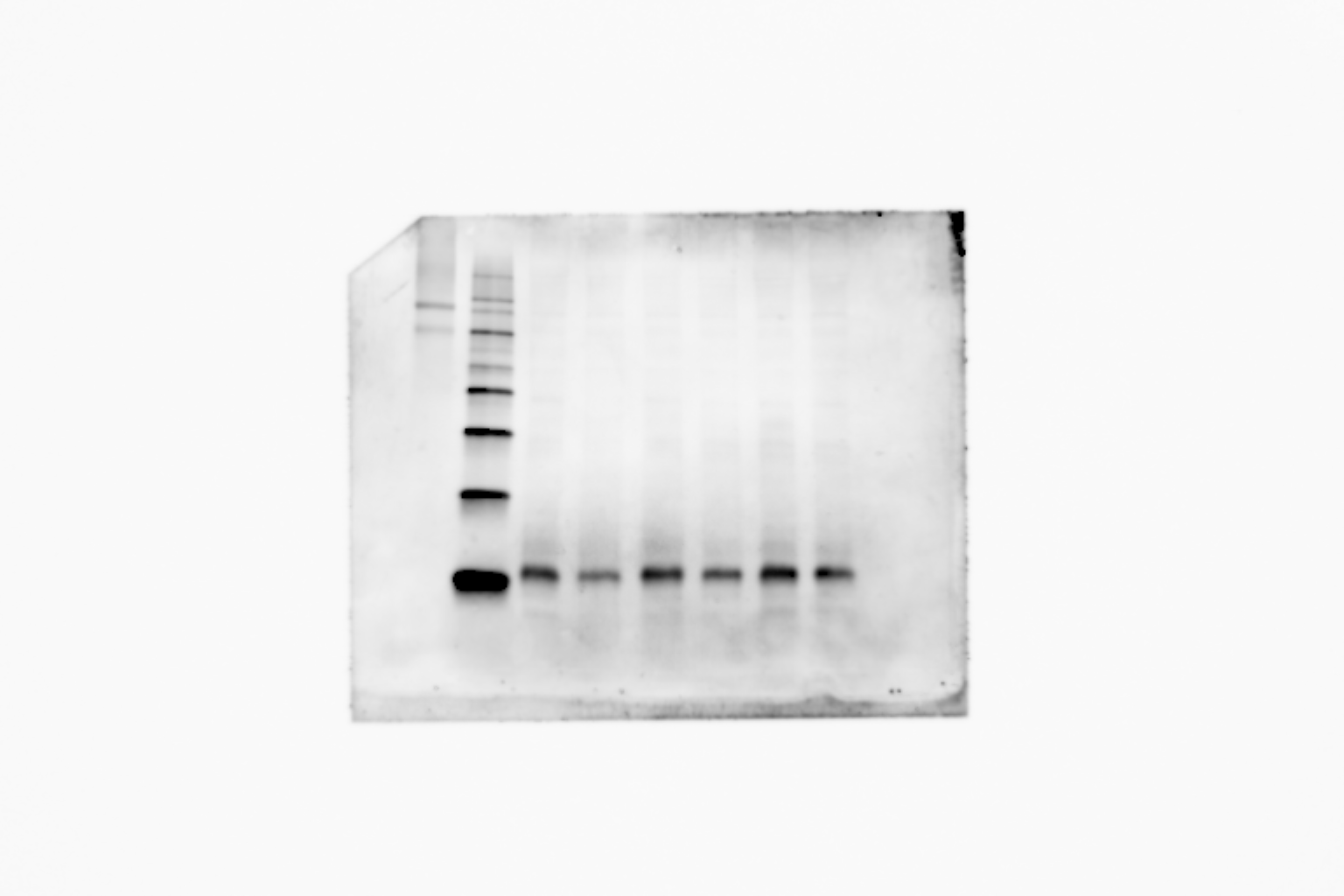

Supplement: S4 File — (ZIP) [file pone.0231910.s004.zip › S4_File/B/TIMP-2/WBexposure (each 10sec)/Fig4B.TIMP2.130s.tif]

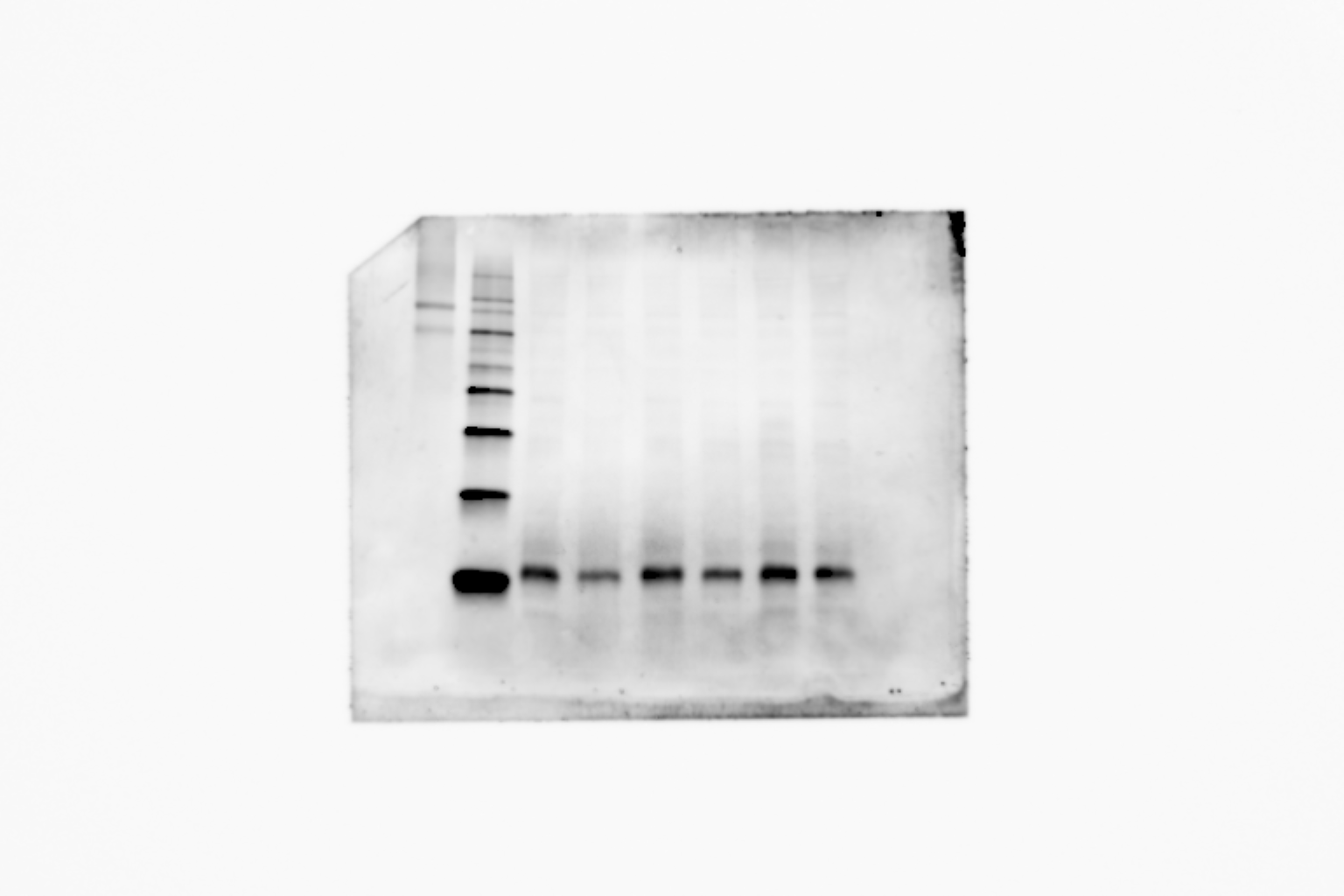

Supplement: S4 File — (ZIP) [file pone.0231910.s004.zip › S4_File/B/TIMP-2/WBexposure (each 10sec)/Fig4B.TIMP2.150s.tif]

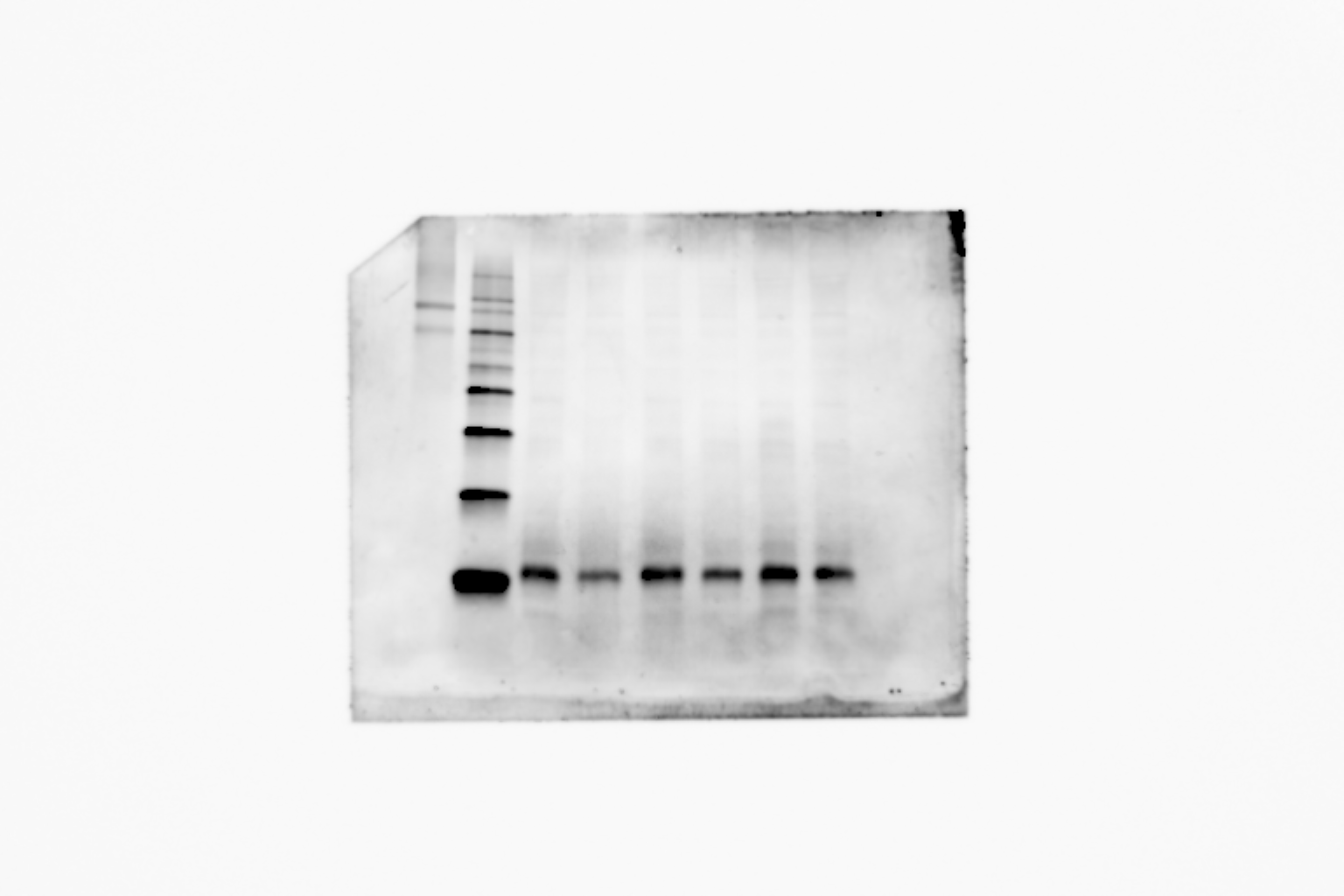

Supplement: S4 File — (ZIP) [file pone.0231910.s004.zip › S4_File/B/TIMP-2/WBexposure (each 10sec)/Fig4B.TIMP2.160s.tif]

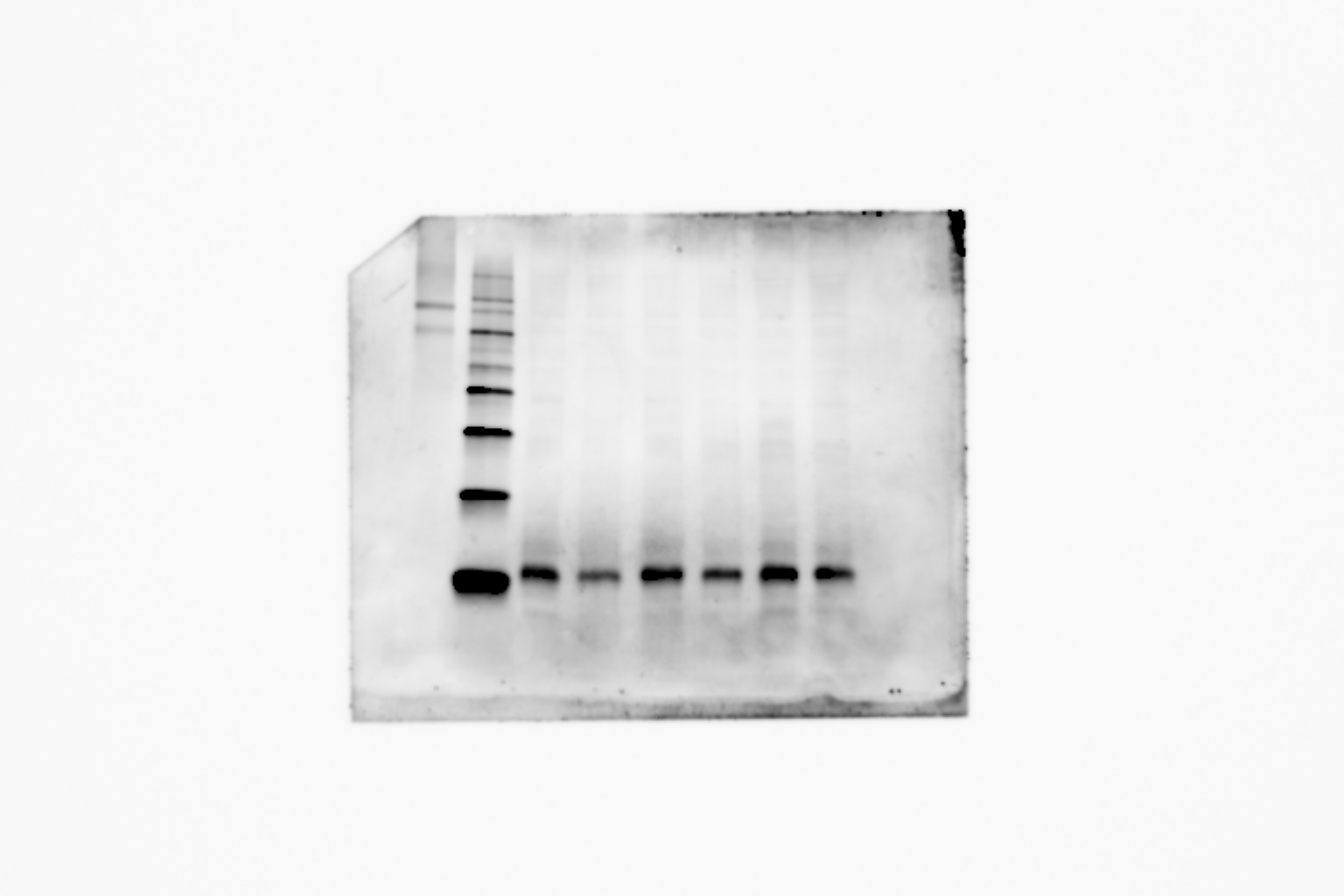

Supplement: S4 File — (ZIP) [file pone.0231910.s004.zip › S4_File/B/TIMP-2/WBexposure (each 10sec)/Fig4B.TIMP2.170s.tif]

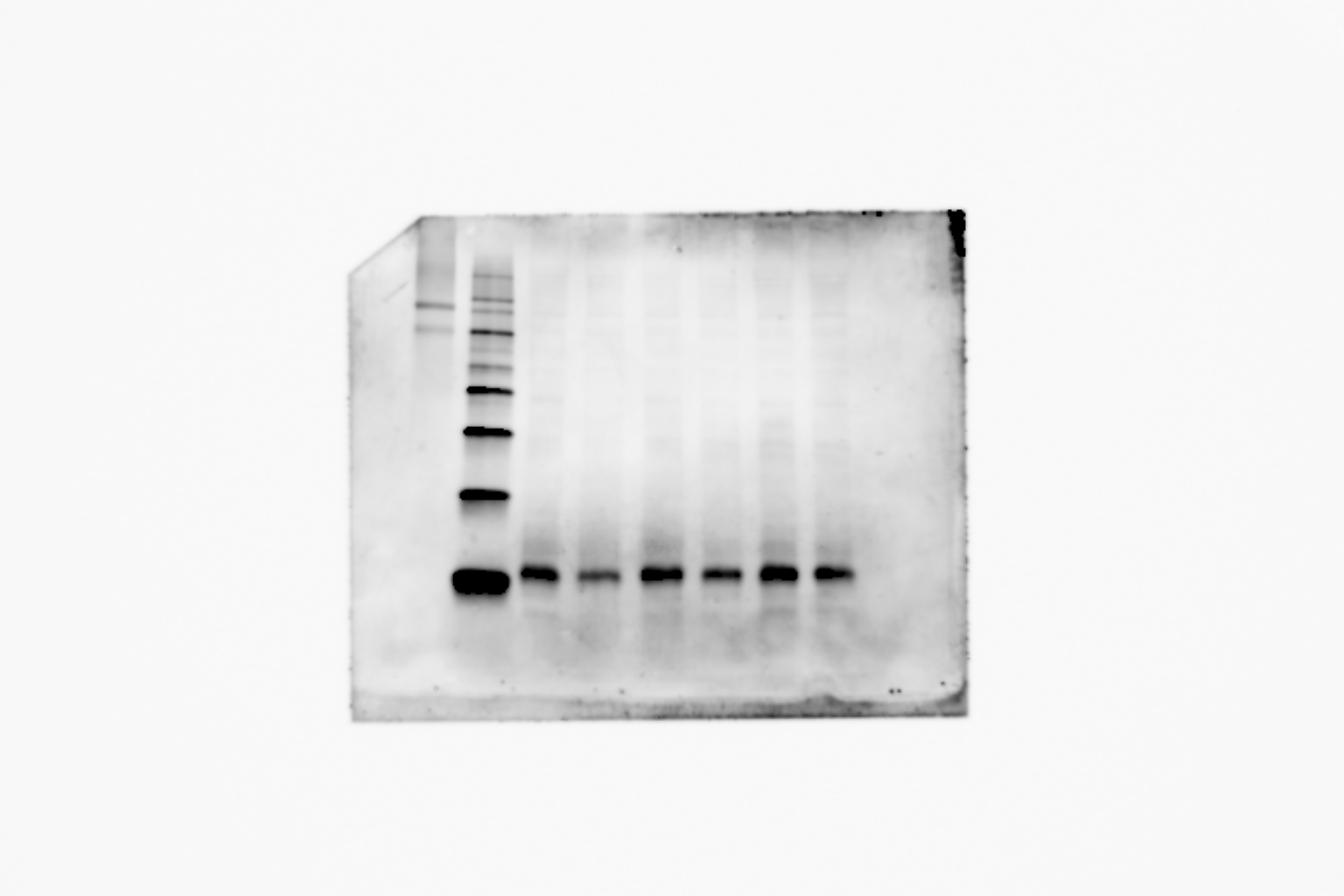

Supplement: S4 File — (ZIP) [file pone.0231910.s004.zip › S4_File/B/TIMP-2/WBexposure (each 10sec)/Fig4B.TIMP2.180s.tif]

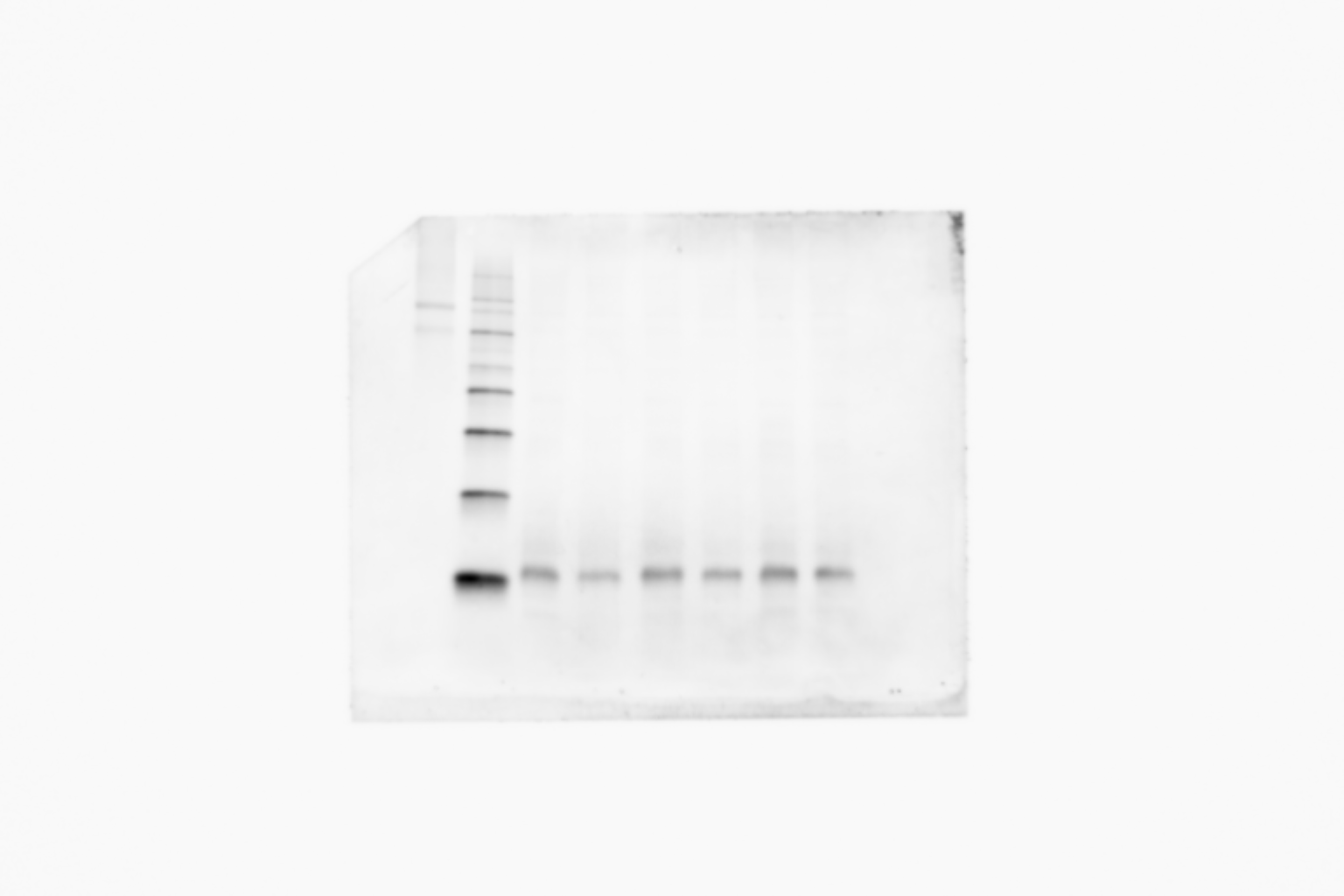

Supplement: S4 File — (ZIP) [file pone.0231910.s004.zip › S4_File/B/TIMP-2/WBexposure (each 10sec)/Fig4B.TIMP2.30s.tif]

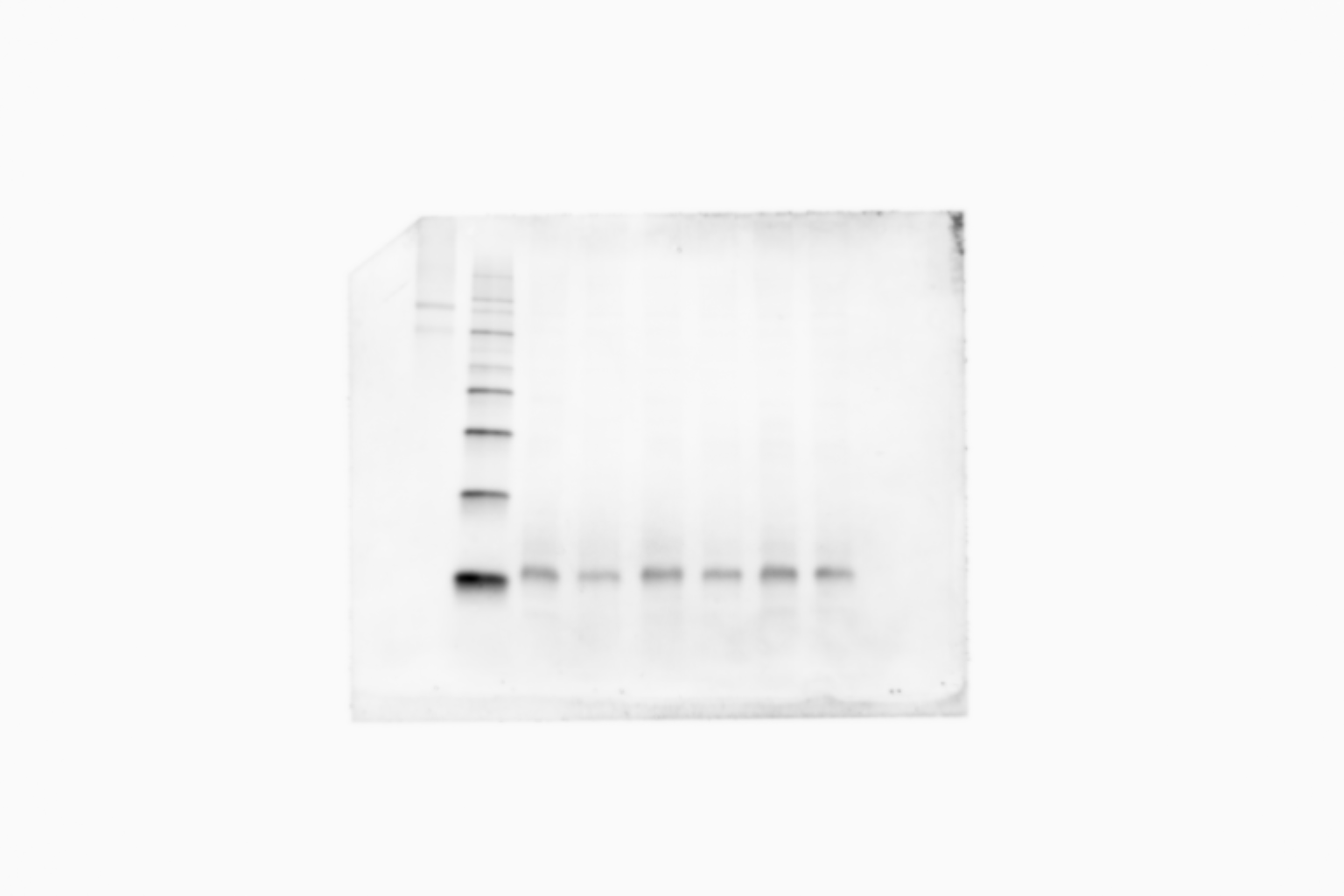

Supplement: S4 File — (ZIP) [file pone.0231910.s004.zip › S4_File/B/TIMP-2/WBexposure (each 10sec)/Fig4B.TIMP2.40s.tif]

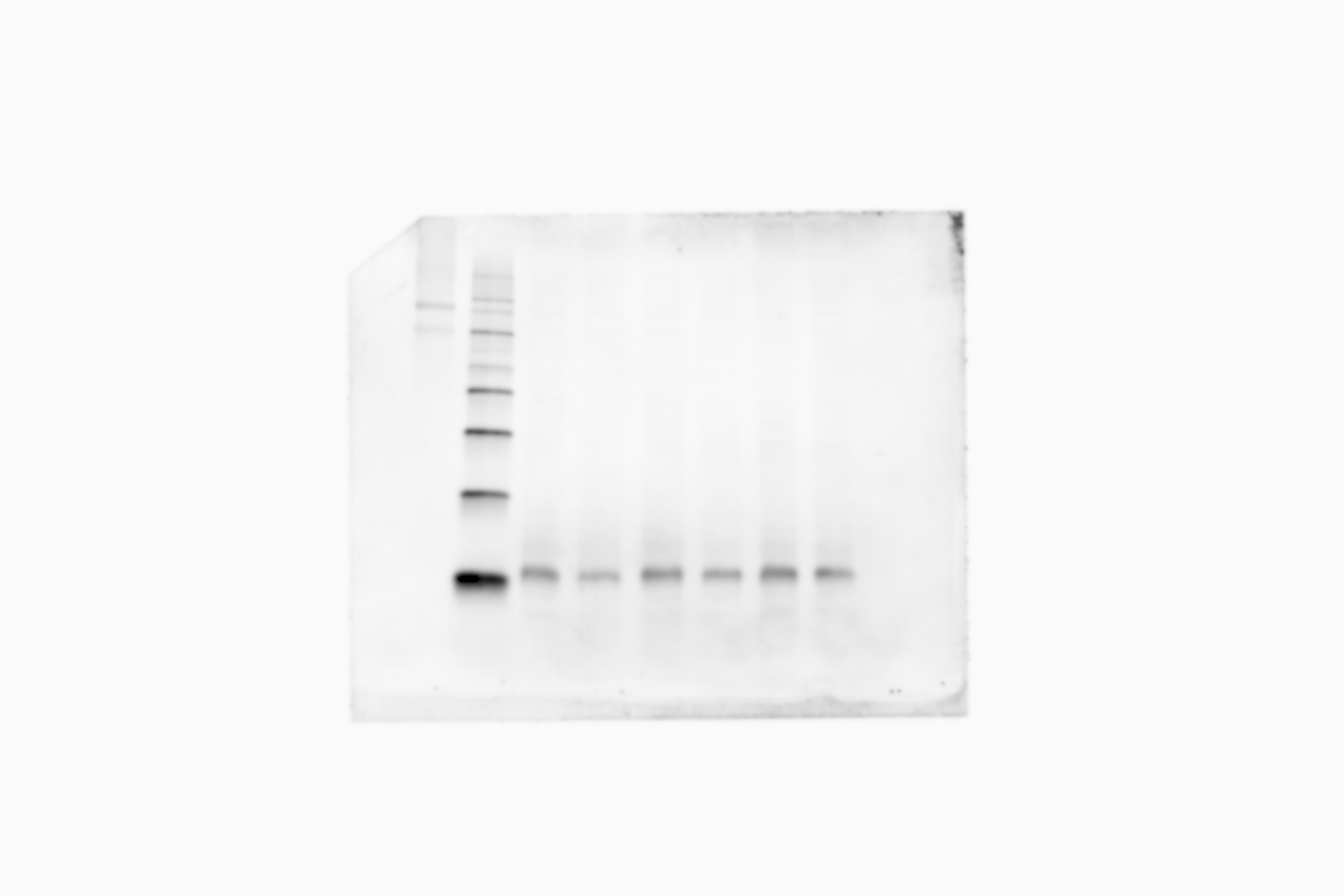

Supplement: S4 File — (ZIP) [file pone.0231910.s004.zip › S4_File/B/TIMP-2/WBexposure (each 10sec)/Fig4B.TIMP2.50s.tif]

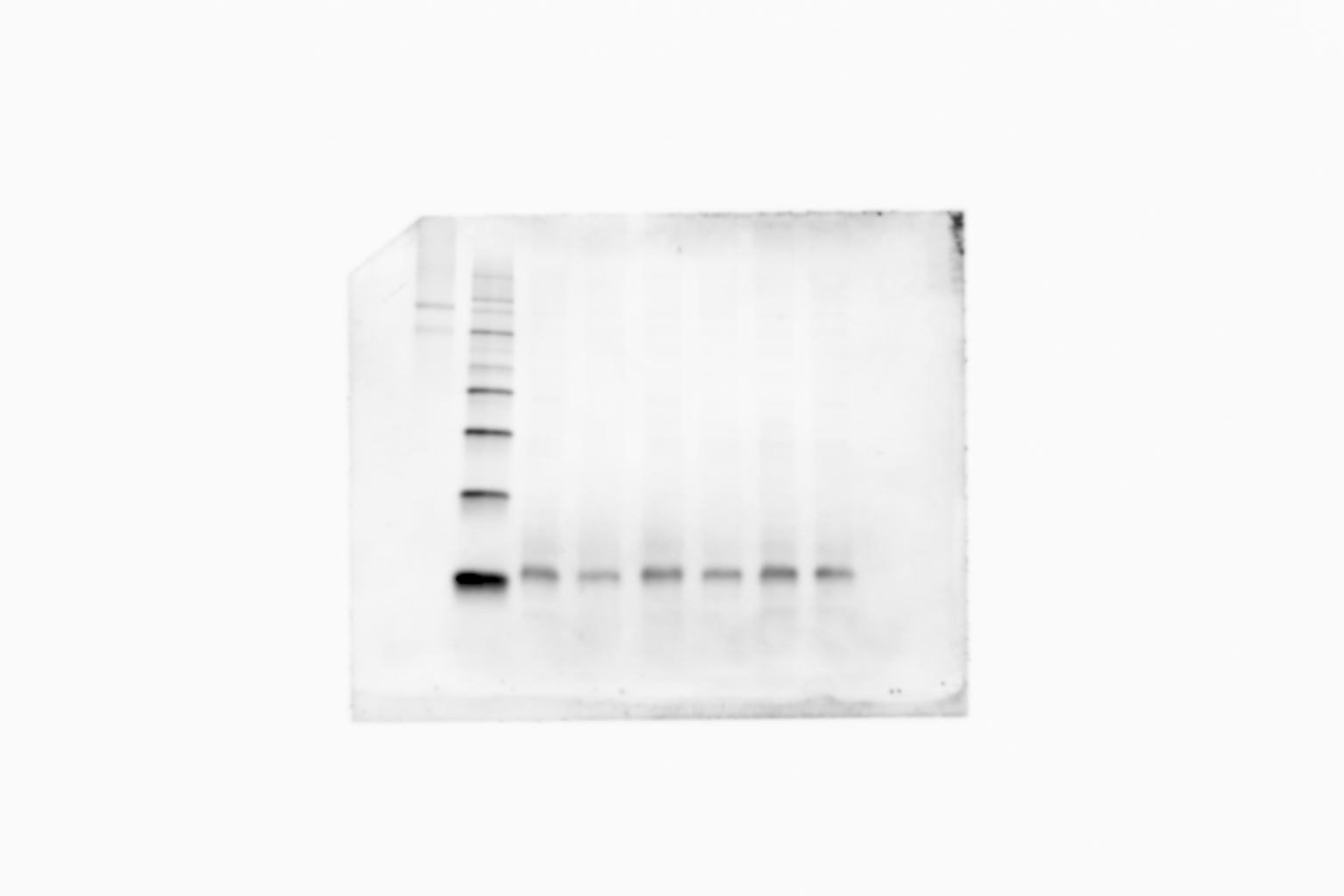

Supplement: S4 File — (ZIP) [file pone.0231910.s004.zip › S4_File/B/TIMP-2/WBexposure (each 10sec)/Fig4B.TIMP2.60s.tif]

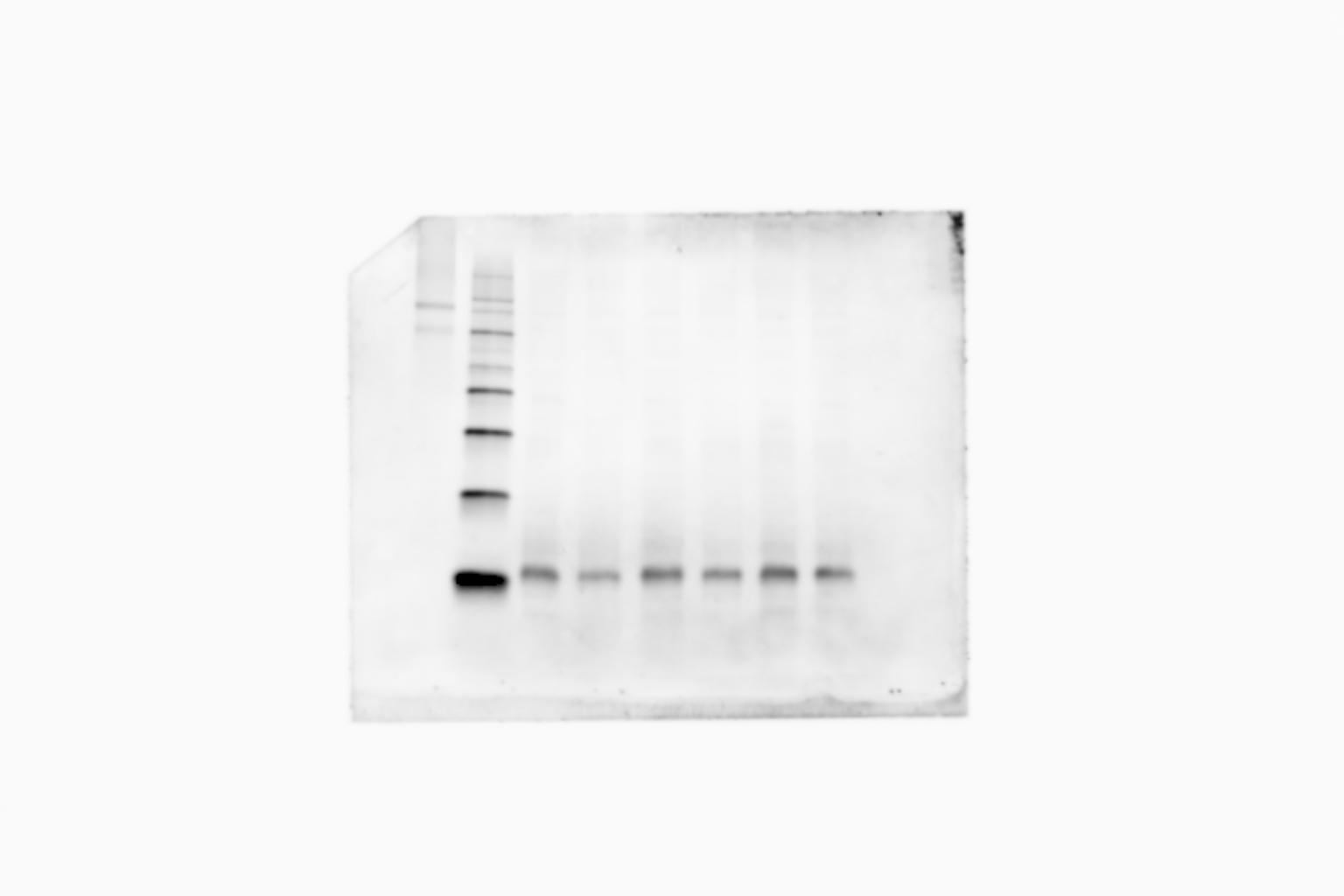

Supplement: S4 File — (ZIP) [file pone.0231910.s004.zip › S4_File/B/TIMP-2/WBexposure (each 10sec)/Fig4B.TIMP2.70s.tif]

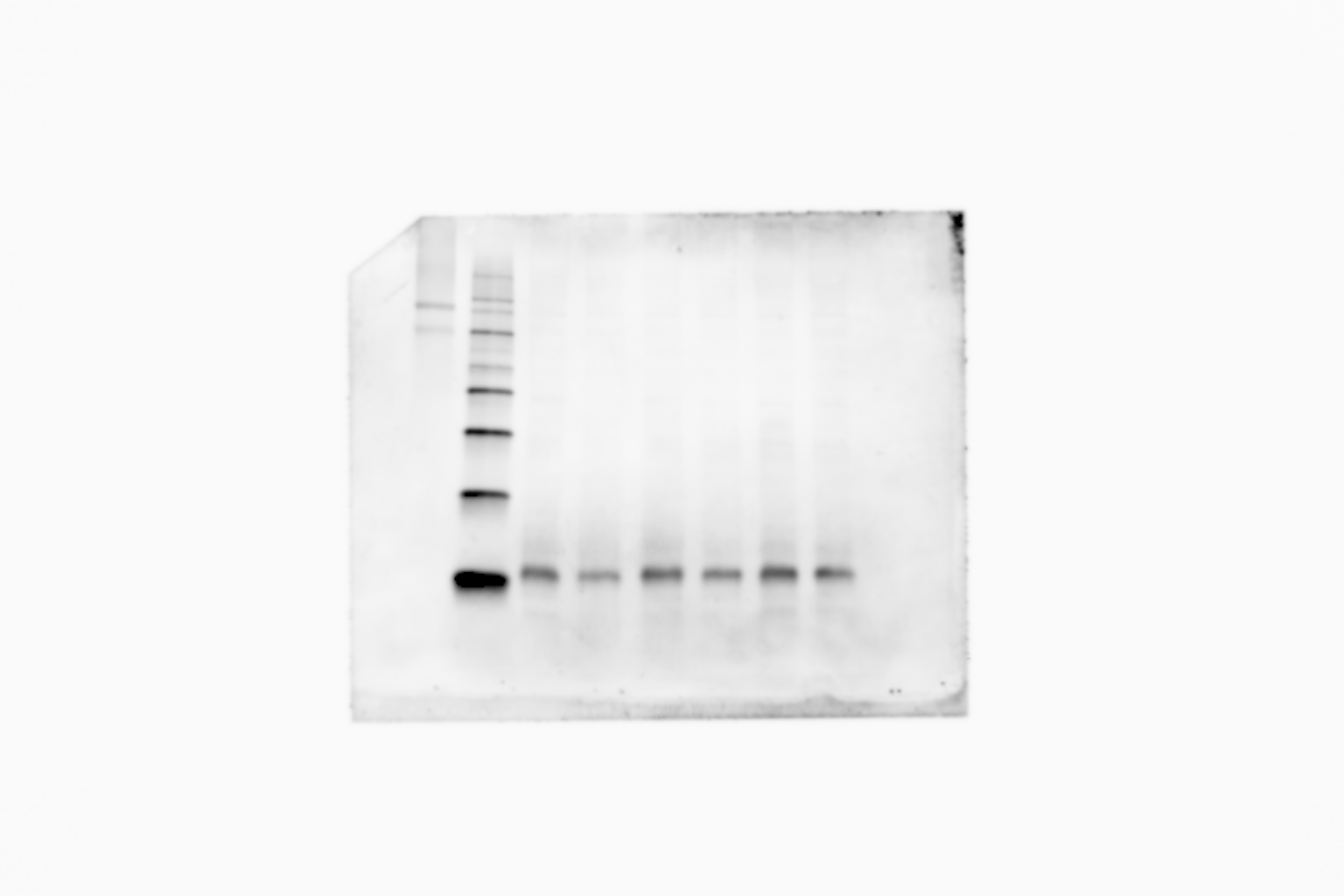

Supplement: S4 File — (ZIP) [file pone.0231910.s004.zip › S4_File/B/TIMP-2/WBexposure (each 10sec)/Fig4B.TIMP2.80s.tif]

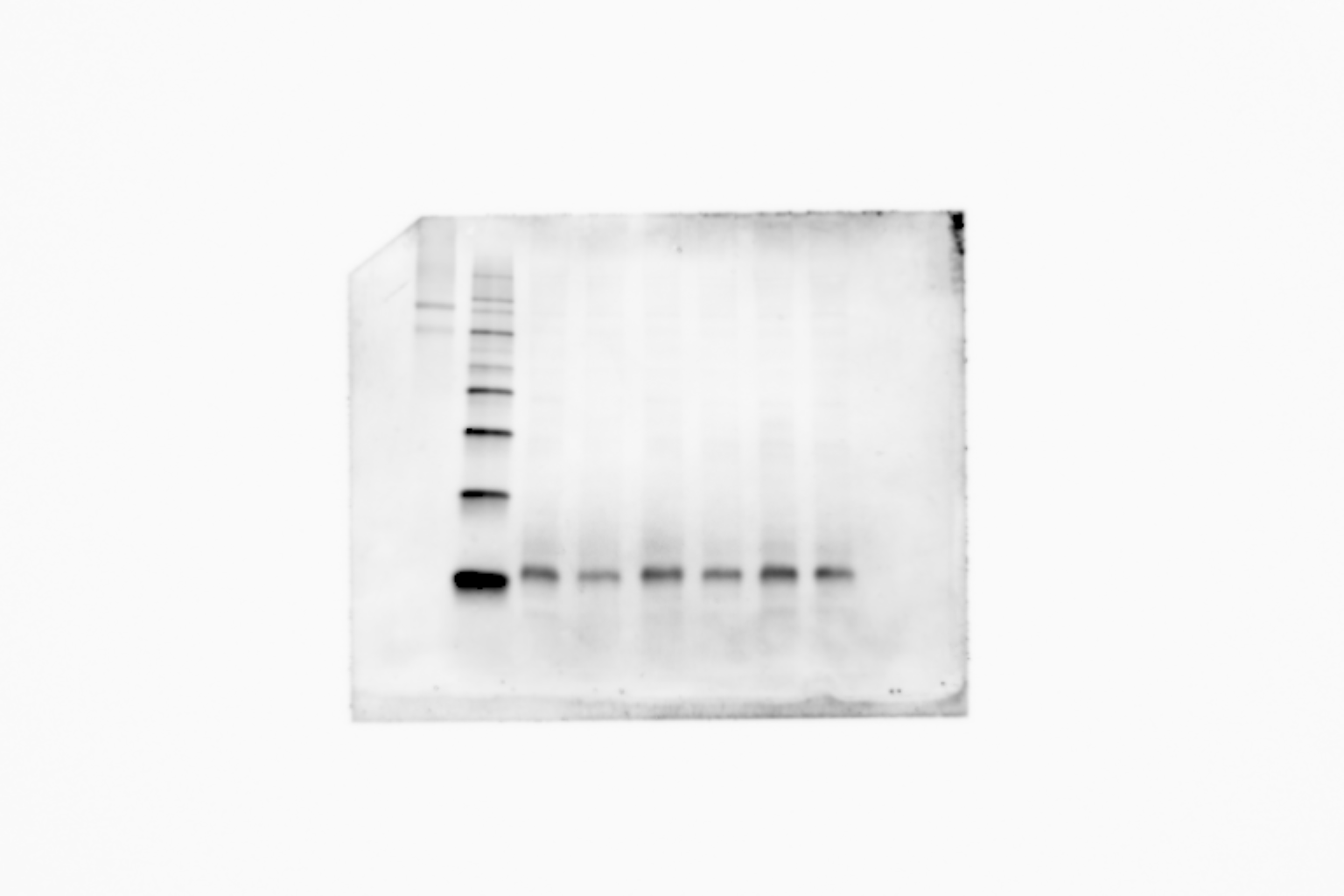

Supplement: S4 File — (ZIP) [file pone.0231910.s004.zip › S4_File/B/TIMP-2/WBexposure (each 10sec)/Fig4B.TIMP2.90s.tif]

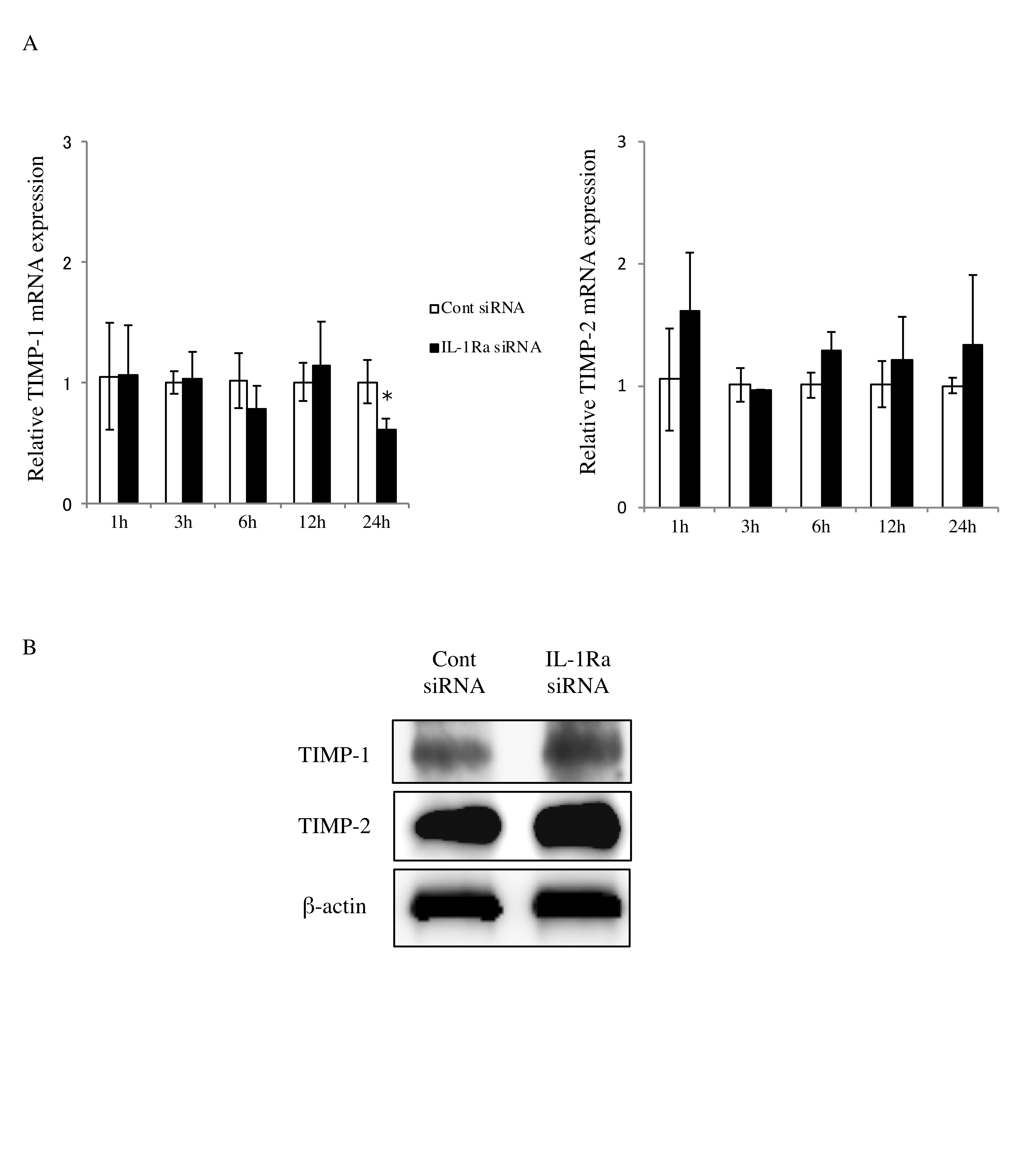

Supplement: S4 File — (ZIP) [file pone.0231910.s004.zip › S4_File/Fig4 new/Fig4 new.tiff]

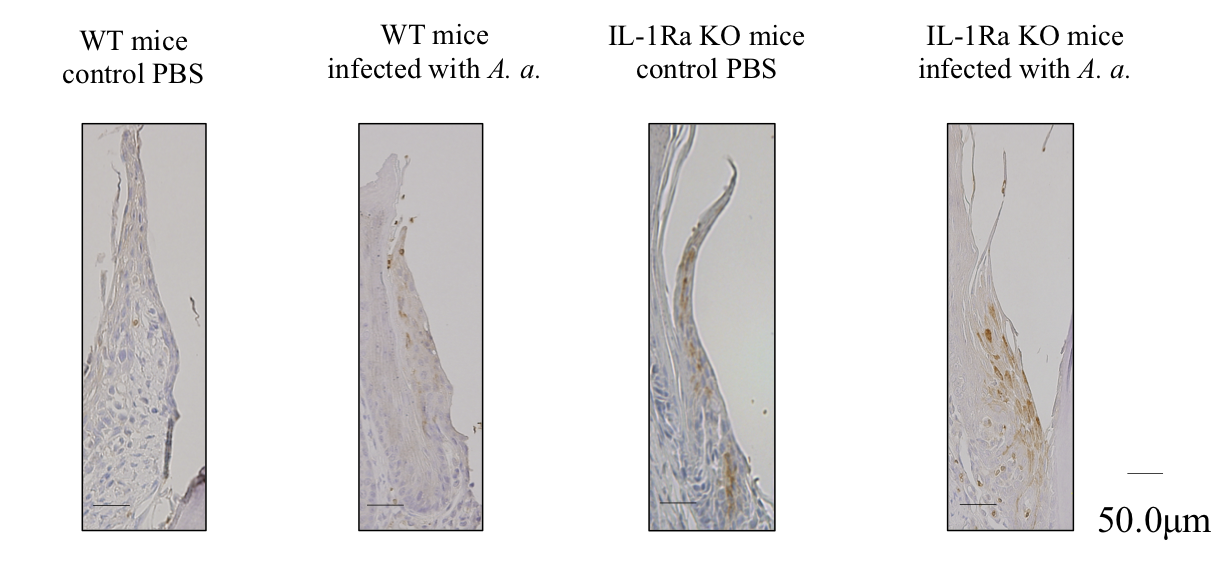

Supplement: S7 File — (ZIP) [file pone.0231910.s007.zip › S7_File/B/Fig7B.tiff]

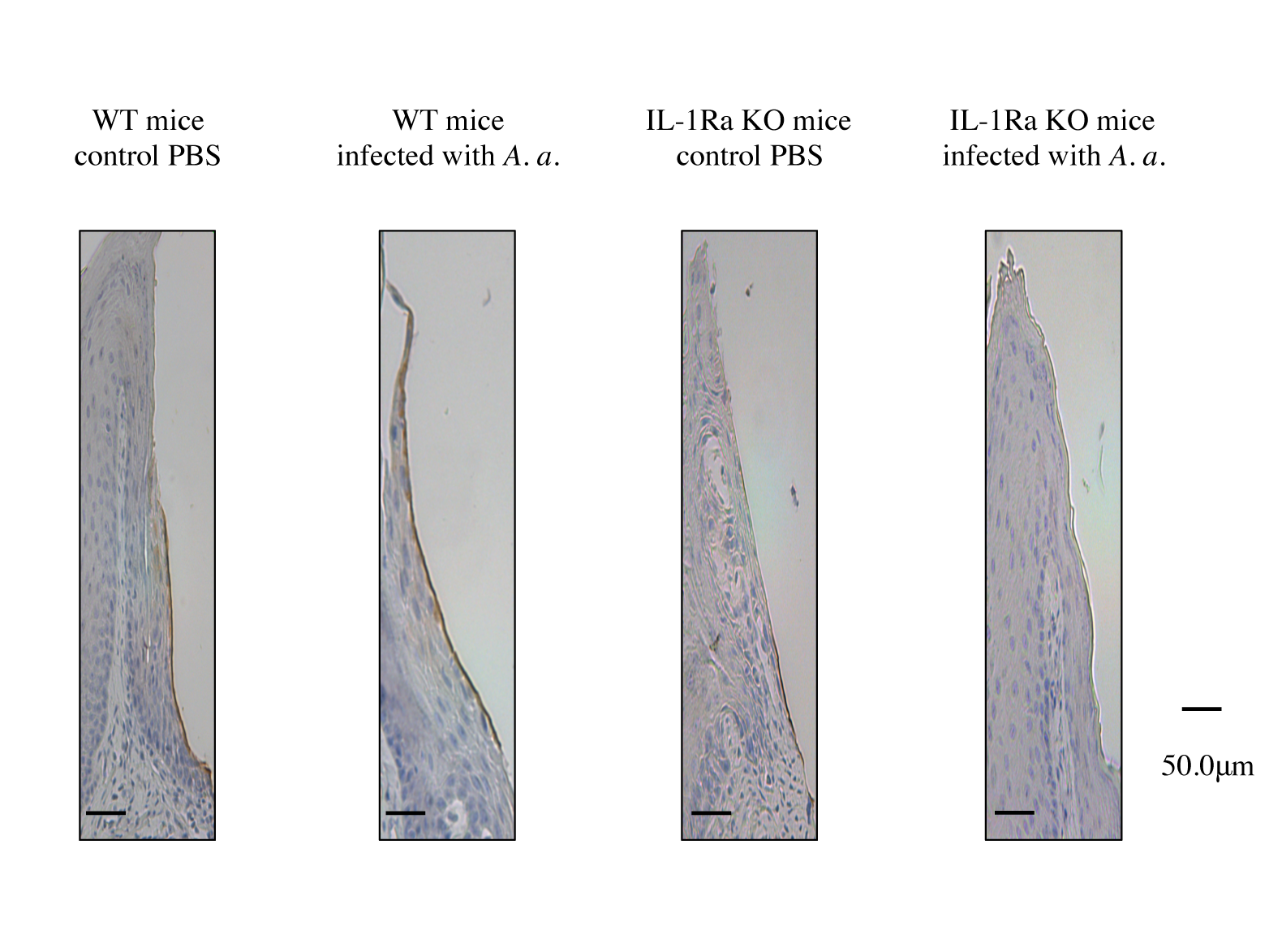

Supplement: S8 File — (ZIP) [file pone.0231910.s008.zip › S8_File/Fig8.tif]
